# Constitutive Androstane Receptor Regulates Germ Cell Homeostasis, Sperm Quality, and Male Fertility via Akt‐Foxo1 Pathway

**DOI:** 10.1002/advs.202402082

**Published:** 2024-09-24

**Authors:** Mélusine Monrose, Hélène Holota, Guillaume Martinez, Christelle Damon‐Soubeyrand, Laura Thirouard, Emmanuelle Martinot, Edwige Battistelli, Angélique de Haze, Stéphanie Bravard, Christelle Tamisier, Françoise Caira, Charles Coutton, Anne‐Laure Barbotin, Angèle Boursier, Laila Lakhal, Claude Beaudoin, David H. Volle

**Affiliations:** ^1^ INSERM U1103 Université Clermont Auvergne CNRS UMR‐6293 GReD Institute Team‐Volle Clermont‐Ferrand F‐63001 France; ^2^ CHU Grenoble Alpes UM de Génétique Chromosomique Grenoble F‐38000 France; ^3^ Team Genetics Epigenetics and Therapies of Infertility Institute for Advanced Biosciences University Grenoble Alpes INSERM U1209, CNRS UMR 5309 Grenoble F‐38000 France; ^4^ INSERM U1103 Université Clermont Auvergne CNRS UMR‐6293 GReD Institute Plateform Anipath Clermont‐Ferrand F‐63001 France; ^5^ CHU Lille Institut de Biologie de la Reproduction‐Spermiologie‐CECOS Lille F‐59000 France; ^6^ Inserm UMR‐S 1172 Laboratory of Development and Plasticity of the Neuroendocrine Brain Lille F‐59000 France; ^7^ INRAe UMR1331 ToxAlim University of Toulouse Toulouse F‐31027 France

**Keywords:** constitutive androstane receptor, Foxo1, repro‐toxicity, spermatogonia, xenobiotic

## Abstract

Male sexual function can be disrupted by exposure to exogenous compounds that cause testicular physiological alterations. The constitutive androstane receptor (Car) is a receptor for both endobiotics and xenobiotics involved in detoxification. However, its role in male fertility, particularly in regard to the reprotoxic effects of environmental pollutants, remains unclear. This study aims to investigate the role of the Car signaling pathway in male fertility. In vivo, in vitro, and pharmacological approaches are utilized in wild‐type and *Car*‐deficient mouse models. The results indicate that Car inhibition impaired male fertility due to altered sperm quality, specifically histone retention, which is correlated with an increased percentage of dying offspring in utero. The data highlighted interactions among Car, Akt, Foxo1, and histone acetylation. This study demonstrates that Car is crucial in germ cell homeostasis and male fertility. Further research on the Car signaling pathway is necessary to reveal unidentified causes of altered fertility and understand the harmful impact of environmental molecules on male fertility and offspring health.

## Graphical abstract

This study highlights the role of the constitutive androstane receptor (Car), a receptor of endo and xeno‐biotics, in testicular pathophysiology. The results indicate that Car inhibition impaired male fertility due to altered sperm quality, specifically histone retention, which is correlated with offspring's survival. This work demonstrates that Car is crucial in germ cell homeostasis via interactions among Car, Akt, Foxo1, and histone acetylation.

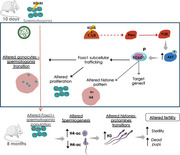

## 1 Introduction

Infertility affects ≈15% of couples worldwide,^[^ ^1^ ^]^ with male factors involved in ≈30–50% of cases.^[^ ^2^ ^]^ Male fertility relies on spermatogenesis, where spermatogonia develop into spermatocytes, then spermatids, and finally spermatozoa.^[^ ^3^, ^4^ ^]^ Spermatogenesis depends on the balance between the self‐renewal and differentiation of spermatogonial stem cells (SSCs).^[^ ^4^ ^]^ Germ cells play a crucial role in transmitting hereditary information,^[^ ^5^, ^6^ ^]^ depending on proper differentiation. During spermiogenesis, germ cells undergo major morphological changes and chromatin compaction to prevent alterations to their paternal genome.^[^ ^7^, ^8^ ^]^ This is associated with histone‐protamine transition, which plays a critical role in germ cell integrity. Defects in the histone‐protamine transition have been linked to male fertility disorders in epidemiological studies and animal models.^[^ ^8^ ^]^

Male fertility has been demonstrated in epidemiological studies and animal research to be affected by genetic mutants or exposure to environmental pollutants.^[^ ^9^, ^10^, ^11^ ^]^ Several signaling pathways mediate these effects.^[^ ^12^, ^13^, ^14^ ^]^ Among these, the constitutive androstane receptor (Car/Nr1i3), a member of the nuclear receptor superfamily, plays a key role in detoxifying endogenous and xenobiotic substances.^[^ ^15^, ^16^ ^]^ While Car is predominantly expressed in the liver, it has been detected in other organs, including the duodenum, brain, heart, and kidneys. In recent decades, Car has emerged as a crucial regulator of liver physiology, controlling metabolic and physiological processes.^[^ ^16^, ^17^, ^18^, ^19^ ^]^ Modulation of Car's transcriptional activity is linked to liver pathologies following acute or chronic exposures to environmental molecules, such as pesticides.^[^ ^18^ ^]^ The relationship between Car and xenobiotics is complex, as Car function loss can either increase or decrease an organism's sensitivity to a particular xenobiotic.^[^ ^20^ ^]^ This complexity reflects the diverse mechanisms by which Car‐regulating molecules act.

Car activity is modulated by environmental pollutants or clinical drugs that are either direct or indirect Car activators.^[^ ^21^ ^]^ Various molecules, including pesticides, fire retardants, environmental contaminants, drugs, and industrial chemicals, such as phenobarbital (barbiturate), chlorpromazine (antiemetic), acetaminophen (anti‐inflammatory drugs), and TCPOBOP (insecticide), modulate Car activity in humans and other animals.^[^ ^18^, ^19^, ^20^, ^21^ ^]^ The high ligand‐independent activity of Car makes it a good target for inverse agonists (IAs).^[^ ^27^, ^28^, ^29^, ^30^, ^31^ ^]^ Several endogenous molecules, such as 5*α*‐androst‐16‐en‐3*α*‐ol (androstenol) and 5*α*‐androstan‐3*α*‐ol (androstanol), have been identified as Car inverse agonists.^[^ ^22^, ^32^, ^33^, ^34^ ^]^ Studies have also identified numerous xenobiotics with inverse agonistic, including clotrimazole, CINPA1, 4‐butylphenol, phenolphthalein, nigramide J, and meclizine.^[^ ^24^, ^26^, ^30^, ^35^, ^36^, ^37^, ^38^ ^]^

Structurally, similar to other nuclear receptors, Car is a modulatory protein composed of several functional domains: an N‐terminal ligand‐independent transactivation domain (activation function 1; AF1), a central DNA‐binding domain that determines target gene selectivity, a dimerization interface for interaction with the retinoid‐X‐receptor, a C‐terminal ligand‐binding domain containing the ligand‐binding pocket, and a ligand‐dependent transactivation domain (activation function 2; AF2). However, unlike other nuclear receptors, Car has intrinsic constitutive activity that requires specific regulatory mechanisms aside from ligand binding. Car features a small X‐helix that orients the H12 helix of the AF2 domain into an active conformation. This H12 helix lacks a C‐terminal extension, leaving a free carboxylate group that stabilizes the active conformation even in the absence of a ligand and facilitates the recruitment of coactivators, contributing to its constitutive activity.^[^ ^39^ ^]^

The first study on Car's role showed that its constitutive activity is inhibited by steroidal metabolites, such as androstenol and androstanol.^[^ ^33^ ^]^ That study showed that the effects of these natural molecules are stereospecific, with only certain forms of androstanes (3*α*‐hydroxy, 5*α*‐reduced) being effective. These steroids do not interfere with receptor heterodimerization or DNA binding^[^ ^33^ ^]^ but cause the release of coactivators from the ligand‐binding domain. Inverse agonist binding destabilizes the H12 helix of the AF2 domain, preventing it from adopting its active conformation and leading to corepressors recruitment.^[^ ^39^ ^]^ The effects of IAs on Car in the cytoplasmic compartment are not well‐described, indicating that Car receptor activity depends on a complex signaling system that is only partially understood.

Androstenols, which can act as Car IAs, have been found in the testes of boars, pigs, and humans.^[^ ^40^ ^]^ A key step in androstenol synthesis is androstadienol conversion, which can then be sequentially converted into androstenol, a precursor of androstanol.^[^ ^41^ ^]^ In mouse testes, Car IAs have been detected in the context of altered bile acid homeostasis^[^ ^23^, ^42^ ^]^ and have been associated with deleterious effects within the testis.^[^ ^42^ ^]^ Analyses of published single‐cell data have shown that Car is expressed in human spermatogonia.^[^ ^43^ ^]^ However, the impact of Car signaling pathways on male fertility has not been fully explored, even under physiological conditions. Despite the association of xenobiotics with male fertility disorders, the role of Car in testicular pathophysiology and fertility remains under‐researched. This is a critical issue as environmentally induced diseases have increased in recent years, correlating with the ongoing decline in sperm count, sperm quality, and male fertility. Therefore, we aimed to decipher the role of Car in testicular physiology and male fertility using a combination of in vivo, in vitro, and pharmacological approaches.

## 2 Results

### 2.1 Car was Expressed in the Mouse Testis from Early Life to Adulthood

**Figure 1:**
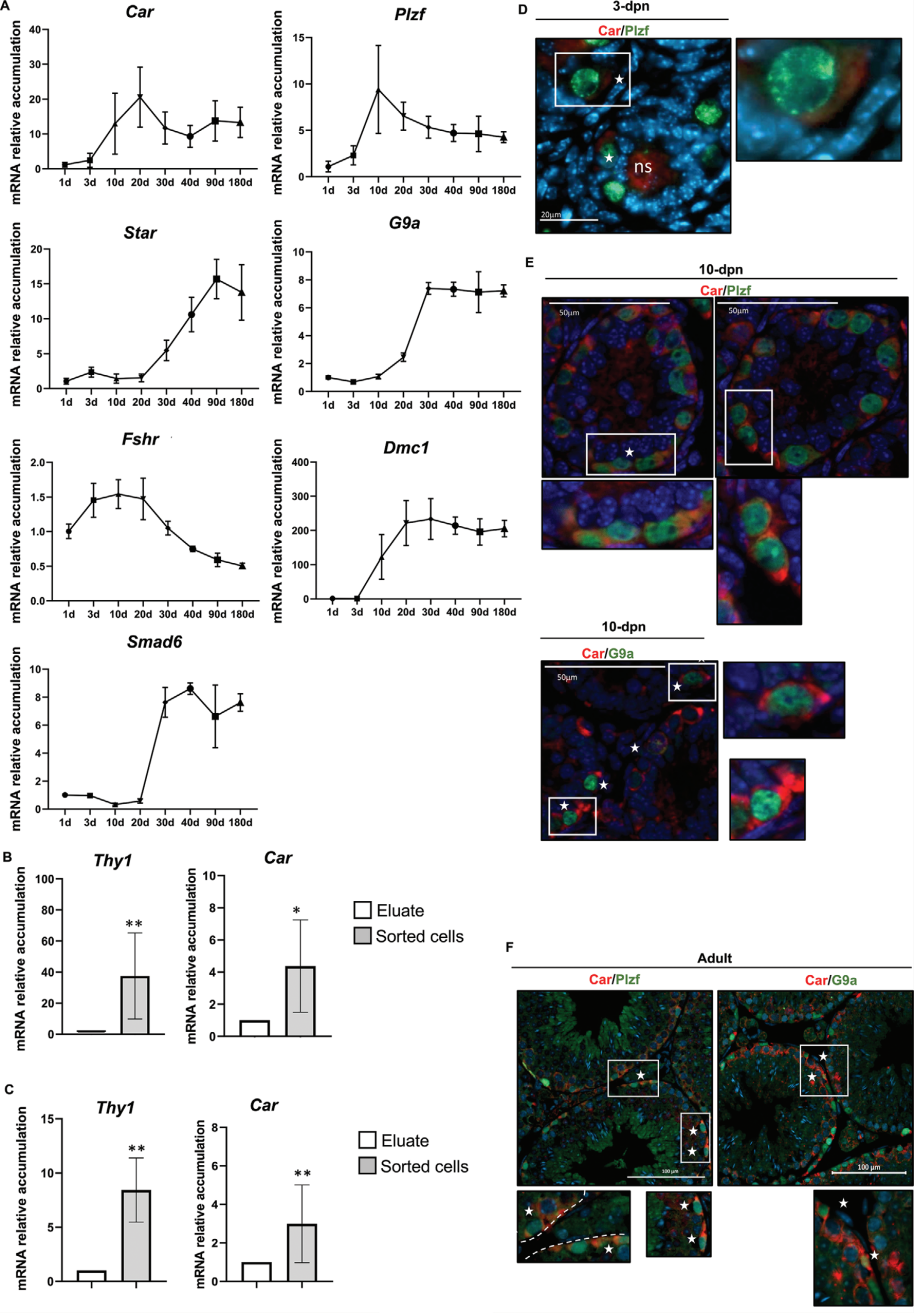
Constitutive androstane receptor is expressed in mouse testes. A) Testicular relative mRNA accumulations, normalized to β‐actin, of *Car* and somatic cell markers *Star* (Leydig) and *Fshr* (Sertoli) as well as germ cell markers *Plzf* (undifferentiated spermatogonia), *G9a* (undifferentiated/differentiating spermatogonia), *Dmc1* (spermatocyte) and *Smad6* (spermatid) at 1, 3, 10, 20, 30, 40, 90, and 180‐d‐old. n = 5 per age. B) *Thy1* and *Car* mRNA accumulations normalized to β ‐actin in Thy1+ cells or eluates isolated from 6‐d‐old C57Bl6J males. n = 7 per group. The Thy1‐ unsorted cell group was arbitrarily set at 1. Data are expressed as the means ± SD. T‐test statistical analysis; *p < 0.05 and **p < 0.01 versus Thy1‐ unsorted cell group. C) *Thy1* and *Car* mRNA accumulations normalized to β ‐actin in Thy1+ cells or eluates isolated from 3‐month‐old adult C57Bl6J males. n = 10. The Thy1‐ unsorted cell group was arbitrarily set at 1. Data are expressed as the means ± SD. T‐test statistical analysis; **p < 0.01. versus Thy1‐ unsorted cell group D‐F) Representative testis micrographs of 3 d‐postnatal (dpn), 10 dpn, or adult (8 months) Wt males stained for Car (red) and for undifferentiated spermatogonia marker Plzf (green) or for differentiating spermatogonia marker G9a (Green). White stars indicate the co‐stained cells. ns: nonspecific. The white squares delimit the area that has been enlarged. The experiment was performed on three different male mice.

To understand Car's role in male reproductive function, we first analyzed its expression pattern in mouse testes. Our qPCR data showed that *Car* mRNA was detected in mouse testes from birth (1 day postnatally) to adulthood (6 months of age) (**Figure** **1**A). Among all the testicular cell type markers analyzed—representing undifferentiated germ cells (promyelocytic leukemia zinc finger, *Plzf*, encoded by the BTB domain zinc finger containing‐16 [*Zbtb16*] gene), differentiating/differentiated spermatogonia (*G9a*, also known as eukaryotic histone methyltransferase 2), spermatocytes (*Dmc1*, DNA meiotic recombinase 1), spermatids (*Smad6*, SMAD family member 6), Leydig cells (*Star*, steroidogenic acute regulatory protein), and Sertoli cells (*Fshr*, follicle‐stimulating hormone receptor)—*Car* mRNA accumulation was closest to that of *Plzf* mRNA (Figure 1A). This revealed that *Car* mRNA is expressed in undifferentiated spermatogonia, consistent with published single‐cell data showing *Car* expression in human spermatogonia, and pseudotime analyses identifying it as part of a group of genes corresponding to undifferentiated germ cells.^[^ ^43^ ^]^ Further studies on *Car* mRNA expression in germ cells were conducted using Thy1‐positive (Thy1+) sorted spermatogonia and comparing it with the eluate. In both neonatal (6 d postnatal, dpn) and adult (3 months of age) mouse testes, *Car* mRNA was enriched in Thy1+ spermatogonial cells compared to the eluate (Figure 1B,C).

The Car protein expression in the germ cell lineage was confirmed via immunochemistry in neonatal (at 3 dpn, Figure 1D, and at 10 dpn, Figure 1E) and adult (8 months; Figure 1F) mouse testes. The results were validated using a control with no primary antibody on wild‐type (Wt) testes and testes samples of *Car* ^−/−^ males (Figure S1, Supporting Information). Car expression was detected in undifferentiated germ cells stained for Plzf (Figure 1D,E,F). Starting at 10 dpn, Car expression was also observed in differentiating/differentiated germ cells stained with G9a (Figure 1E,F).

At all ages analyzed, no Car staining was observed in Sertoli cells, which were detected using Sry‐Box transcription factor‐9 (Sox9) (Figure S2A, Supporting Information). At 3 and 10 dpn (Figure 1D,E), Car protein was detected in the interstitial compartment and, specifically in some Leydig cells, as revealed by the co‐staining of Car with 3‐*β*‐hydroxysteroid dehydrogenase (Figure S2B, Supporting Information). However, analysis of Car protein in the interstitial compartment of adult mice was challenging due to autofluorescence observed in Leydig cells (data not shown). Consistent with the in vivo data, the expression of *Car* mRNA in the germ and Leydig cells was confirmed via RT‐PCR using cDNA from the mouse MLTC1 Leydig cell line and mouse C18‐4 and GC1spg germ cell lines (Figure S2C, Supporting Information). These data highlight that Car is present at different stages of mouse testis development, from neonatal age to adulthood.

### 2.2 Car Inhibition Altered Neonatal Spermatogonia Homeostasis

To decipher the roles of Car signaling in male reproductive capacity, we analyzed the effects of the absence of the *Car* gene and/or the pharmacological inhibition of its activity. For this purpose, wild type (Wt) and *Car* knockout (*Car^−/−^*)^[^ ^18^ ^]^ male mice were exposed to either a vehicle or an IA, such as androstanol,^[^ ^33^, ^42^ ^]^ from 1 to 10 d after birth. Testicular homeostasis was then analyzed at various time points from 1 dpn to adulthood (8 months of age).

**Figure 2:**
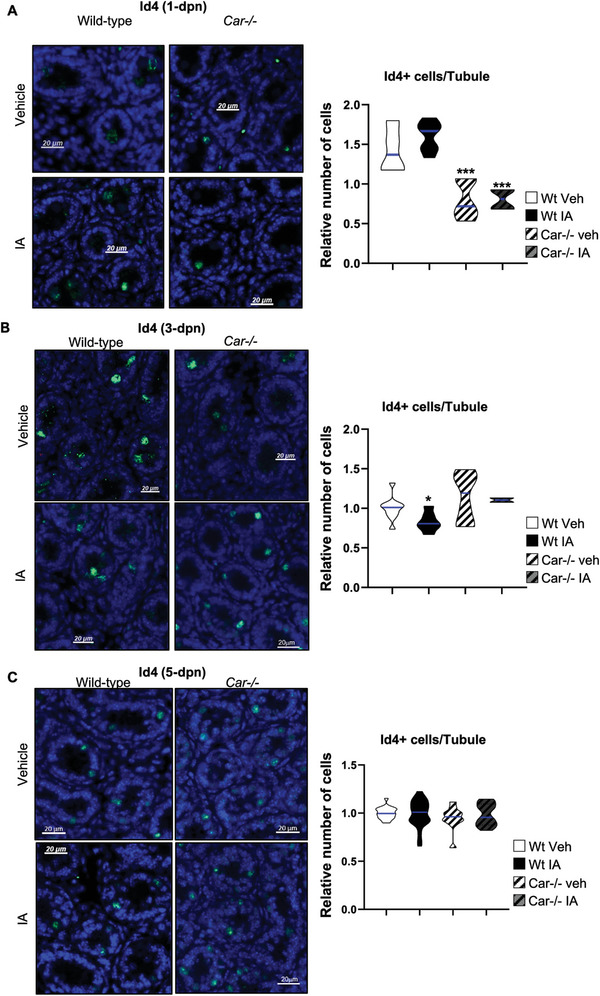
Car inhibition alters Id4+ spermatogonia population A) (Left panel) Representative testis micrographs of 1‐d‐old vehicle or IA‐treated Wt and Car^−/−^ males stained for Id4. (Right panel) Quantification of the number of Id4 positive cells per seminiferous tubule in testes of Wt or Car^−/−^ males treated with vehicle or IA. n = 5 per group. The blue line indicates the median of the group. Numbers were normalized to vehicle‐treated Wt group, which was arbitrarily set at 1. B) (Left panel) Representative testis micrographs of 3‐d‐old vehicle or IA‐treated Wt and Car^−/−^ males stained for Id4. (Right panel) Quantification of the number of Id4 positive cells per seminiferous tubule in testes of Wt or Car^−/−^ males treated with vehicle or IA. n = 5 per group. The blue line indicates the median of the group. Numbers were normalized to vehicle‐treated Wt group, which was arbitrarily set at 1. C) (Left panel) Representative testis micrographs of 5‐d‐old vehicle or IA‐treated Wt and Car^−/−^ males stained for Id4. (Right panel) Quantification of the number of Id4 positive cells per seminiferous tubule in testes of Wt or Car^−/−^ males treated with vehicle or IA. n = 20 per groups for Wt and n = 15 per groups for Car^−/−^ males. The blue line indicates the median of the group. Numbers were normalized to vehicle‐treated Wt group, which was arbitrarily set at 1. In all panels, Veh: vehicle and IA: Inverse agonist. Two‐way ANOVA followed by Holm‐Sidak's test was used for multiple comparisons. *p < 0.05; ***p < 0.001 versus vehicle‐treated Wt males.

First, we analyzed the impact of Car inhibition on neonatal testes. The data showed that the population of undifferentiated spermatogonia was altered by genetic or pharmacological inhibition of Car during the neonatal period, as confirmed by Id4 staining (**Figure** **2**). The initial effect was noted on the first day (1 dpn), with fewer Id4+ cells observed in *Car* ^−^ *^/^* ^−^ males than in Wt males (Figure 2A). At this age, IA treatment had not yet shown an effect, which was expected as treatment started at 1 dpn. At 3 dpn, fewer Id4+ cells were observed in IA‐treated Wt males compared to vehicle‐treated Wt males, whereas no difference was observed between vehicle‐treated Wt and vehicle‐treated Car^−^ *^/^* ^−^ males (Figure 2B). At 5 dpn, the number of Id4+ cells did not differ between IA‐treated Wt males and vehicle‐treated *Car* ^−^ *^/^* ^−^ males compared to vehicle‐treated Wt males (Figure 2C). Similarly, neither genetic nor pharmacological inhibition of Car significantly affected the number of Id4+ cells at 10 dpn (Figure S3, Supporting Information). These data indicate that inhibition of Car signaling leads to a transitory decrease in the number of Id4+ undifferentiated spermatogonia during the neonatal period.

The population of undifferentiated spermatogonia is heterogeneous, comprising single, paired, and aligned spermatogonia. Id4 is restricted to the early population of undifferentiated spermatogonia and is an SSC marker. In contrast, Plzf protein expression is broader, extending into later populations of undifferentiated spermatogonia. During germ cell differentiation, only a subset of undifferentiated Plzf+ spermatogonia is positive for Id4,^[^ ^44^, ^45^, ^46^ ^]^ as confirmed by our data (Figure S4A, Supporting Information). Thus, we analyzed the effect of the Car signaling pathway on the Plzf+ spermatogonial population at different time points during the neonatal period.

**Figure 3:**
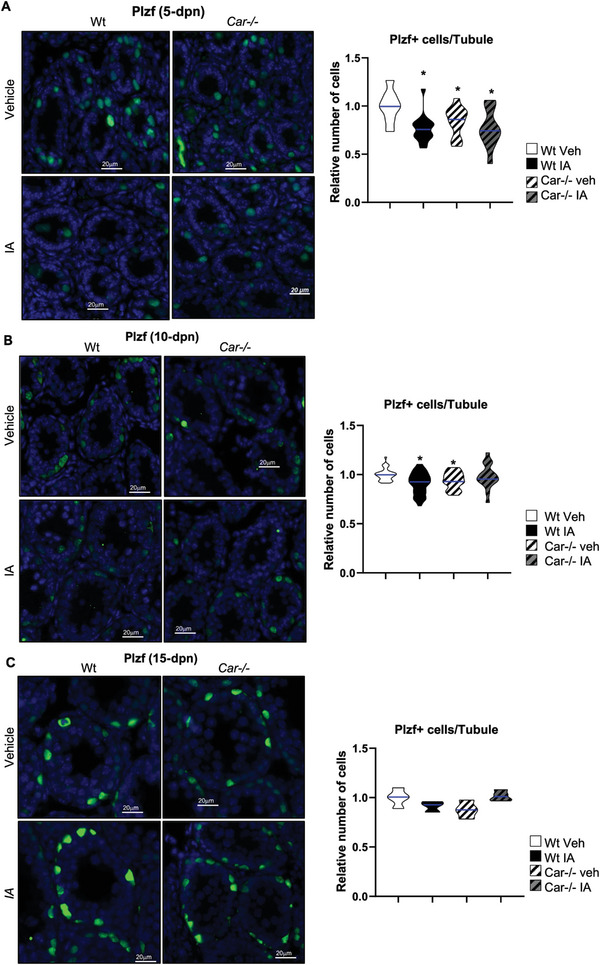
Car inhibition alters the Plzf+ spermatogonia population. A) (Left panel) Representative micrographs of 5‐d‐old IA‐treated Wt or Car^−/−^ testis stained for Plzf. (Right panel) Quantification of the number of Plzf positive cells per seminiferous tubule in testes of Wt or Car^−/−^ males treated with vehicle or IA. n = 30 per group for Wt and n = 20 per group for Car^−/−^ males. The blue line indicates the median for each group. Numbers were normalized to vehicle‐treated Wt group, which was arbitrarily set at 1. B) (Left panel) Representative micrographs of 10‐d‐old IA‐treated Wt or Car^−/−^ testis stained for Plzf. (Right panel) Quantification of the number of Plzf positive cells per seminiferous tubule in testes of Wt or Car^−/−^ males treated with vehicle or IA. n = 30 per group for Wt and n = 20 per group for Car^−/−^ males. The blue line indicates the median for each group. Numbers were normalized to vehicle‐treated Wt group, which was arbitrarily set at 1. C) (Left panel) Representative testis micrographs of 15‐d‐old vehicle or IA‐treated Wt and Car^−/−^ males stained for Plzf. (Right panel) Quantification of the number of PLZF positive cells per seminiferous tubule in testes of Wt or Car^−/−^ males treated with vehicle or IA. n = 5 per group. The blue line indicates the median for each group. Numbers were normalized to vehicle‐treated Wt group, which was arbitrarily set at 1. In all panels, Veh: vehicle and IA: Inverse agonist. Two‐way ANOVA followed by Holm‐Sidak's test for multiple comparisons. *p < 0.05; ***p < 0.001 versus vehicle‐treated Wt males.

Similar to the Id4+ cells, fewer Plzf+ cells per seminiferous tubule were observed in vehicle‐treated *Car* ^−^ *^/^* ^−^ male mice than in vehicle‐treated Wt mice at 1 dpn, with no effect of IA treatment observed (Figure S4B, Supporting Information). Notably, at 3 dpn, no difference was observed in the total number of Plzf+ cells regardless of genotype or treatment (Figure S4C, Supporting Information). Given the discrepancy between the Id4+ and Plzf+ cell populations, we analyzed the Lin28+ spermatogonial cell population, as Lin28a is a marker of undifferentiated spermatogonia with a pattern similar to Plzf in mice.^[^ ^47^ ^]^ Similar to Plzf, *Car* gene deficiency or IA‐mediated inhibition of Car activity did not affect the number of Lin28+ cells at 3 dpn (Figure S5, Supporting Information). However, at 5 dpn, the number of Plzf+ cells was lower in vehicle‐treated *Car* ^−^ *^/^* ^−^ and IA‐treated Wt males than in vehicle‐treated Wt males (**Figure** **3**A). Similarly, at 10 dpn, alterations in the number of undifferentiated Plzf+ spermatogonia were observed in *Car* ^−^ *^/^* ^−^ mice compared to Wt mice, as well as in IA‐treated Wt mice compared to vehicle‐treated Wt mice (Figure 3B). These results showed that the effects of IA were Car‐dependent, as IA had no effect in *Car* ^−^ *^/^* ^−^ male mice (Figure 3A,B).

Analysis of labeling data for Id4, Plzf, and Lin28a demonstrated the impact of Car signaling inhibition on the progression of spermatogenesis in neonatal life. Initially, the number of undifferentiated spermatogonia decreased, as revealed by Id4+ staining (1 dpn in *Car* ^−^ *^/^* ^−^ mice and at 3 dpn in response to IA treatment in Wt males), followed by a reduction in the number of Plzf+ cells at more advanced stages of the spermatogenesis at 5 and 10 dpn. This impact on spermatogenesis progression was further supported by data showing a larger decrease in the number of Plzf+ cells in the Plzf+/Id4‐ population than in the Plzf+/Id4+ population at 5 dpn in IA‐treated Wt and vehicle‐treated *Car* ^−^ *^/^* ^−^ mice compared to that in the vehicle‐treated Wt mice (Figure S6, Supporting Information).

Notably, no effect on the number of Plzf+ cells was observed in vehicle‐treated *Car* ^−^ *^/^* ^−^ or IA‐treated wild‐type mice compared to that in the vehicle‐treated Wt mice at 15 dpn (Figure 3C), suggesting normalization of the undifferentiated spermatogonia population over time. To monitor the dynamics of spermatogenesis in the context of Car signaling modulation, we studied G9a, a marker of germ cells from A1 spermatogonia to leptotene spermatocytes, which overlaps with that of Plzf+ and Ckit+ spermatogonia.^[^ ^48^ ^]^ These differentiating/differentiated spermatogonia appear approximately at 6 d postnatally in mouse testes,^[^ ^49^ ^]^ which was confirmed by our data using co‐staining of G9a and Plzf or Ckit (Figure S7A, Supporting Information). Interestingly, as with Plzf staining, no impact of Car inhibition was observed on G9a+ cells at 15 dpn (Figure S7B,C, Supporting Information). These data support the normalization of the spermatogonia population over time.

Cell proliferation and apoptosis were then analyzed to investigate the mechanisms involved in the transitory loss of Id4+ and Plzf+ spermatogonia. At 5 and 10 dpn, no effect was observed on germ cell proliferation (as revealed by Pcna staining, Figure S8A, Supporting Information). In contrast, a higher number of apoptotic germ cells (as revealed by positive TUNEL staining) was observed in the seminiferous tubules of IA‐treated Wt males and vehicle‐treated *Car* ^−^ *^/^* ^−^ males than in vehicle‐treated Wt males (Figure S8B, Supporting Information). This finding may partially explain the transient decrease in the spermatogonia population following Car inhibition, as no further effect on apoptosis was observed at 10 dpn.

### 2.3 Car Inhibition Delayed Germ Cell Differentiation and Migration to the Basement Membrane

**Figure 4:**
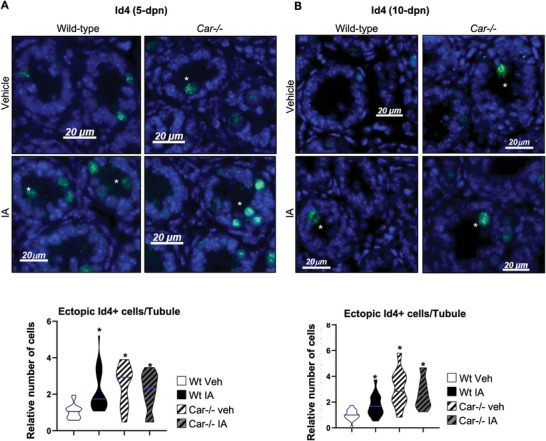
CAR inhibition alters Id4+ cell localization in neonatal mouse testis. A) (Left panel) Representative micrographs of 5‐d‐old IA‐treated Wt and Car^−/−^ testis stained for Id4. The white stars indicate ectopic cells. (Right panel) Quantification of the number of Id4 positive ectopic cells per seminiferous tubule in testes of Wt or Car^−/−^ males treated with vehicle or IA. n = 20 per group for Wt and n = 15 per group for Car^−/−^ males. The blue line indicates the median for each group. Numbers were normalized to vehicle‐treated Wt group, which was arbitrarily set at 1. B) (Left panel) Representative micrographs of 10‐d‐old IA‐treated Wt and Car^−/−^ testis stained for Id4. The white stars indicate ectopic cells. (Right panel) Quantification of the number of Id4 positive ectopic cells per seminiferous tubule in testes of Wt or Car^−/−^ males treated with vehicle or IA. n = 30 per group for Wt and n = 20 per group for Car^−/−^ males. The blue line indicates the median for each group. Numbers were normalized to vehicle‐treated Wt group, which was arbitrarily set at 1. In all panels, Veh: vehicle and IA: Inverse agonist. Two‐way ANOVA followed by Holm‐Sidak's test for multiple comparisons. *p < 0.05 versus vehicle‐treated Wt males. Veh: vehicle and IA: Inverse agonist.

During postnatal development, germ cells at 6 dpn typically migrate from the center of the seminiferous tubule to the basement membrane. In addition to the transient impact of Car inhibition on spermatogonial cell numbers, we observed an alteration in cell localization within the seminiferous tubules. At 5 and 10 dpn, vehicle‐treated *Car* ^−/−^ and IA‐treated Wt male mice presented an accumulation of ectopic Id4+ cells in the center of the seminiferous tubules compared to vehicle‐treated Wt male mice (**Figure** **4**A,B). However, this ectopic Id4+ cell population represented a small percentage of total cells, and this effect diminished with age, notably decreasing between 5 dpn (30% in IA‐treated Wt and 20% in vehicle‐treated *Car* ^−/−^ mice) and 10 dpn (11% in IA‐treated Wt and 18% in vehicle‐treated *Car* ^−/−^ mice).

**Figure 5:**
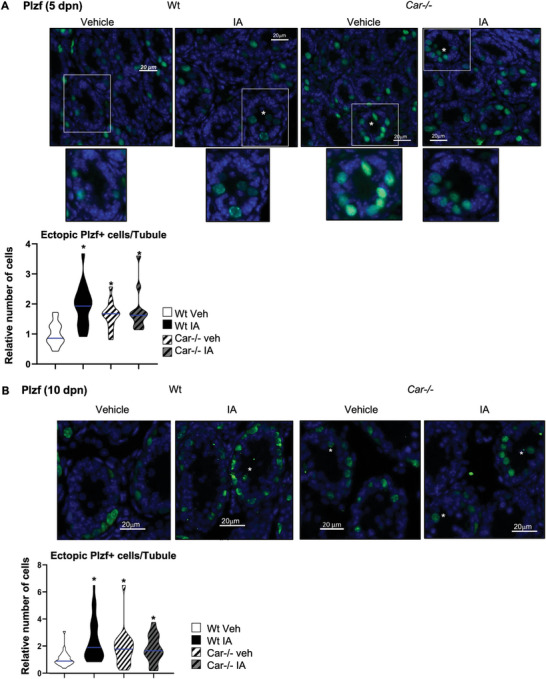
CAR inhibition alters Plzf+ cell localization in neonatal mouse testis. A) (Top panel) Representative micrographs of 5‐d‐old IA‐treated Wt and Car^−/−^ testis stained for Plzf. The white stars indicate ectopic cells. White square indicates enlarged area. (Bottom panel) Quantification of the number of Plzf positive ectopic cells per seminiferous tubule in 5‐dpn testis of Wt or Car^−/−^ males treated with vehicle or IA. n = 20 per group for Wt and n = 15 per group for Car^−/−^ males. The blue line indicates the median for each group. Numbers were normalized to vehicle‐treated Wt group, which was arbitrarily set at 1. B) (Top panel) Representative micrographs of 10‐d‐old IA‐treated Wt testis stained for Plzf. The white stars indicate ectopic cells. (Bottom panel) Quantification of the number of Plzf positive ectopic cells per seminiferous tubule in 10‐dpn testis of WT or Car^−/−^ males treated with vehicle or IA. n = 30 per group for Wt and n = 20 per group for Car^−/−^ males. The blue line indicates the median for each group. Numbers were normalized to vehicle‐treated Wt group, which was arbitrarily set at 1. In all panels, Veh: vehicle and IA: Inverse agonist. Two‐way ANOVA followed by Holm‐Sidak's test for multiple comparisons. *p < 0.05 versus vehicle‐treated Wt males.

To confirm these results, we analyzed the Plzf+ population and observed a significant accumulation of ectopic Plzf+ cells in the center of the seminiferous tubules at 5 dpn in vehicle‐treated *Car* ^−^ *^/^* ^−^ and IA‐treated Wt males compared to that in vehicle‐treated Wt males (**Figure** **5**A). This abnormal localization persisted at 10 dpn in both *Car* ^−^ *^/^* ^−^ and IA‐treated Wt males (Figure 5B), highlighting a disruption caused by Car inhibition. Notably, IA exposure had no effect in *Car* ^−^ *^/^* ^−^ males. As for Id4+ cells, the impact of Car inhibition on spermatogonia localization was transient. Indeed, at 15 dpn, no ectopic Plzf+ cells was observed in the center of the seminiferous tubules in either vehicle‐treated *Car* ^−^ *^/^* ^−^ or IA‐treated Wt mice (Figure 3C).

Similarly, the transient impact of Car genetic or pharmacological inhibition on germ cell localization was confirmed through analysis of G9a+ cells. At 10 dpn, an increased number of ectopic G9a+ cells was observed in vehicle‐treated *Car* ^−^ *^/^* ^−^ and IA‐treated Wt males compared to that in vehicle‐treated Wt males (Figure S7B, Supporting Information). These effects were Car‐dependent, as IA had no effect in *Car* ^−^ *^/^* ^−^ males at 10 dpn. By 15 dpn, no ectopic G9a+ cells were detected in the seminiferous tubules of vehicle‐treated *Car* ^−^ *^/^* ^−^ or IA‐treated wild‐type mice, resembling the pattern observed in vehicle‐treated Wt males (Figure S7C, Supporting Information).

These data demonstrated that Car inhibition transiently disrupts the normal localization of neonatal spermatogonia, likely impacting cell migration and/or differentiation processes crucial during the gonocyte‐to‐spermatogonia transition in neonatal mouse testes.^[^ ^50^, ^51^ ^]^ To further explore the impact on the gonocyte (pro‐spermatogonia) population, we analyzed DNA‐methyl‐transferase 3l (Dnmt3l) staining.^[^ ^52^, ^53^ ^]^ Our results confirmed the expected expression pattern of Dnmt3l in Wt testes during neonatal period (Figure S9A, Supporting Information) and showed altered early neonatal germ cell homeostasis in vehicle ‐treated *Car* ^−^ *^/^* ^−^ and IA‐treated Wt mice compared to vehicle‐treated Wt males (Figure S9B, Supporting Information). Specifically, higher numbers of Dnmt3l+ gonocytes were observed in vehicle‐treated *Car* ^−^ *^/^* ^−^ and IA‐treated Wt males at 1 dpn compared to vehicle treated Wt males (Figure S9B, Supporting Information). This altered pattern persisted at 3 dpn and 5 dpn in IA‐treated Wt mice and vehicle‐treated *Car* ^−^ *^/^* ^−^ mice compared to that in vehicle‐treated Wt males (Figure S9C,D, Supporting Information). These findings underscore that Car inhibition affects early neonatal germ cell dynamics, potentially influencing gonocyte physiology during testicular development.

### 2.4 Car Inhibition Altered Foxo1 Subcellular Localization in Spermatogonia

Foxo1 is crucial in the transition from gonocytes to spermatogonia during neonatal mouse testis development.^[^ ^20^ ^]^ Previous studies have shown interactions between Foxo1 and Car signaling pathways, influencing transcriptional activation.^[^ ^54^ ^]^ To assess the potential impact of Car on this transition, we analyzed the Foxo1 signaling pathway.

**Figure 6:**
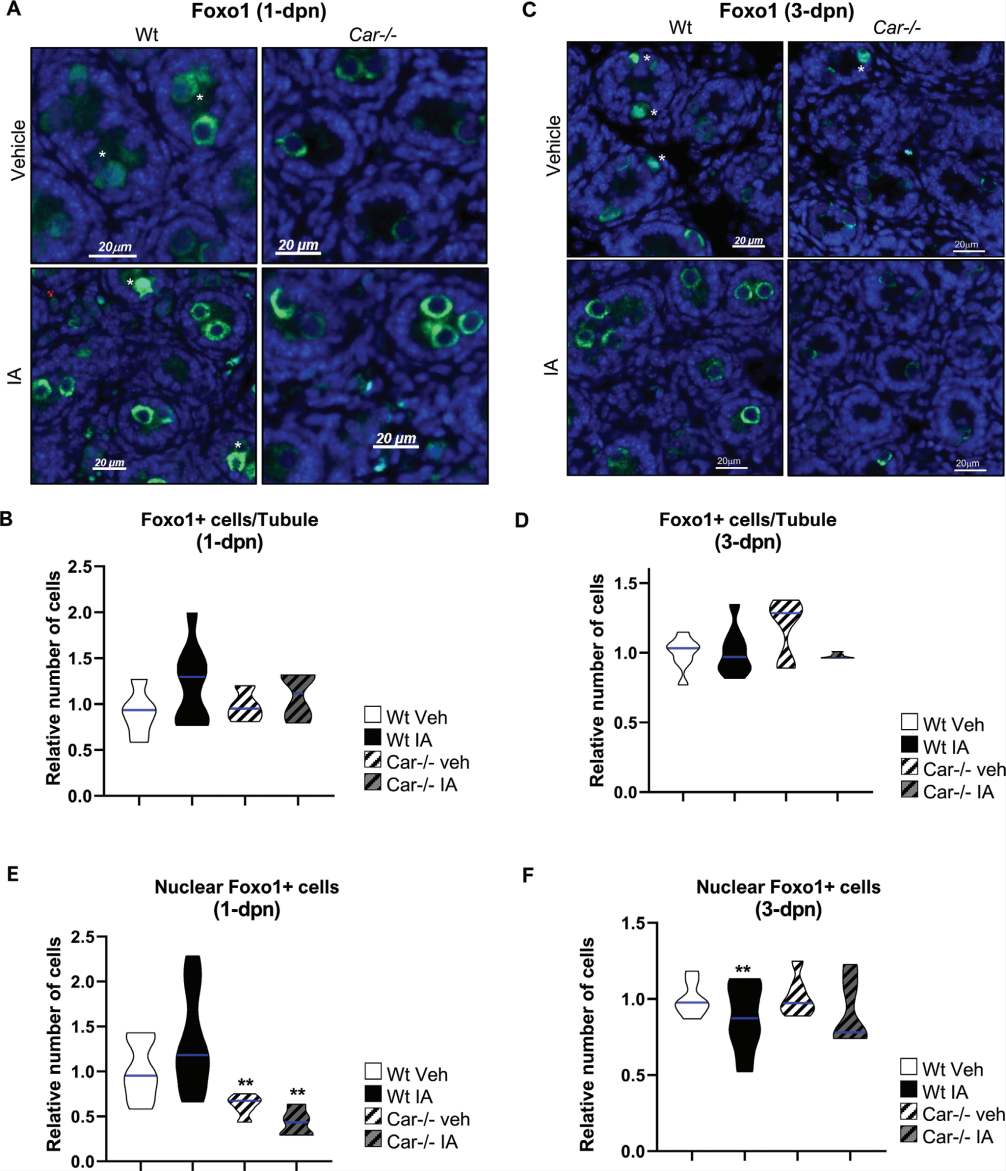
CAR inhibition alters Foxo1+ spermatogonia. A) Representative micrographs of 1‐d old vehicle or IA‐treated Wt and Car^−/−^ testis stained for Foxo1. The white stars indicate cells with Foxo1 nuclear staining. B) Quantification of the number of Foxo1‐positive cells per seminiferous tubule in 1‐d‐old testes of Wt or Car^−/−^ males treated with vehicle or IA. n = 5 per group. The blue line indicates the median for each group. Numbers were normalized to vehicle‐treated Wt group, which was arbitrarily set at 1.C) Representative micrographs of the 3‐d‐old vehicle or IA‐treated Wt and Car^−/−^ testis stained for Foxo1. The white stars indicate cells with Foxo1 nuclear staining. D) Quantification of the number of Foxo1‐positive cells per seminiferous tubule in 3‐d‐old testes of Wt or Car^−/−^ males treated with vehicle or IA. n = 5 per group. The blue line indicates the median for each group. Numbers were normalized to vehicle‐treated Wt group, which was arbitrarily set at 1. E) Quantification of the relative ratio of Foxo1‐positive cells with nuclear staining at 1‐dpn testis of Wt male mice or Car^−/−^ males. n = 5 per group. The blue line indicates the median for each group. Numbers were normalized to vehicle‐treated Wt group, which was arbitrarily set at 1. F) Quantification of the relative ratio of Foxo1‐positive cells with nuclear staining at 3‐dpn testis of Wt male mice or Car^−/−^ males. n = 5 per group. The blue line indicates the median for each group. Numbers were normalized to vehicle‐treated Wt group, which was arbitrarily set at 1. In all panels, Veh: vehicle and IA: Inverse agonist. Two‐way ANOVA followed by Holm‐Sidak's test for multiple comparisons. **p < 0.01 versus vehicle‐treated Wt males.

**Figure 7:**
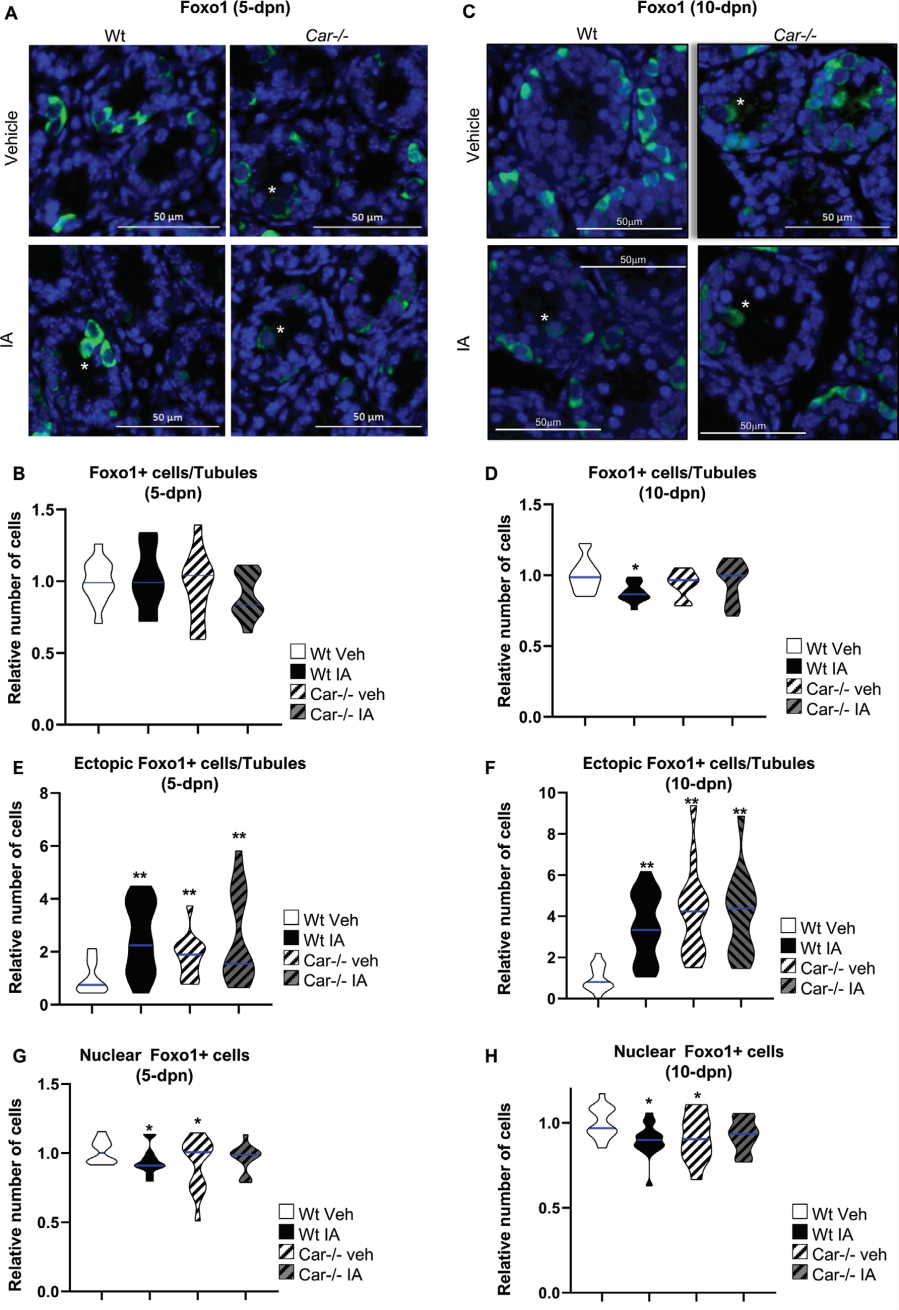
CAR inhibition alters Foxo+ cell localization in seminiferous tubules and Foxo1 subcellular localization in spermatogonia. A) Representative micrographs of the 5‐d‐old vehicle or IA‐treated Wt and Car^−/−^ testis stained for Foxo1. The white stars indicate ectopic cells. B) Quantification of the number of Foxo1‐positive cells per seminiferous tubule in 5‐dpn testis of Wt or Car^−/−^ males treated with vehicle or IA and 5 dpn. n = 20 per group for Wt and n = 15 per group for Car^−/−^ males. The blue line indicates the median for each group. Numbers were normalized to the vehicle‐treated Wt group, which was arbitrarily set at 1. C) Representative micrographs of the 10‐d‐old vehicle or IA‐treated Wt and Car^−/−^ testis stained for Foxo1. The white stars indicate ectopic cells. D) Quantification of the number of Foxo1‐positive cells per seminiferous tubule in testes of Wt or Car^−/−^ males treated with vehicle or IA at 10 dpn. n = 30 per group for Wt and n = 20 per group for Car^−/−^ males. The blue line indicates the median for each group. Numbers were normalized to the vehicle‐treated Wt group, which was arbitrarily set at 1. E) Quantification of the number of ectopic Foxo1‐positive cells per seminiferous tubule in 5‐dpn testis of Wt or Car^−/−^ males treated with vehicle or IA. n = 20 per group for Wt and n = 15 per group for Car^−/−^ males. The blue line indicates the median for each group. Numbers were normalized to the vehicle‐treated Wt group, which was arbitrarily set at 1. F) Quantification of the number of ectopic Foxo1‐positive cells per seminiferous tubule in 10‐dpn testis of Wt or Car^−/−^ males treated with vehicle or IA. n = 30 per group for Wt and n = 20 per group for Car^−/−^ males. The blue line indicates the median for each group. Numbers were normalized to the vehicle‐treated Wt group, which was arbitrarily set at 1. G) Quantification of the relative ratio of Foxo1‐positive cells with nuclear staining at 5‐dpn testis of Wt male mice or Car^−/−^ males. n = 20 per group for *WT* and n = 15 per group for Car^−/−^ males. The blue line indicates the median for each group. Numbers were normalized to vehicle‐treated Wt group, which was arbitrarily set at 1. H) Quantification of the relative ratio of Foxo1‐positive cells with nuclear staining at 10‐dpn testis of Wt male mice or Car^−/−^ males. n = 30 per group for Wt and n = 20 per group for Car^−/−^ males. The blue line indicates the median for each group. Numbers were normalized to the vehicle‐treated Wt group, which was arbitrarily set at 1. In all panels, Veh: vehicle and IA: Inverse agonist. Two‐way ANOVA followed by Holm‐Sidak's test for multiple comparisons. *p < 0.05; **p < 0.01 versus vehicle‐treated Wt males.

From 1 to 5 dpn, no differences were observed in the overall number of Foxo1+ cells between *Car* ^−^ *^/^* ^−^ males or in IA‐treated Wt males compared to that in vehicle‐treated Wt males (**Figures** **6**A–D and **7**A,B). By 10 dpn, IA‐treated Wt males showed a decreased number of Foxo1+ cells compared to vehicle‐treated Wt males, while vehicle‐treated *Car* ^−^ *^/^* ^−^ males did not differ from vehicle‐treated Wt males (Figure 7C,D). These findings suggest that early neonatal effects of Car inhibition on Id4+ or Plzf+ germ cell numbers and localizations were not linked to Foxo1+ cell numbers.

However, genetic or pharmacological Car inhibition affected Foxo1+ cell localization similar to Id4+ or Plzf+ cells. At 5 dpn, more ectopic Foxo1+ germ cells were observed at the center of the seminiferous tubules in IA‐treated Wt males and vehicle‐treated *Car* ^−^ *^/^* ^−^ males compared to vehicle‐treated Wt males (Figure 7A,E). Similarly, at 10 dpn, more ectopic Foxo1+ cells were observed in IA‐treated Wt males and vehicle‐treated *Car* ^−^ *^/^* ^−^ males than in Wt males (Figure 7C,F). At 15 dpn, similar to other germ cell markers, no ectopic Foxo1+ cells were observed in IA‐treated Wt males or vehicle‐treated *Car* ^−^ *^/^* ^−^ males (Figure S10A, Supporting Information).

Foxo1 undergoes translocation from the cytoplasm to the nucleus in early postnatal gonocytes, a process implicated in the transition from gonocytes to SSCs.^[^ ^20^ ^]^ Consistent with these findings, our data in Wt mice showed that testicular germ cells showed positive cytoplasmic Foxo1 staining in 80% of the cells at 1 dpn, 40% at 3 dpn, and 20% at 10 dpn (Figure S10B, Supporting Information).

Notably, at 1 dpn vehicle‐treated *Car* ^−^ *^/^* ^−^ males had fewer Foxo1+ cells with nuclear staining than vehicle‐treated Wt males (Figure 6E). No difference was observed between IA‐treated Wt and vehicle‐treated Wt male mice at this age, reflecting the timing of exposure on the first day of life. However, by 3 dpn, IA‐treated Wt males exhibited a lower percentage of Foxo1+ cells with nuclear staining than vehicle‐treated Wt males (Figure 6F). This altered subcellular localization of Foxo1 persisted at 5 and 10 dpn, where both IA‐treated Wt males and vehicle‐treated *Car* ^−^ *^/^* ^−^ showed a lower percentage of cells with nuclear localization of Foxo1 than vehicle‐treated Wt males (Figure 7G,H). IA treatment had no effect on Foxo1 localization in *Car* ^−^ *^/^* ^−^ mice (Figure 7G,H).

These findings suggest that after Car inhibition, either genetically (*Car* ^−^ *^/^* ^−^) or pharmacologically (IA treatment), Foxo1 accumulation was reduced in the nucleus and increased in the cytoplasm. This suggests that Car inhibition modulates Foxo1 signaling by disrupting its cellular localization, potentially impairing the cytoplasmic‐nuclear translocation process critical for the transition from gonocytes to SSCs.^[^ ^20^ ^]^ This alternation may contribute to the retention of more cells in the center of the seminiferous tubules, possibly due to disrupted differentiation and/or migration processes.

### 2.5 Neonatal Exposure to Car IA and Car Gene Deficiency Altered Testicular Histology in Adult Mice

**Figure 8:**
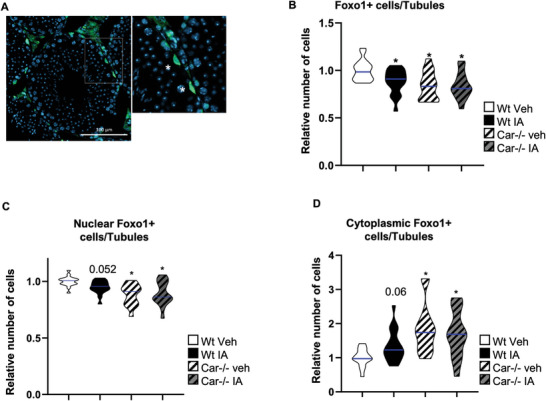
Car inhibition alters Foxo+ cells in adult testis. A) Representative micrographs of adult (8 months) IA‐treated Wt and Car^−/−^ testis stained for Foxo1. The white square indicates the enlarged area. Stars indicate Foxo1+ cells. B) Quantification of the number of Foxo1‐positive cells per seminiferous tubule in adult testes of Wt or Car^−/−^ males treated with vehicle or IA. The blue line indicates the median for each group. Numbers were normalized to the vehicle‐treated Wt group, which was arbitrarily set at 1. (n = 26 for Wt veh; n = 24 for IA, n = 15 for Car^−/−^ veh, and n = 15 for Car^−/−^ IA). C) Quantification of ratios of Foxo1+ cells with nuclear staining compared to the total number of Foxo1‐positive cells in Wt and Car^−/−^ male mice treated with vehicle or IA. (n = 26 for Wt veh; n = 24 for IA, n = 15 for Car^−/−^ veh and n = 15 for Car^−/−^ IA). The blue line indicates the median for each group. Wt vehicle group was arbitrarily set at 1. D) Quantification of ratios of Foxo1+ cells with cytoplasmic staining compared to the total number of Foxo1‐positive cells in Wt and Car^−/−^ male mice F treated with vehicle or IA. The blue line indicates the median for each group. Wt vehicle group was arbitrarily set at 1. Two‐way ANOVA followed by Holm‐Sidak's test for multiple comparisons. *p < 0.05 versus vehicle‐treated Wt males.

In contrast to the other markers tested (Plzf and G9a), the number of Foxo1+ cells remained lower in IA‐treated Wt and vehicle‐treated *Car* ^−^ *^/^* ^−^ mice than that in vehicle‐treated Wt male mice at 15 dpn (Figure S10A, Supporting Information). To determine whether this persisted into adulthood, we analyzed the long‐term effects of the Car signaling pathway in Wt and *Car* ^−^ *^/^* ^−^ male mice at 8 months of age. The number of Foxo1+ cells was still lower in vehicle‐treated *Car* ^−/−^ and IA‐treated Wt adult male mice than in vehicle‐treated Wt mice (**Figure** **8**A,B). However, IA treatment had no effect in *Car* ^−^ *^/^* ^−^ males (Figure 8A,B). As observed in neonatal mice, adult testes showed a lower rate of nuclear Foxo1 staining and a higher rate of cytoplasmic staining in *Car* ^−^ *^/^* ^−^ males than Wt mice, as well as in IA‐treated Wt males compared to vehicle‐treated Wt male mice (Figure 8C,D).

**Figure 9:**
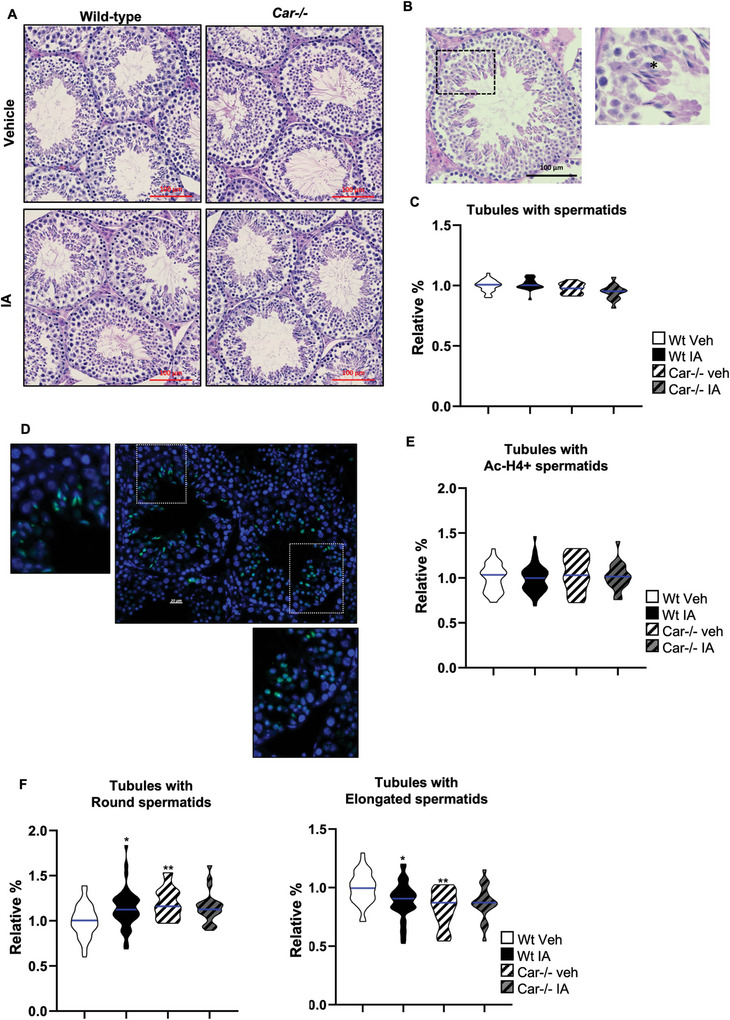
Car inhibition alters the late step of spermatogenesis. A) Representative micrographs of hematoxylin/eosin‐stained adult testes (8 months of age) of Wt or Car^−/−^ males neonatally treated with the vehicle or IA. B) Representative micrograph of hematoxylin/eosin‐stained adult testes (8 months of age) of Wt males neonatally treated with the vehicle IA. The square indicates the enlarged region. The black star indicates elongated spermatids. C) Quantification of the relative percentage of seminiferous tubules with elongated spermatids in adult testis (8 months of age) of Wt or Car^−/−^ males treated with vehicle or IA. (n = 26 for Wt veh; n = 24 for IA, n = 15 for Car^−/−^ veh and n = 15 for Car^−/−^ IA). The blue line indicates the median for each group. Numbers were normalized to vehicle‐treated Wt which was arbitrarily set at 1. D) Representative micrograph of adult (8 months of age) IA‐treated Wt testes stained for acetylated histone H4 (Ac‐H4). White squares indicate the enlarged region. Zoom area upper left shows elongated spermatids stained for Ac‐H4 and the zoom area lower right shows round spermatids stained for Ac‐H4. E) Quantification of the relative percentage of seminiferous tubules with Ac‐H4 spermatids in testis (8 months of age) of Wt or Car^−/−^ males treated with vehicle or IA. (n = 26 for Wt veh; n = 24 for IA, n = 15 for Car^−/−^ veh and n = 15 for Car^−/−^ IA). The blue line indicates the median for each group. Numbers were normalized to vehicle‐treated Wt which was arbitrarily set at 1. F) Quantification of the relative percentage of seminiferous tubules with round or elongated Ac‐H4 spermatids in testis (8 months of age) of Wt or Car^−/−^ males treated with vehicle or IA. (n = 26 for Wt veh; n = 24 for IA, n = 15 for Car^−/−^ veh and n = 15 for Car^−/−^ IA). The blue line indicates the median for each group. Numbers were normalized to vehicle‐treated Wt which was arbitrarily set at 1. In all panels, Veh: vehicle and IA: Inverse agonist. Two‐way ANOVA followed by Holm‐Sidak's test for multiple comparisons. *p < 0.05; **p < 0.01 versus vehicle‐treated Wt males.

As Foxo1 is involved in germ‐cell homeostasis,^[^ ^20^ ^]^ we analyzed the testicular histology of adult mice. However, hematoxylin and eosin staining revealed no major difference in testicular histology between the groups (**Figure** **9**A). Additionally, the number of Plzf+ spermatogonia and Sox9+ Sertoli cells did not differ in Wt mice following IA treatment compared to that in vehicle‐treated Wt mice (Figure S11A,B, Supporting Information). Similarly, no difference was observed in the number of seminiferous tubules positively stained for the meiotic marker Sycp3 (synaptonemal complex protein 3) in the testes of IA‐treated Wt males compared to that in vehicle‐treated Wt males (Figure S11C, Supporting Information). To further investigate spermatogenesis, we analyzed spermatids. No difference was observed in the percentage of seminiferous tubules with elongated spermatids in vehicle‐treated *Car* ^−^ *^/^* ^−^ and IA‐treated Wt males compared to vehicle‐treated Wt males (Figure 9B,C). A deeper analysis revealed no difference in the overall percentage of seminiferous tubules with post‐meiotic cells, as revealed by the Ac‐H4 (acetylated histone H4) staining of spermatids (Figure 9D,E). However, in IA‐treated Wt males, Ac‐H4 staining revealed fewer seminiferous tubules with elongated spermatids and a higher relative number of seminiferous tubules with round spermatids than vehicle‐treated Wt (Figure 9F). Similarly, Ac‐H4 staining also revealed fewer seminiferous tubules with elongated spermatids and a higher relative number of seminiferous tubules with round spermatids in vehicle‐treated *Car* ^−^ *^/^* ^−^ mice than vehicle‐treated Wt males (Figure 9F). These data suggest that Car inhibition was associated with alterations in spermiogenesis. Moreover, as the acetylation of H4 is considered a key event in the histone‐to‐protamine transition, these observations suggest that Car inhibition (genetically or pharmacologically) could lead to reduced efficiency in initiating this transition. This could explain the higher proportion of seminiferous tubules with round spermatids than those with elongated spermatids.

**Figure 10:**
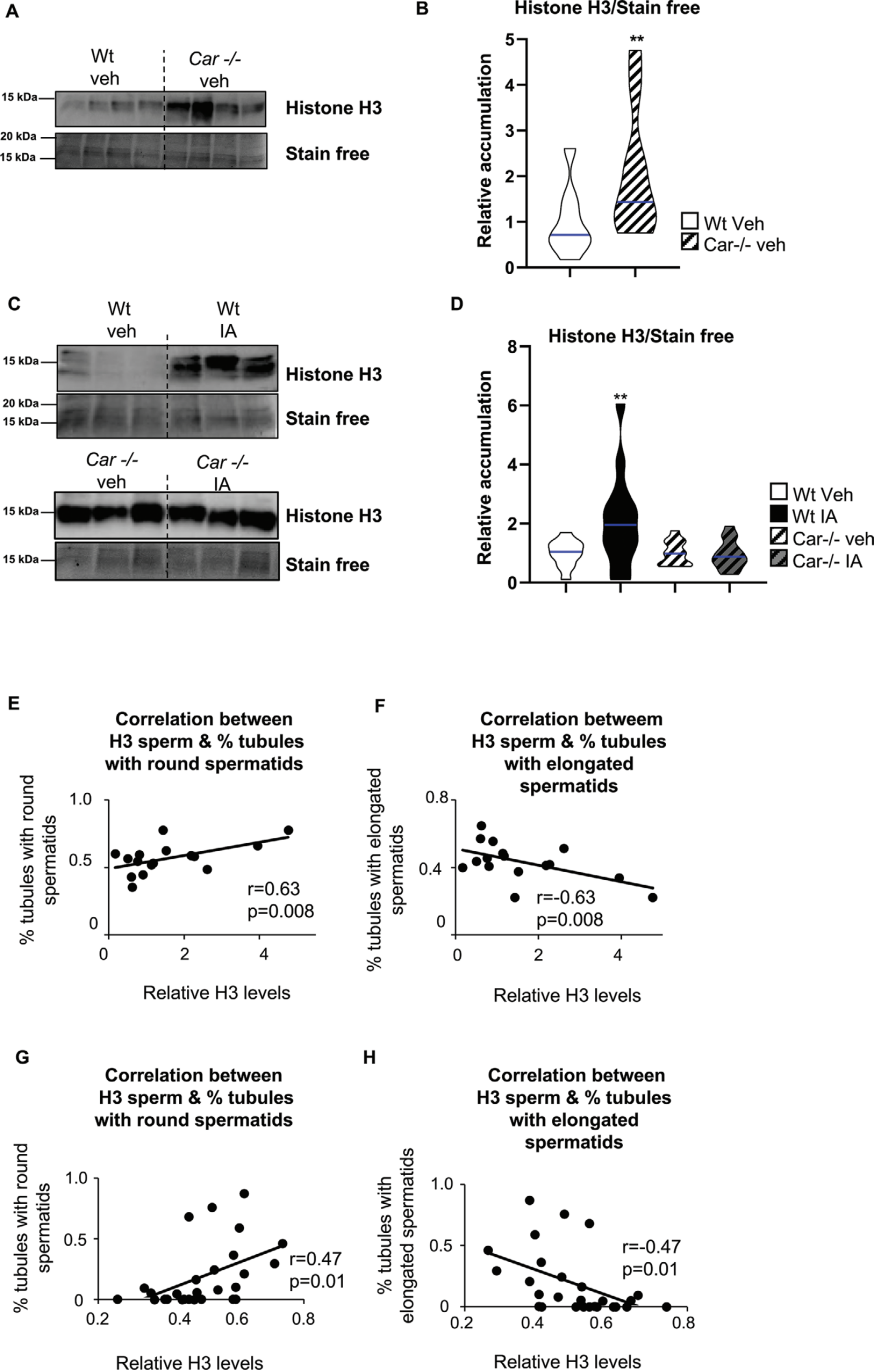
Car inhibition leads to histone retention in sperm cells. A) Representative western blots of H3 in sperm cells of Wt and Car^−/−^ mice (8 months of age). B) Quantification of ratios H3/total proteins in sperm cells of Wt and Car^−/−^ mice. Normalization was performed against total protein using stain‐free gels. The blue line indicates the median for each group. Wt vehicle‐treated group was arbitrarily set at 1. C) Representative western blots of H3 in sperm cells of Wt and Car^−/−^ mice neonatally treated with vehicle or IA. D) Quantification of ratios H3/total proteins in sperm cells of Wt and Car^−/−^ mice neonatally treated with vehicle or IA. Normalization was performed against total protein using stain‐free gels. The blue line indicates the median for each group. Vehicle groups of each genotype were arbitrarily set at 1. E) Correlation between the relative level of H3 in sperm cells and the relative percentage of seminiferous tubules with round elongated stained for Ac‐H4 of Wt and Car^−/−^ mice. F) Correlation between the relative level of H3 in sperm cells and the relative percentage of seminiferous tubules with elongated stained for acH4 of Wt and Car^−/−^ mice. G) Correlation between the relative level of H3 and the relative percentage of seminiferous tubules with round elongated stained for Ac‐H4 in sperm cells of Wt and Car^−/−^ mice neonatally treated with vehicle or IA. H) Correlation between the relative level of H3 and the relative percentage of seminiferous tubules with elongated stained for Ac‐H4 in sperm cells of Wt and Car^−/−^ mice neonatally treated with vehicle or IA. In all panels, Veh: vehicle and IA: Inverse agonist. n = 26 for Wt veh; n = 24 for IA, n = 15 for Car^−/−^ veh and n = 15 for Car^−/−^ IA. T‐test (panel B) was performed to compare two groups and Two‐way ANOVA followed by Holm‐Sidak's test (panel D) was performed for multiple comparisons. A to D **p < 0.01 versus Wt vehicle‐treated male group. E to H, Pearson test was performed.

Thus, we next analyzed histone retention in the sperm cells of IA‐treated Wt males and *Car* ^−^ *^/^* ^−^ vehicle‐treated males compared to Wt vehicle‐treated males (**Figure** **10**). The data showed a higher level of histone H3 in the sperm cells of *Car* ^−^ *^/^* ^−^ males than in Wt males (Figure 10A,B). Similarly, histone H3 retention was observed in the sperm cells of IA‐treated Wt males compared to Wt control males, while IA treatment had no effect in *Car* ^−^ *^/^* ^−^ males compared to vehicle‐treated *Car* ^−^ *^/^* ^−^ males (Figure 10C,D). The results showed a positive correlation between the percentage of seminiferous tubules with round spermatids and the levels of H3 in sperm cells when comparing Wt and *Car* ^−^ *^/^* ^−^ males (Figure 10E,F) and IA‐treated versus vehicle‐treated Wt males (Figure 10G,H). These results suggest that alterations in sperm cells may originate from abnormal testicular spermatogenesis.

### 2.6 Car Inhibition Altered Mature Sperm Production and Head Morphology

**Figure 11:**
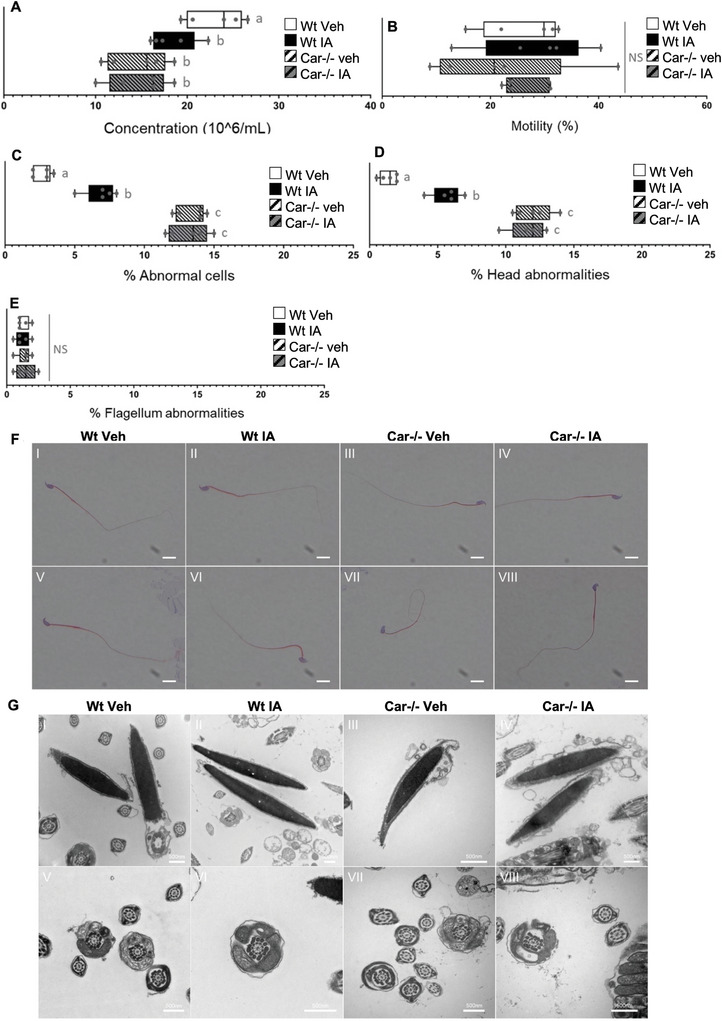
Complete sperm parameters analysis of each condition. A) Relative number of sperm count in the epididymis tail of Wt or Car^−/−^ males (8 months of age). IA‐treated Wt and Car^−/−^ males present lower sperm concentrations than wild‐type individuals. B) Sperm motility was found similar among the conditions. C) Car^−/−^ individuals present more morphology abnormalities than wild‐type individuals. Inverse antagonist treatment induces a slight increase in abnormalities rates of wild‐type but not in Car^−/−^ animals. D) These abnormalities are focused on the head, while in E) no abnormality of the flagella is observed among the different conditions. In all panels, n = 5 per group. Panels A‐E data are presented as horizontal box and whiskers plots and individual data points simultaneously. Statistical significance was assessed using an unpaired Student t‐test. Each group was compared individually with all other groups one by one. For each histogram, plots sharing different small letters represent statistically significant differences between the groups (p < 0.05), and plots with a common letter do not present statistically significant differences between the groups (p > 0.05). The corresponding statistical data can be found in Tables S1 and S2 (Supporting Information). F) Optical microscopy analysis showing for each condition sperm with typical (I‐IV) normal and (V‐VIII) abnormal morphology. E) Transmission electron microscopy analysis confirmed the morphology analysis with most of the sperm cells displaying normal (I‐IV) head and (V‐VIII) flagellum morphology.

IA treatment or *Car* gene deficiency did not affect sperm production, as indicated by the number of sperm cells in the epididymis head (Figure S12A, Supporting Information). In contrast, a slight reduction in sperm count was observed within the epididymal tails of *Car* ^−^ *^/^* ^−^ mice compared to that in Wt mice (**Figure** **11**A). IA treatment had a similar adverse effect on sperm count in Wt mice but not in *Car* ^−^ *^/^* ^−^ mice (Figure 11A). This suggests that altered spermatozoa might be eliminated during epididymal transit; consistent with the histone retention observed, we hypothesized that, some spermatozoa produced by the testis after Car inhibition were abnormal.

To elucidate the impact of Car inhibition on spermatogenesis, a thorough analysis of mature sperm was conducted. No differences were observed among the groups regarding overall sperm motility (Figure 11B) or swimming pattern (Figure S13A–C, Supporting Information). *Car* ^−^ *^/^* ^−^mice exhibited a higher proportion of abnormal sperm cells than Wt mice. IA treatment had a moderately detrimental effect on sperm morphology in Wt mice (Figure 11C). Importantly, these abnormalities were exclusively localized in the sperm head, while the flagella remained structurally intact across all groups (Figure 11D,E). These findings were consistent at both the macroscopic (Figure 11F) and microscopic levels (Figure 11G).

**Figure 12:**
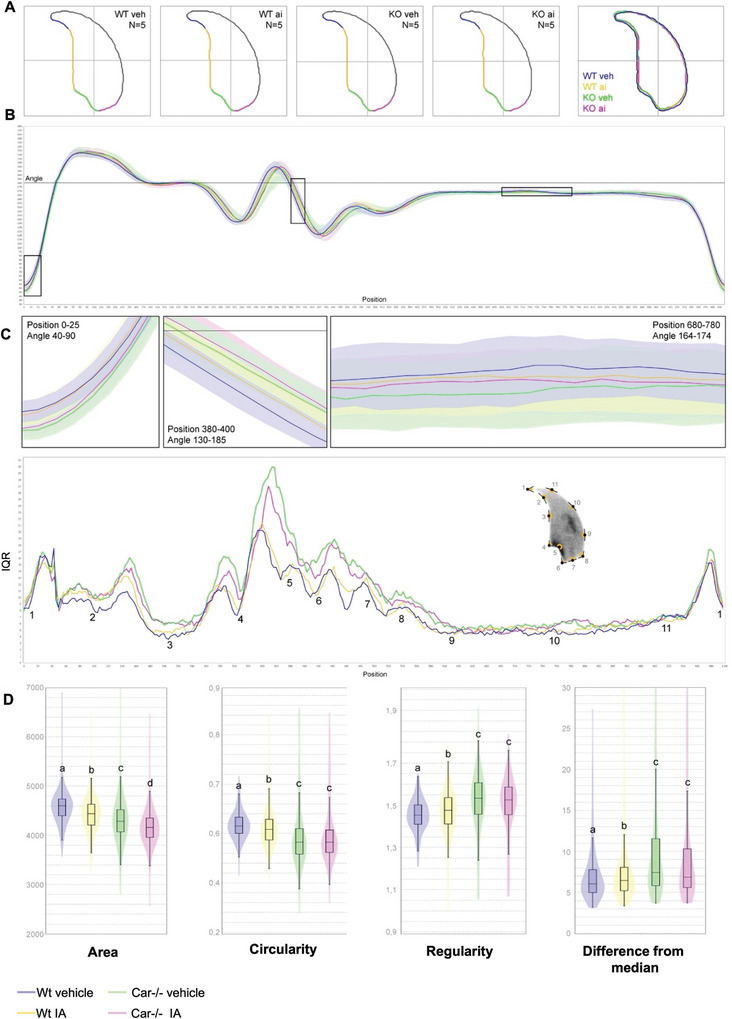
Fine nuclear morphology analysis of sperm cells from each condition. A) Consensus nuclear outlines for each condition and merged consensus nucleus (blue = W Wt veh, yellow = Wt IA, green = Car^−/−^ veh, and pink = Car^−/−^ IA). B) Angle profiles for each condition with focuses (black boxes) on specific positions. The abscissa is an index of the percentage of the total perimeter measured counterclockwise from the apex of the sperm hook. Ordinate represents the interior angle measured across a sliding window centered on each index location. C) Variability profiles of sperm cells from each condition. Abscissa is similar to the angle profile and the ordinate represents the InterQuartile Range (IQR, defined by the difference between the 75th and the 25th percentiles). Some regions of the nuclei are mapped on the profile and the graphical representation (from Skinner et al. 2019^[^ ^55^ ^]^) to ease reading. D) Violins plots of nuclear parameters. Statistical significance of differences between populations was automatically assessed using the software (Mann–Whitney U test, significantly different populations are identified by distinct letters). In all panels, n = 5 per group.

To further characterize the abnormalities in the sperm head, we used Nuclear Morphology Analysis Software^[^ ^55^ ^]^ to evaluate the nuclear morphologies of sperm from each group. Shape modeling revealed a largely congruent consensus and angle profile for the nuclei, underscoring the shared genetic background among the mice (**Figure** **12**A,B). A slight but significant difference was observed between IA‐ and vehicle‐treated Wt mice. However, *Car* ^−/−^ mice exhibited greater variability in nuclear morphology than Wt mice (Figure 12C), affecting fundamental nuclear morphology parameters (Figure 12D).

### 2.7 Car Activity Inhibition Through Genetic or Pharmacological Approaches Led to Male Fertility Disorders

**Figure 13:**
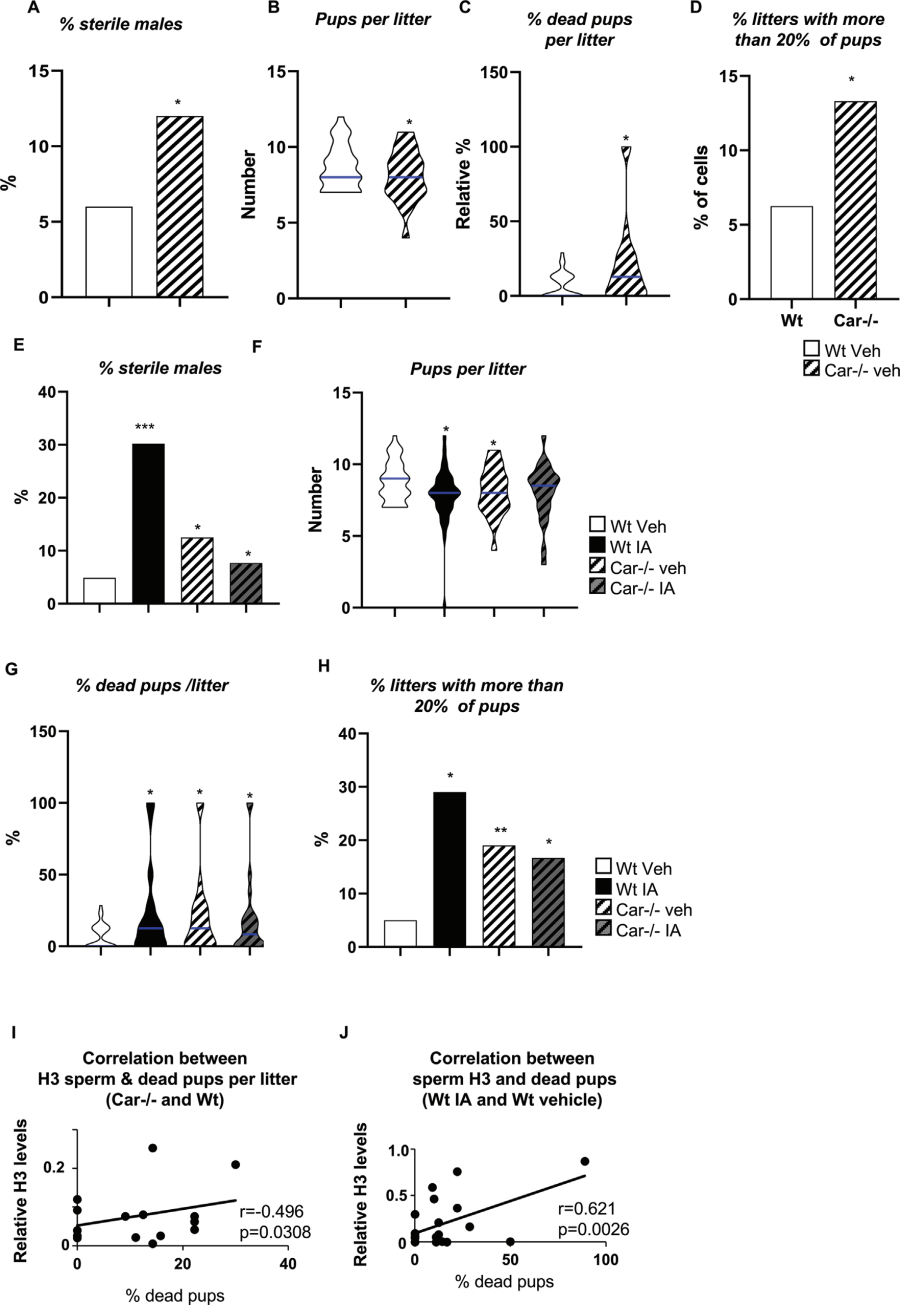
Analysis of fertility in 8‐month‐old adult males. A) Percentage of sterile Wt or Car^−/−^ males. B) Number of pups per litter obtained from fertile Wt or Car^−/−^ males. The blue line indicates the median of each group. C) Percentage of dead pups per litter obtained from fertile Wt or Car^−/−^ males. The blue line indicates the median of each group. D) Percentage of litters with more than 20% of dead pups obtained from fertile Wt or Car^−/−^ males. E) Percentage of sterile Wt or Car^−/−^ males treated neonatally with the vehicle of IA. F) Number of pups per litter obtained from fertile Wt or Car^−/−^ males treated neonatally with the vehicle of IA. The blue line indicates the median of each group. G) Percentage of dead pups per litter obtained from fertile Wt or Car^−/−^ males treated neonatally with the vehicle of IA. The blue line indicates the median of each group. The blue line indicates the median of each group. H) Percentage of litters with more than 20% of dead pups obtained from fertile Wt or Car^−/−^ males treated neonatally with the vehicle of IA. I) Correlation between the relative level of H3 in sperm cells and the relative percentage of dead pups per litter obtained from Wt and Car^−/−^ mice. J) Correlation between the relative level of H3 in sperm cells and the relative percentage of dead pups per litter obtained from Wt and Car^−/−^ mice neonatally treated with vehicle or IA. In A to D: The number of males in breeding was n = 39 for Wt and 24 for Car^−/−^ mice. In D to H: The number of males Vehicle‐treated Wt n = 39 and IA‐treated Wt n = 43; Vehicle Car^−/−^ n = 24 and n = 25 IA‐treated Car^−/−^; in K): n = 39 for Wt, 24 for Car^−/−^ mice. In L): Wt n = 39 and IA‐treated Wt n = 43; Vehicle‐treated Car^−/−^ n = 24 and IA‐treated Car^−/−^ n = 25. In M): n = 26 for Wt veh; n = 24 for IA; n = 15 for Car^−/−^ veh; and n = 15 for Car^−/−^ IA. In all panels, Veh: vehicle and IA: Inverse agonist. In A, D, E, and H Xi2 analysis were performed. In B and C, T‐test was performed. In F and G, two‐way ANOVA followed by Holm–Sidak's test was performed for multiple comparisons. *p < 0.05 and ***p < 0.001 Wt vehicle‐treated male group. For correlation, the Pearson's test was performed.

To determine the impact of inhibiting Car signaling on male fertility and the relative importance of reduced sperm count or impaired sperm quality, we performed reproductive tests on 8‐month‐old Wt or *Car* ^−^ *^/^* ^−^ male mice exposed to vehicle or IA during the neonatal period (1–10 dpn). No difference in the percentage of plugged females was found between groups (Figure S14A, Supporting Information). Fertility was assessed by studying the number of embryos at E.14.5 post‐coitum. A statistically significant difference in the percentage of sterile males (defined by the absence of pups or all progenies being dead in the litters) was found between Wt and *Car* ^−^ *^/^* ^−^ males. Specifically, 5% (2/39) of Wt males and 12.5% (3/24) of *Car* ^−^ *^/^* ^−^ males were sterile (**Figure** **13**A).

To determine whether the sterility was due to non‐fecundity or embryo loss during development, the number of embryos was counted at E2.5 post‐coitum. No difference was observed in the number of embryos per litter at E2.5, regardless of genotype or treatment (Figure S14B, Supporting Information). However, at E14.5, *Car* ^−^ *^/^* ^−^ males had a statistically significant reduction in the number of offspring per litter, suggesting embryo loss and resorption between E2.5 and E14.5 (Figure 13B). Consistent with this, *Car* gene knockout was associated with a higher rate of dead pups per litter than Wt males at E.14.5 (Figure 13C). Additionally, *Car* ^−^ *^/^* ^−^ mice had a higher litter rate with more than 20% dead pups than Wt mice (Figure 13D). These data suggest that *Car* gene deficiency negatively impacts male fertility.

The impact of neonatal IA inhibition of Car activity on male fertility was also analyzed 8 months after treatment. No difference was found between the groups regarding the percentage of plugged females producing progeny (Figure S14A, Supporting Information). Fertility was assessed by studying the number of embryos at E.14.5 post‐coitum. Specifically, 30% (13/43) of IA‐treated Wt males were sterile (defined by the absence of pups or all progenies being dead in the litters), compared to only 5% (2/39) of vehicle‐treated Wt males (Figure 13E). IA treatment had no effect on *Car* ^−^ *^/^* ^−^ males, with 8.0% (2/25) of IA‐treated *Car* ^−^ *^/^* ^−^ mice being sterile compared to 12.5% (3/24) of vehicle treated Car^−/−^ male mice (Figure 13E).

No difference was observed in the number of embryos per litter at E2.5 days post‐coitum, regardless of the genotype or treatment (Figure S14B, Supporting Information). However, IA‐treated Wt males that remained fertile generated fewer pups per litter at E14.5 than the vehicle treated Wt males, suggesting that some embryos may have died during the early developmental stages (Figure 13F). Moreover, a higher death rate of dead pups was observed in litters originating from IA‐treated Wt males than those from control male mice (Figure 13G). Notably, IA treatment showed no effect in *Car* ^−^ *^/^* ^−^ males (Figure 13G). Additionally, a higher rate of litters with more than 20% dead pups was observed in IA‐treated Wt than in vehicle‐treated Wt male mice (Figure 13H).

In this study, we observed a similar number of embryos per litter at E2.5 days post‐coitum across all genotypes or treatments. However, at E.14.5, a higher rate of dead pups per litter was observed in *Car* ^−/−^ males and IA‐treated Wt males than in the vehicle treated Wt males, suggesting that the impact of Car inhibition (genetic or pharmacological) on male fertility might be associated with an alteration in the quality rather than the number of germ cells. This hypothesis was supported by correlation analyses, which showed a positive correlation between histone H3 levels in spermatozoa and the number and percentage of dead pups per litter when comparing Wt and *Car* ^−^ *^/^* ^−^ males (Figure 13I) and IA‐treated versus vehicle‐treated Wt males (Figure 13J). Overall, our findings demonstrate that *Car* gene deficiency or pharmacological inhibition of its activity led to fertility defects in males with similar phenotypes, including altered neonatal spermatogonia homeostasis, altered spermatid homeostasis associated with an increased percentage of sterile males, and an increased incidence of dead progenies.

### 2.8 Car Inhibition Modulated the Pi3k‐Akt‐Foxo1 Signaling Pathway in Spermatogonia

Undifferentiated germ cells are responsible for the multiple waves of spermatogenesis throughout life. Therefore, the observed impacts of Car neonatal inhibition on male fertility could stem from alterations in spermatogonia, the primary germ cell population present during this critical exposure period. To delineate the molecular mechanism underlying the effects of the Car signaling pathway on germ cell homeostasis, we performed in vitro experiments using the spermatogonial C18‐4 cell line.^[^ ^56^ ^]^ Using CRISPR/Cas9 technology, we generated a C18‐4 cell line deficient in the *Car* gene (Car‐KO cells) (see Experimental Section and Figure S15A–D, Supporting Information). qPCR revealed that *Car* was not expressed in the knockout cells (Figure S15E, Supporting Information).

**Figure 14:**
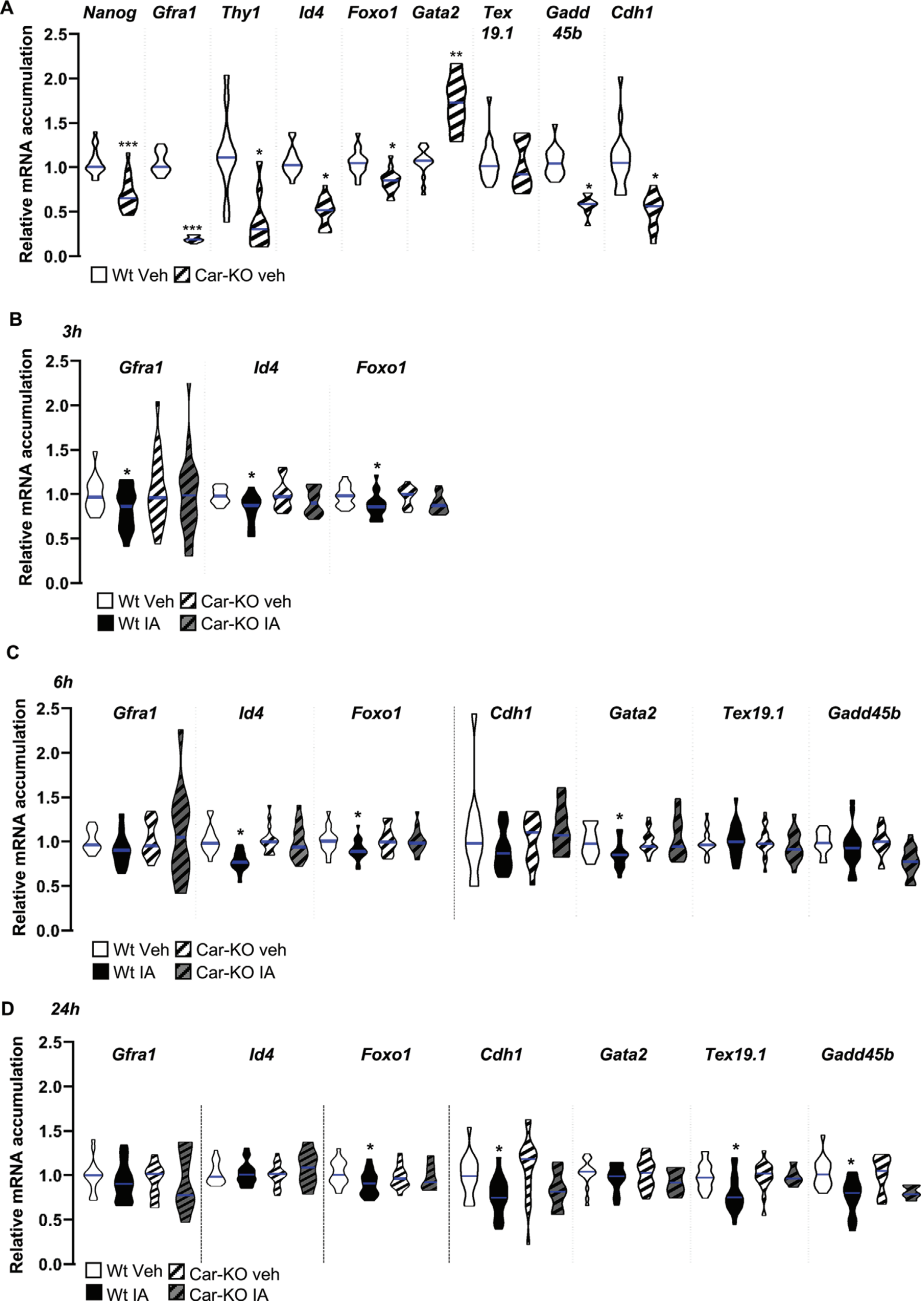
A) Relative mRNA accumulations of *Nanog, Gfra1, Thy1, Id4, Foxo1, Gata2, Tex19.1, Gadd45b*, and *Cdh1* normalized to β‐actin on Wt and CAR‐KO cells. The blue line indicates the median for each group. The Wt group was arbitrarily set at 1.B) Relative mRNA accumulations of *Gfra1, Id4*, *and Foxo1* normalized to β‐actin on Wt and CAR‐KO cells treated with vehicle or IA. Analyses were performed at 3 h after treatment. The blue line indicates the median for each group. Vehicle‐treated groups of each genotype were arbitrarily set at 1. C) Relative mRNA accumulations of *Gfra1, Id4, Foxo1, Cdh1*, *Gata2, Tex19.1*, and *Gadd45b* normalized to β‐actin on Wt and CAR‐KO cells treated with vehicle or IA. Analyses were performed at 6 h after treatment. The blue line indicates the median for each group. Vehicle‐treated groups of each genotype were arbitrarily set at 1. D) Relative mRNA accumulations of *Gfra1, Id4, Foxo1, Cdh1*, *Gata2, Tex19.1*, and *Gadd45b* normalized to β‐actin on Wt and CAR‐KO cells treated with vehicle or IA. Analyses were performed 24 h after treatment. The blue line indicates the median for each group. Vehicle‐treated groups of each genotype were arbitrarily set at 1. In panel A, T‐tests were performed for comparison of two groups and in panels B to D, two‐way ANOVA followed by Holm–Sidak's test was performed for multiple comparisons. *p < 0.05, and ***p < 0.001, versus vehicle‐treated Wt males.

We then elucidated the impact of *Car* deficiency on this cell line compared to Wt cells, as well as the effect of IA treatment in both Wt and Car‐KO cells at various time points. Compared to Wt cells, Car knockout led to a lower mRNA expression of genes associated with undifferentiated germ cells, such as *Nanog* (Nanog homeobox), *Gfra1* (GDNF family receptor alpha 1), *Thy1* (Thy‐1 cell surface antigen), and *Id4* (**Figure** **14**A). Additionally, IA treatment also led to lower mRNA expression of *Gfra1* in Wt cells at 3 h after treatment. In addition, a decrease in *Id4* mRNA expression was observed at 3 and 6 h after treatment with IA in Wt cells (Figure 14B,C). Notably, IA treatment did not affect Car‐KO cells (Figure 14B,C), suggesting that the Car signaling pathway altered key marker expression of undifferentiated germ cells.

**Figure 15:**
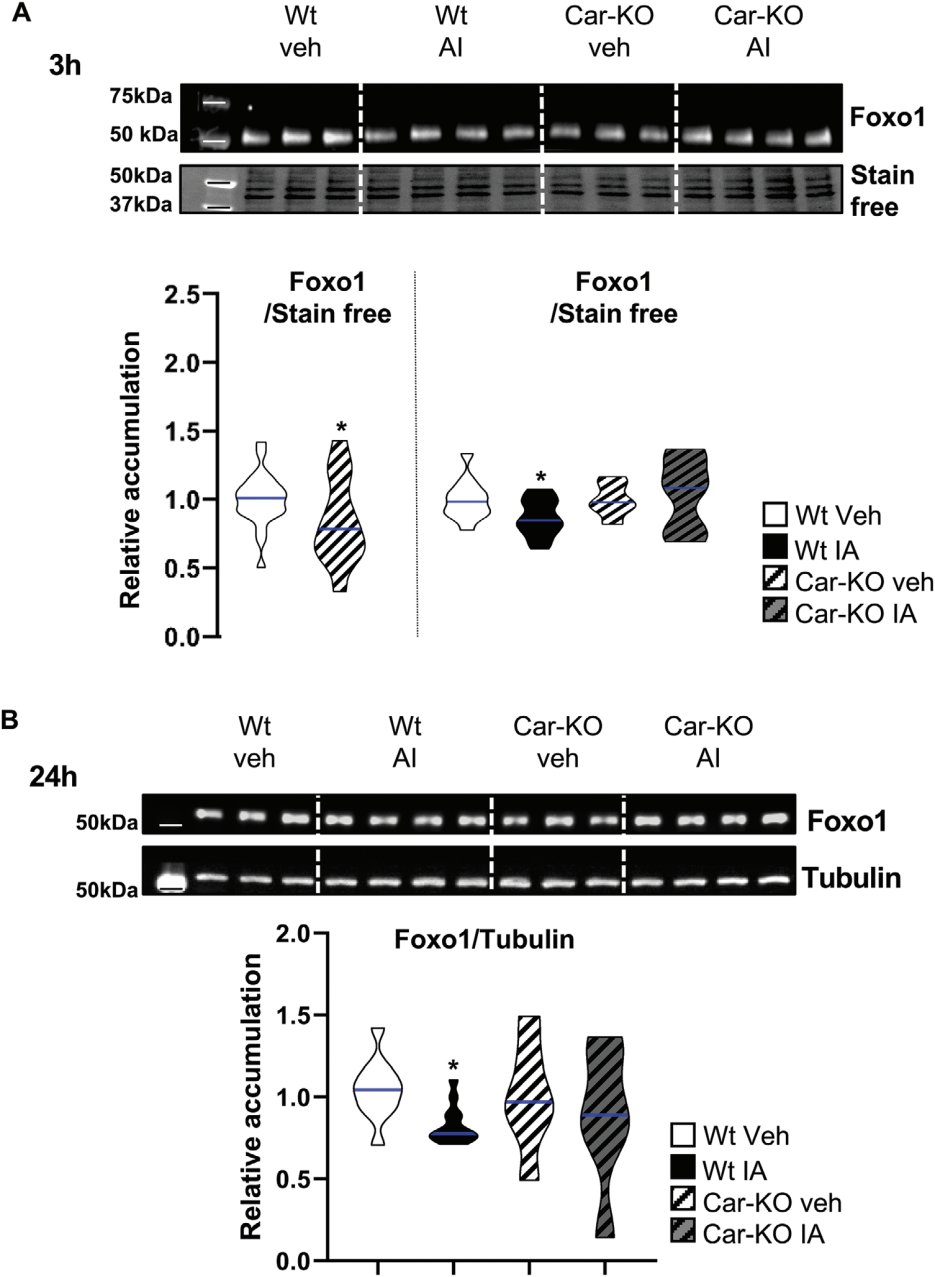
A) (Top panel) Representative western blots of Foxo1 in Wt and Car‐KO cells treated with vehicle or IA for 3 h. (Bottom panel) Quantification of ratios in Wt and Car‐KO cells. Normalization was performed against total protein using stain‐free gels. The blue line indicates the median for each group. Vehicle‐treated Wt cells were arbitrarily set at 1. Quantification of ratios in Wt and Car‐KO cells treated with vehicle or IA for 3 h. Normalization was performed against total protein using stain‐free gels. The blue line indicates the median for each group. Vehicle‐treated cellsof each genotype were arbitrarily set at 1. B) (Top panel) Representative western blots of Foxo1 Wt and Car‐KO cells treated with vehicle or IA for 24 h. (Bottom panel) Quantification of ratios in Wt and Car‐KO cells treated with vehicle or IA for 24 h. Normalization was performed against total protein using stain‐free gels. The blue line indicates the median for each group. Vehicle groups of each genotype were arbitrarily set at 1. In all panels, Veh: vehicle and IA: Inverse agonist. In panels A, n = 27 per group; in panel B n = 15 per group from five independent experiments. T‐test was performed for comparison of two groups and two‐way ANOVA followed by Holm–Sidak's test was performed for multiple comparisons. *p < 0.05, and ***p < 0.001, versus vehicle‐treated Wt males.

Consistent with the in vivo immunohistochemistry data (Figure 3), a decrease in *Foxo1* mRNA accumulation was observed from 3 to 24 h after IA treatment in Wt cells (Figure 14B–D). IA treatment did not affect Car‐KO cells (Figure 14B); however, a decrease in *Foxo1* mRNA expression was observed in vehicle‐treated Car‐KO cells compared to that in Wt cells (Figure 14A). Notably, the effects of the Car signaling pathway on *Foxo1* mRNA expression were validated at the protein level, showing decreased Foxo1 accumulation in vehicle‐treated Car‐KO cells compared to Wt cells (**Figure** **15**A,B), as well as in IA‐treated Wt cells at 3 to 24 h compared to vehicle‐treated Wt cells (Figure 15A,B). The impact on the Foxo1 signaling pathway was confirmed through the reduced mRNA accumulation of known target genes of Foxo1, such as Gata binding factor 2 (*Gata2)*,^[^ ^20^ ^]^ Testis‐expressed protein 19 (*Tex19.1)*,^[^ ^20^ ^]^ Growth arrest and DNA damage‐inducible protein 45 (*Gadd45)*,^[^ ^57^ ^]^ and Cadherin 1 (*Cdh1)*,^[^ ^58^ ^]^ specifically in Wt cells treated with IA for either 6 or 24 h (Figure 14C,D). These results highlight that during IA treatment, the changes in *Foxo1* mRNA accumulation preceded the impact on its target genes, indicating that IA treatment alters the transcriptional activity of Foxo1. Similarly, a lower mRNA accumulation of *Gadd45b* and *Cdh1* was detected in vehicle‐treated Car‐KO cells than in Wt cells (Figure 14A).

Although the exact mechanisms regulating mRNA for each gene were not defined, protein level variations for Gfra1 and Gata2 were validated compared to vehicle‐treated cells. Protein accumulation was affected in Car‐KO cells compared to that in Wt cells and IA‐treated Wt cells compared to vehicle‐treated Wt cells. These changes corresponded with the observed modulation of mRNA levels (Figure S16, Supporting Information).

**Figure 16:**
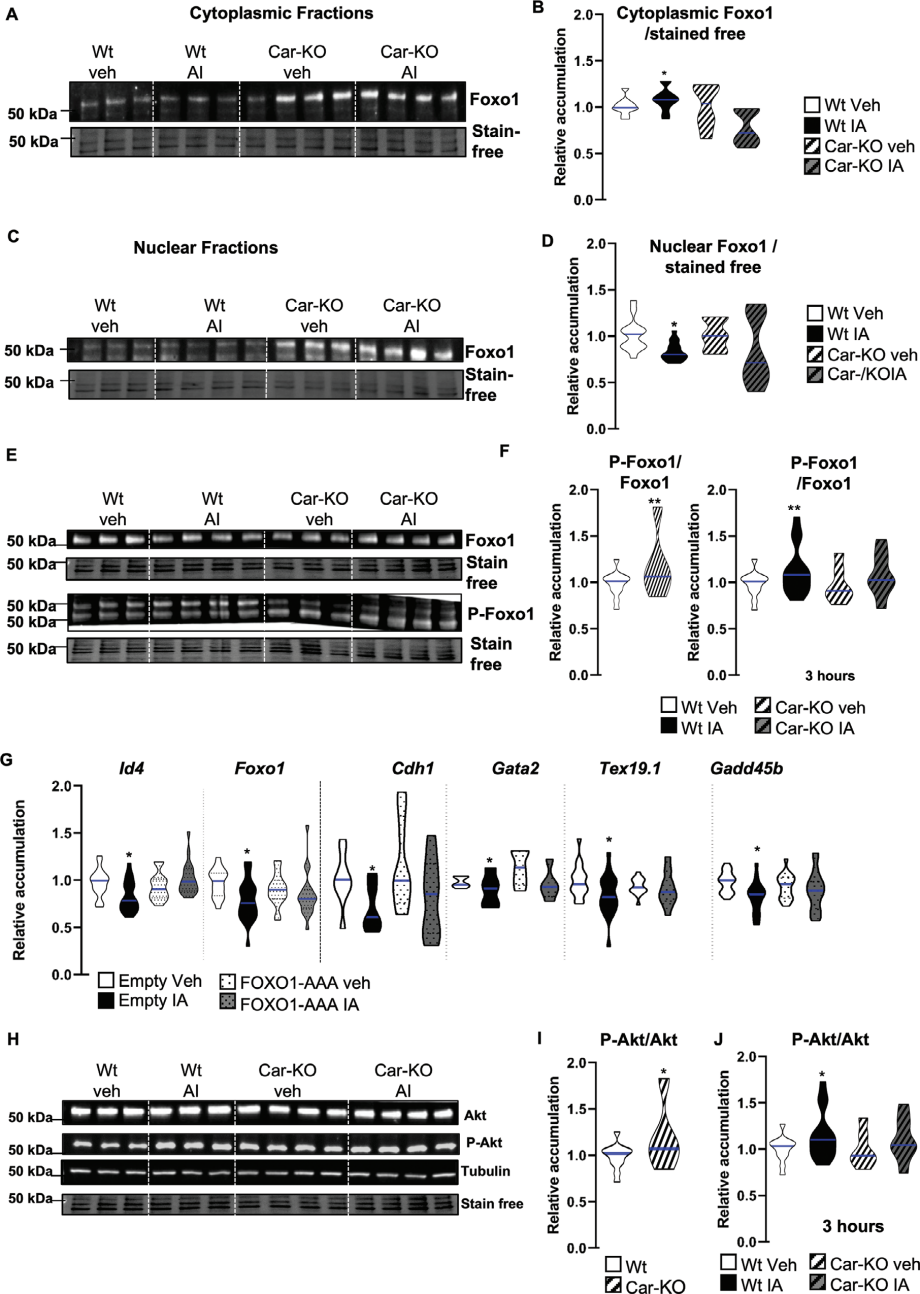
A) Representative western blots of Foxo1 on cytoplasmic compartments of Wt and Car‐KO cells treated with vehicle or IA for 3 h. B) Quantification of ratios of Foxo1/total proteins in cytoplasmic compartments of Wt and Car‐KO cells treated with vehicle or IA for 3 h. Normalization was performed against total protein using stain‐free gels. The blue line indicates the median for each group. Vehicle groups of each genotype were arbitrarily set at 1. C) Representative western blots of Foxo1 on the nuclear compartment of Wt and Car‐KO cells treated with vehicle or IA for 3 h. D) Quantification of ratios Foxo1/total proteins in nuclear compartments of Wt and Car‐KO cells treated with vehicle or IA for 3 h. Normalization was performed against total protein using stain‐free gels. The blue line indicates the median for each group. Vehicle groups of each genotype were arbitrarily set at 1. E) Representative western blots of Foxo1 and Phospho‐Foxo1 in Wt and Car‐KO cells treated with vehicle or IA for 3 h. F) (Left panel) Quantification of ratios P‐Foxo1/Foxo1 in Wt and Car‐KO cells in which normalization was performed against total protein using stain‐free gels and Wt vehicle cells were arbitrarily set at 1; and (Right panel) quantification of ratios P‐Foxo1/Foxo1 in Wt and Car‐KO cells treated with vehicle or IA for 3 h for which normalization was performed against total protein using stain‐free gels. The blue line indicates the median for each group. Vehicle groups of each genotype were arbitrarily set at 1. G) Relative mRNA accumulations of *Id4, Foxo1, Cdh1, Gata2, Tex19.1, and Gadd45b* normalized to β‐actin on Wt cells transfected with empty vector or a vector for overexpression of non‐phosphorylable Foxo1 (FOXO1‐AAA) treated with vehicle or IA for 24 h. The blue line indicates the median for each group. Vehicle‐treated groups of each condition of transfection (empty vector or pFOXO1‐AAA) were arbitrarily set at 1. H) Representative western blots of Akt and Phospho‐Akt in Wt and Car‐KO cells treated with vehicle or IA for 3 h. I) Quantification of ratios of P‐Akt/Akt in Wt and Car‐KO cells in which normalization was performed against total protein using stain‐free gels. The blue line indicates the median for each group. Wt vehicle cellsof each genotype were arbitrarily set at 1.J) Quantification of ratios P‐Akt/Akt in Wt and Car‐KO cells treated with vehicle or IA for 3 h and normalization was performed against total protein using stain‐free gels. The blue line indicates the median for each group. Vehicle groups of each genotype were arbitrarily set at 1.In all panels, Veh: vehicle and IA: Inverse agonist. In panels A to F and H, n = 25, and in panel G, n = 15 per group. T‐test was performed for comparison of two groups (panels F and I) Two‐way ANOVA followed by Holm–Sidak's test for multiple comparisons. *p < 0.05 and **p < 0.01 versus the respective vehicle group for each genotype or versus the respective vehicle group for the condition of transfection.

Consistent with the in vivo findings, the cellular localization of Foxo1 was examined to further understand the impact of Car signaling on the Foxo1 signaling pathway. For this purpose, its repartitioning in the cellular compartment following IA exposure was evaluated using western blotting of samples from subcellular fractions (**Figure** **16**). The efficiency of the fraction methods was validated using western blots with Gapdh as a marker of the cytoplasmic compartment and histone H3 as a marker of the nucleus/chromatin (Figure S17A, Supporting Information). IA treatment modulates the ratio of nuclear to cytoplasmic Foxo1. Consistent with the in vivo observations (Figures 6, 7, and 8), IA treatment led to lower Foxo1 levels in the nucleus and higher levels in the cytoplasm (Figure 16A–D). These findings suggest that IA acts in a Car‐dependent manner, as revealed using Car‐KO cells (Figure 16A–D).

The cellular trafficking of Foxo1 is regulated by its phosphorylation.^[^ ^59^ ^]^ Our data showed that IA treatment increased the level of phosphorylated Foxo1 in Wt cells compared to vehicle‐treated controls, correlating with reduced nuclear accumulation of Foxo1. In contrast, IA had no effect on Car‐KO cells (Figure 16E,F). Consistently, Car knockout led to a higher P‐Foxo1/Foxo1 ratio than Wt cells under vehicle‐treated conditions (Figure 16E,F). To determine whether the phosphorylation of Foxo1 and its retention in the cytoplasm were involved in these observed effects, we performed experiments using constitutively active Foxo1 retained in the nucleus, where the phosphorylation sites were mutated (Foxo1‐AAA).^[^ ^59^ ^]^ qPCR analysis demonstrated that if IA treatment affected the mRNA accumulation of *Id4*, *Foxo1*, *Cdh1*, *Tex19.1*, and *Gadd45b* in cells transfected with an empty vector, no effect was observed in cells transfected with a plasmid overexpressing Foxo1‐AAA (Figure 16G). These findings support a major crosstalk between Car and Foxo1 signaling, resulting in the observed phenotypes.

To determine how the Car signaling pathway interacts with Foxo1 phosphorylation in spermatogonia, we focused on the Pi3k pathway, as it has been demonstrated that Akt phosphorylates Foxo1^[^ ^60^ ^]^ and that Car can modulate the Akt pathway.^[^ ^61^ ^]^ The results showed that IA treatment increased the P‐Akt/Akt ratio, as detected 3 h after treatment (Figure 16H,I), which correlated with elevated P‐Foxo1 levels. Similarly, *Car* knockout exhibited effects akin to Car pharmacological inhibition, showing a higher P‐Akt/Akt ratio in Car‐KO cells than in Wt cells (Figure 16H,I).

Previous reports have demonstrated that the Car signaling pathway may influence the Pi3k‐Akt pathway by affecting Pten protein accumulation.^[^ ^62^, ^63^ ^]^ Notably, our current data showed that Car may regulate Pten at protein levels in germ cells, potentially explaining the crosstalk between CAR and Akt signaling (Figure S17B,C, Supporting Information).

**Figure 17:**
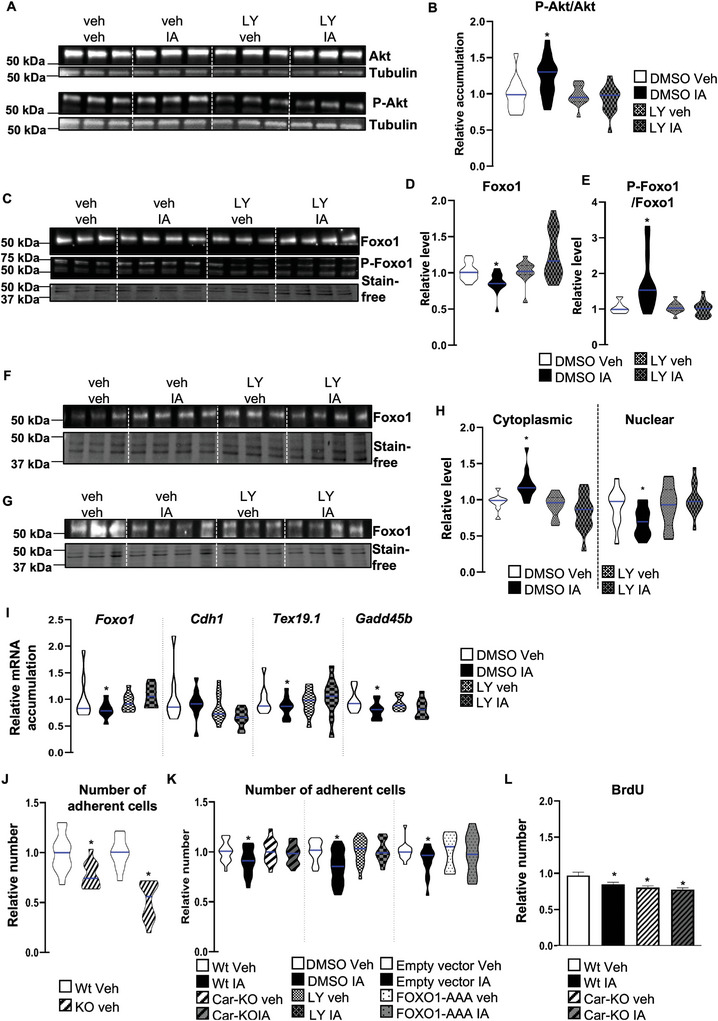
A) Representative western blots of Akt and Phospho‐Akt in Wt and Car‐KO cells treated with vehicle or LY (LY294002) for 45 min and then with vehicle or IA for 3 h. B) Quantification of ratios P‐Akt/Akt in Wt and Car‐KO cells treated with vehicle or LY (LY294002) for 45 min and then with vehicle or IA for 3 h. Normalization was performed against total protein using stain‐free gels. The blue line indicates the median for each group. Vehicle groups of each condition (DMSO or LY) were arbitrarily set at 1. C) Representative western blots of Foxo1 and Phospho‐Foxo1 in Wt and Car‐KO cells treated with vehicle or LY (LY294002) for 45 min and then with vehicle or IA for 3 h. D) Quantification of ratios Foxo1/total proteins in Wt and Car‐KO cells treated with vehicle or LY (LY294002) for 45 min and then with vehicle or IA for 3 h. The blue line indicates the median for each group. E) Quantification of ratios of P‐Foxo1/Foxo1 in Wt and Car‐KO cells treated with vehicle or LY (LY294002) for 45 min and then with vehicle or IA for 3 h. Normalization was performed against total protein using stain‐free gels. The blue line indicates the median for each group. Vehicle groups of each condition (DMSO or LY) were arbitrarily set at 1. F) Representative western blots of Foxo1 on cytoplasmic compartments of Wt and Car‐KO cells treated with vehicle or LY (LY294002) for 45 min and then with vehicle or IA for 3 h. G) Representative western blots of Foxo1 on the nuclear compartment of Wt and Car‐KO cells treated with vehicle or LY (LY294002) for 45 min then with vehicle or IA for 3 h. H) (Left panel) Quantification of cytoplasmic ratios of Foxo1/total proteins in Wt and Car‐KO cells treated with vehicle or LY (LY294002) for 45 min and then with vehicle or IA for 3 h. Normalization was performed against total protein using stain‐free gels and vehicle groups of each condition (DMSO or LY) were arbitrarily set at 1. (Right panel) Quantification of nuclear ratios of Foxo1/total proteins in Wt and Car‐KO cells treated with vehicle or LY (LY294002) for 45 min and then with vehicle or IA for 3 h. The blue line indicates the median for each group. Normalization was performed against total protein using stain‐free gels and vehicle groups of each condition (DMSO or LY) were arbitrarily set at 1. I) Relative mRNA accumulations of *Foxo1, Gata2, Tex19.1, Gadd45b*, and *Cdh1* normalized to β‐actin on Wt and Car‐KO cells treated with vehicle or LY (LY294002) for 45 min and then with vehicle or IA for 3 h. The blue line indicates the median for each group. Vehicle groups of each condition (DMSO or LY) were arbitrarily set at 1. J) Relative number of adherent cells in Wt and Car‐KO cells. The blue line indicates the median for each group. Wt cells were arbitrarily set at 1. K) Relative number of adherent cells in Wt and Car‐KO cells treated with vehicle or IA for 24 h (Left part) for which vehicle groups of each genotype were arbitrarily set at 1; Relative number of adherent cells in Wt and Car‐KO cells treated with vehicle or LY (LY294002) for 45 min and then with vehicle or IA for 24 h (Middle part) for which vehicle groups of each condition (DMSO or LY) were arbitrarily set at 1; and Relative number of adherent cells in Wt cells transfected with empty vector or a vector for overexpression none phosphorylable Foxo1 (Foxo1‐AAA) and then treated with vehicle or IA for 24 h (Right part); vehicle groups of each condition (empty vector of Foxo1‐AAA) was arbitrarily set at 1. The blue line indicates the median for each group. L) Quantification of the relative number of BrdU positive in Wt and Car‐KO cells treated with vehicle or IA for 24 h. Vehicle‐treated cells were arbitrarily set at 1. M) Quantification of the relative number of TUNEL positive in Wt and Car‐KO cells treated with vehicle or IA for 24 h. The blue line indicates the median for each group. Vehicle‐treated cells were arbitrarily set at 1. In all panels Veh: vehicle and IA: Inverse agonist. n = 25 from six independent experiments. Two‐way ANOVA followed by Holm–Sidak's test for multiple comparisons; except for panel J in which T‐test was performed for comparison of two groups. In B to I: *p < 0.05 versus DMSO‐vehicle group. In J and L: *p < 0.05 versus Wt vehicle‐treated male group. The horizontal square brackets underline the groups statistically compared between two conditions of different genotypes. In K, the p < 0.05 is either versus Wt‐veh, DMSO‐veh or versus vehicle cells transfected with empty vector.

To investigate the role of Akt in modulating Foxo1 expression, Pi3k activity was inhibited using LY294002 (**Figure** **17**). The data showed that LY 294002 inhibited the IA‐induced increase in the P‐Akt/Akt ratio (Figure 17A,B, Supporting Information). This inhibition of the Pi3k‐Akt pathway also mitigated IA's effects on P‐Foxo1 and total Foxo1 accumulation, thereby altering the P‐Foxo1/Foxo1 ratio (Figure 17C,D, Supporting Information). Furthermore, pretreatment with LY294002 prevented IA‐induced changes in the subcellular localization of Foxo1 (Figure 17E–H), specifically preventing its cytoplasm accumulation and maintaining nuclear levels (Figure 17E–H). Reduced nuclear Foxo1 levels suggest diminished Foxo1 transcriptional activity. qPCR analyses confirmed that LY294002 pretreatment attenuated IA‐induced alterations in mRNA levels of *Foxo1*, *Cdh1*, *Tex19.1*, and *Gadd45b* (Figure 17I).

These findings underscore the interaction between the Car signaling pathway and the Pi3k‐Akt‐Foxo1 pathway in mediating detrimental effects on spermatogonia. The functional link between the inhibition of Car activity and the Pi3k‐Akt‐Foxo1 signaling cascade was supported by the fact that there were higher P‐Akt/Akt and P‐Foxo1/Foxo1 ratios in IA‐treated Wt cells and vehicle‐treated Car‐KO cells than in vehicle‐treated Wt cells (Figure 16). This was, at least in part, associated with a lower level of Foxo1 in *Car*‐KO cells than in Wt cells (Figures 14A and 15A).

The Pi3k/Akt and Foxo1 pathways regulate cell proliferation. Notably, *Car* deficiency resulted in fewer adherent cells than in Wt cells (Figure 17J). Similarly, treatment with Car IA also reduced the number of adherent Wt cells (Figure 17K), an effect not observed in *Car*‐KO cells (Figure 17K). In *Car*‐KO cells or IA‐treated Wt cells, these effects on cell number might be associated with the impact of IA on cell proliferation (Figure 17L), rather than on apoptosis (Figure S18, Supporting Information).

To investigate whether the Pi3k/Akt‐Foxo1 pathway mediates the effects of the Car signaling pathway on cell proliferation, cells were pretreated with LY294002. This pre‐treatment effectively reversed the IA‐induced reduction in the number of adherent cells (Figure 17K). Similarly, even though the effect of IA treatment on the number of adherent cells was confirmed in cells transfected with the empty vector, no effect was detected in cells transfected with non‐phosphorylated Foxo1 (Foxo1‐AAA) (Figure 17K). These findings demonstrate that the crosstalk between Car and the Pi3k/Akt/Foxo1 signaling pathway is crucial in mediating the impact of Car on spermatogonial cells.

**Figure 18:**
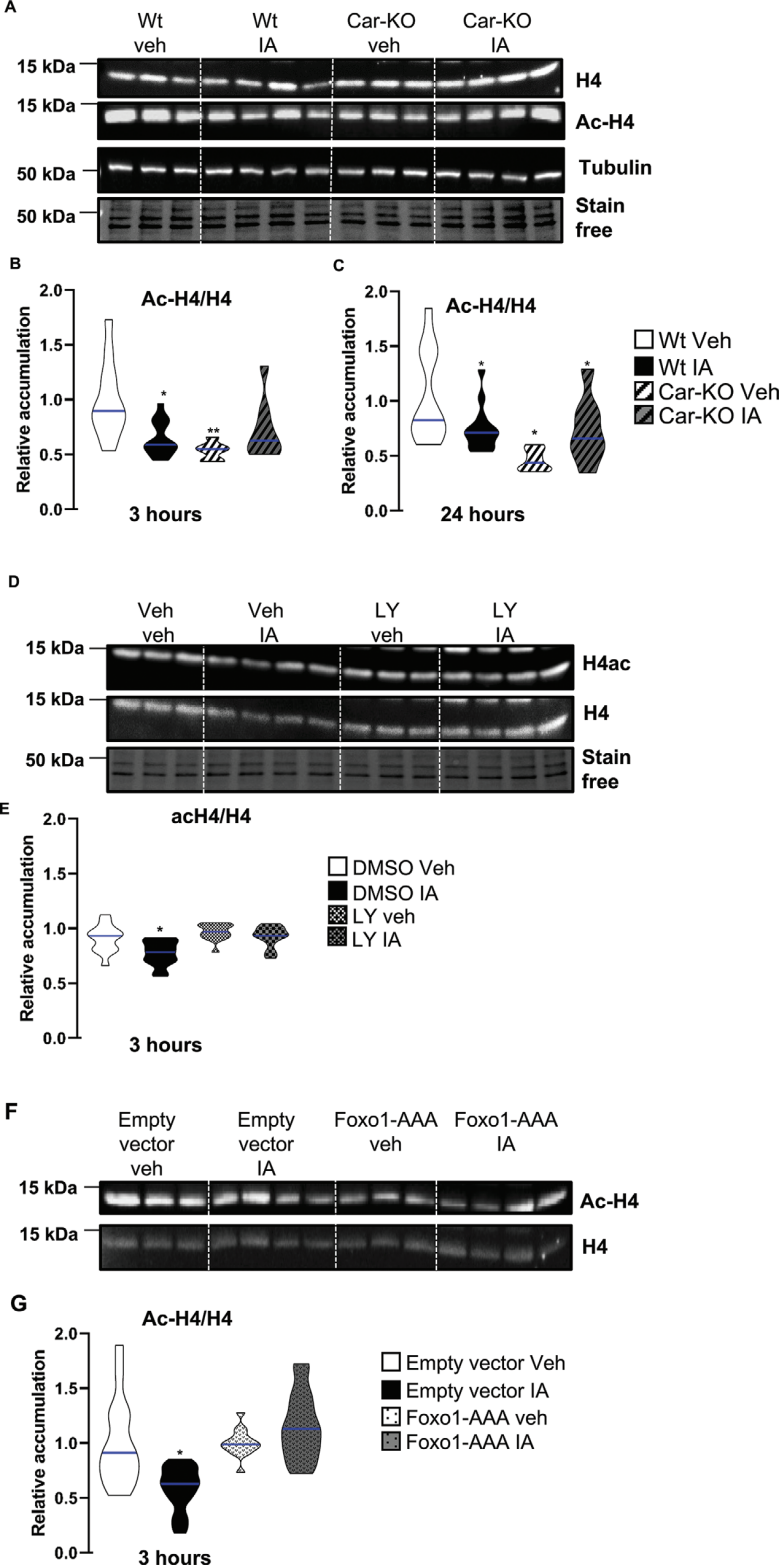
A) Representative western blots of H4 and Ac‐H4 in Wt and CAR‐KO cells treated with vehicle or IA for 3 h. B) Quantification of ratios Ac‐H4/H4 in Wt and Car‐KO cells treated with vehicle or IA for 3 h. Normalization was performed against total protein using stain‐free gels. The blue line indicates the median for each group. Wt vehicle group was arbitrarily set at 1. C) Quantification of ratios acH4/H4 in Wt and Car‐KO cells treated with vehicle or IA for 24 h. Normalization was performed against total protein using stain‐free gels. The blue line indicates the median for each group. Wt vehicle group was arbitrarily set at 1. D) Representative western blots of H4 and Ac‐H4 in Wt cells treated with vehicle or LY (LY294002) for 45 min and then with vehicle or IA for 3 h. E) Quantification of ratios Ac‐H4/H4 in Wt cells treated with vehicle or LY (LY294002) for 45 min and then with vehicle or IA for 3 h. Normalization was performed against total protein using stain‐free gels. The blue line indicates the median for each group. Vehicle groups of each condition (DMSO or LY) were arbitrarily set at 1. F) Representative western blots of H4 and Ac‐H4 in Wt cells transfected with an empty vector or a vector for overexpression of none phosphorylable Foxo1 (Foxo1‐AAA) and treated with vehicle or IA for 3 h. G) Quantification of ratios Ac‐H4/H4 in Wt cells transfected with empty vector or a vector for overexpression of none phosphorylable Foxo1 (Foxo1‐AAA) and treated with vehicle or IA for 3 h. Normalization was performed against total protein using stain‐free gels. The blue line indicates the median for each group. Vehicle groups transfected with empty vector or Foxo1‐AAA were arbitrarily set at 1. In all panels, Veh: vehicle and IA: Inverse agonist. In all panels, n = 25 from six independent experiments. Two‐way ANOVA followed by Holm–Sidak's test for multiple comparisons. B and C: *p < 0.05 versus Wt vehicle‐treated group. In E *p < 0.05 versus DMSO‐vehicle‐treated cells group. In G, *p < 0.05 versus vehicle cells transfected with empty vector.

Although the exact mechanism is unclear, Foxo1 expression alteration in spermatogonia is speculated to result in abnormalities in germ cell development during the post‐meiotic steps.^[^ ^20^, ^32^ ^]^ To explore the potential link between Car inhibition (via IA exposure or gene deficiency), modulation of Foxo1 expression, and alteration in histone H4 acetylation observed in vivo (**Figure** **18**), we examined the role of acetylated histone H4, which plays critical roles in spermatogonium and spermatid homeostasis and differentiation. Our in vitro analyses revealed that the lack of the *Car* gene led to a lower Ac‐H4/H4 ratio in Car‐KO cells than in Wt cells (Figure 18A–C). Similarly, exposure of Wt cells to IA led to a reduced Ac‐H4/H4 ratio compared to vehicle‐treated Wt cells (Figure 18A–C). IA treatment did not affect the *Car*‐KO cells (Figure 18A–C). The reduction in Ac‐H4 levels observed 3 h after IA treatment (Figure 18A–C) suggests that this effect may not be mediated through transcriptional Car activity, but rather through the modulation of more rapid signaling pathways. Pre‐treatment with LY294002 was sufficient to counteract the IA effect on the Ac‐H4/H4 ratio in Wt cells (Figure 18D,E). To define the involvement of Foxo1, we performed experiments using Foxo1‐AAA‐transfected cells. IA treatment did not affect the Ac‐H4/H4 ratio in Foxo1‐AAA‐transfected cells, in contrast to that observed in cells transfected with the empty vector (Figure 18F,G; Figure S19, Supporting Information).

Collectively, our findings explain how the inhibition of Car activity (genetically or pharmacologically) alters spermatogonia homeostasis and subsequent alteration of Ac‐H4 that was associated with the retention of histones in sperm cells and fertility disorders.

## 3 Discussion

In this study, Wt and *Car* ^−^ *^/^* ^−^ mice were utilized to demonstrate that neonatal inhibition of the Car signaling pathway critically impacts germ cell homeostasis and male fertility following exposure to xeno‐ or endobiotics, exemplified here using androstanol as a classical Car inhibitor. This opens intriguing avenues for exploring the molecular mechanisms underlying Car action in the context of inhibitor exposure.

### 3.1 Car Signaling Pathway Altered Neonatal Undifferentiated Germ Cells

The study showed that inhibition of the Car signaling pathway primarily affected undifferentiated spermatogonial cells during neonatal life, transitory altering the establishment of the spermatogonial stem cell pool. Initially, Car inhibition (genetic and pharmacological) impact on gonocyte numbers was observed through Dnmt3l staining. The data demonstrated a higher number of gonocytes in AI‐treated Wt males and *Car* ^−^ *^/^* ^−^ males than in vehicle‐treated Wt males from day 1 to day 5 of life. This partly explains the influence of Car signaling on the gonocyte‐to‐spermatogonia transition, as indicated by an increase in Dnmt3l+ cells and a decrease in Id4+ cells during the neonatal period under Car inhibition. Specifically, a transient decrease in Id4+ cell numbers was observed in IA‐treated Wt males and Car^−/−^ males compared to vehicle‐treated Wt mice. However, a slight delay was observed in this phenotype: it appeared as early as 1 dpn in *Car* ^−^ *^/^* ^−^ males, whereas it was detectable only at 3 dpn in IA‐treated Wttt mice, reflecting the treatment initiation from 1 dpn onwards in the latter group.

As Id4 expression is confined to a rare subset of undifferentiated spermatogonial cells, additional markers, such as Plzf, were analyzed to further delineate the impact of the Car signaling pathway on germ cells. In *Car* ^−^ *^/^* ^−^ males, lower Plzf+ cell numbers were observed at 1 dpn, and no effect was observed at 3 dpn. This pattern mirrored that observed for Id4+ cells in *Car* ^−^ *^/^* ^−^ males, suggesting that Car deficiency affects the pre‐spermatogonia (gonocyte) population. In contrast, IA treatment did not affect Plzf+ cells in Wt males at 3 dpn, unlike its impact on Id4+. Notably, IA treatment had no effect on Lin28a, another spermatogonia marker, at 3 dpn. This difference could be related to the heterogeneity of spermatogonia, as illustrated by the fact that only 90% of Id4+ cells were Plzf+ at 3 dpn (Figure S4A, Supporting Information).^[^ ^46^, ^64^ ^]^ This was also supported by previous data showing that Id4+ spermatogonia only represented a subset of Gfra1+ spermatogonia from 6 dpn to adulthood, identifying the heterogeneity and hierarchy of spermatogonial populations in mouse testes.^[^ ^47^, ^65^, ^66^ ^]^ Thus, the present data suggest that the inhibition of Car may specifically affect Id4+ cells.

The concept of disrupted gonocyte‐to‐spermatogonia transition was substantiated by identifying ectopic Id4+ germ cells and Plzf+ germ cells at 5 dpn. Identifying ectopic germ cells at the center of seminiferous tubules coincided with the altered subcellular localization of Foxo1 in spermatogonia, a crucial factor in the migration of pre‐spermatogonia from the tubule center to the basement membrane during gonocytes‐to‐spermatogonia transition. Notably, Foxo1 showed alterations in response to neonatal modulation of the Car signaling pathway. Vehicle‐treated *Car* ^−^ *^/^* ^−^ males and IA‐treated Wt males exhibited fewer Foxo1+ spermatogonia and alterations in its subcellular and cellular localization than control Wt males. These effects on Foxo1 expression and localization were confirmed using in vitro approaches. The rapid onset of IA treatment's effect on Foxo1 expression from 3 h onward suggests involvement in intracytoplasmic signaling rather than transcriptomic changes. Our results also showed that Car modulation altered the Foxo1 phosphorylation level, which is a prerequisite for subcellular trafficking. This effect on P‐Foxo1 was counteracted by inhibiting Pi3k, suggesting that the crosstalk between Car signaling and the Pi3k/Akt pathway mediates the effects of Foxo1 in spermatogonia. In vitro findings further support that the Car signaling pathway modulates Foxo1 expression and localization via Pi3k/Akt, as demonstrated with the specific inhibitor LY294002. These changes in Foxo1 expression were associated with alterations in mRNA levels of known Foxo1 target genes, such as *Gata2*, *Tex19*.1, and *Gadd45b*. Thus, additional studies are needed to better understand how exposure to Car IAs modulate Foxo1 translocation and altered Foxo1 activity in spermatogonia.

Although not explicitly defined in this study, these differences likely arise from molecular mechanisms specific to Car absence or pharmacological inhibition. Consistently, the in vivo findings on spermatogonial homeostasis align with results obtained in vitro using spermatogonial cell lines. Notably, the distinct downregulation profiles of *Gfra1* and *Id4* at 3 h versus that at 6 and 24 h may be due to specific signaling pathways underlying these downregulations. The process was rapid for the genes that were downregulated within 3 h (*Gfra1* and *Id4*). Hence, we speculated that these could be Car target genes or that these decreases could be due to an interaction with an intracellular signaling pathway, such as Pi3k/Akt, which we have shown here to be modulated by *Car* gene deficiency and IA treatment (see Figure 17).

Our in vitro data suggest that Car regulates mRNA levels of key genes, such as *Nanog*, *Thy1*, *Gfra1*, and *Id4* in undifferentiated spermatogonia, potentially influencing their homeostasis. This provides insight into how Car's absence or inhibition by AI reduces spermatogonial numbers compared to control conditions (Wt cells treated with veh). Additionally, while *Gata2* mRNA levels were altered 6 h after treatment, changes in *Cdh1*, *Tex19.1*, and *Gadd45b* mRNA were observed only after 24 h. These long‐term effects may indicate indirect regulation by Car or dependency on intermediate factors, possibly involving Foxo1, given that these genes are known targets of *Foxo1* whose mRNA levels are influenced by changes in Foxo1 protein expression downstream of Car signaling.

Furthermore, the duration of Car's impact on mRNA accumulation appears gene‐specific, potentially influenced by messenger stability and the complexity of regulatory mechanisms. Genes regulated through direct interactions with intracellular signaling pathways may show short‐term effects, while those affected by both genomic and intracellular signaling pathways may show short‐ and long‐term effects, while those affected only by genomic pathways may exhibit long‐lasting effects mediated by Car.

Even though transient, the early effects on Id4+ cells could potentially impact the establishment of the germ cell lineage. Analysis of Plzf, which is expressed more broadly than Id4 in spermatogonial populations, revealed insights into how Car signaling influences germ cell differentiation. In *Car* ^−^ *^/^* ^−^ males, a reduction in Plzf+ cells was observed at 1 dpn, not at 3 dpn, and again at 5 and 10 dpn. Interestingly, the early phenotype (1 and 3 dpn) was observed only in vehicle‐treated *Car* ^−^ *^/^* ^−^ males compared to that in Wt males. In contrast, similar reductions in the number of Plzf+ cells were observed at 5 and 10 dpn in IA‐exposed Wt compared to vehicle‐treated Wt male mice.

These findings suggest that the Car signaling pathway exerts multiple effects on spermatogonia at different stages of differentiation. The impacts at 1 and 3 dpn likely reflect effects on pre‐spermatogonia (gonocytes), whereas analyses at 5 and 10 dpn likely reflect effects on spermatogonia. This distinction is supported by the greater reduction in Id4‐/Plzf+ cells than in Id4+/Plzf+ cells in IA‐treated Wt males or *Car* ^−^ *^/^* ^−^ males at 10 dpn compared to control Wt males. However, by 15 dpn, germ cell numbers normalized across all groups, suggesting that spermatogenesis returned to a level comparable to control mice. These findings suggest that the major long‐term effect of Car signaling inhibition on male fertility is not due to altered germ cell number. This hypothesis was confirmed in adulthood by the absence of any effect on sperm count in the epididymal head, which represents testicular production.

### 3.2 Car Signaling Pathway Impacted Spermiogenesis, Sperm Cell, and Male Fertility

Our findings demonstrate that, unlike other analyzed markers, alterations in Foxo1 due to neonatal modulation of the Car signaling pathway persist into adulthood. Specifically, fewer Foxo1+ spermatogonia and alterations in its subcellular and cellular localization were still detected in vehicle‐treated *Car* ^−^ *^/^* ^−^ males and IA‐treated Wt males than in control Wt males. These Foxo1 alterations were suspected to alter the late stages of spermatogenesis, namely spermiogenesis. Interestingly, the present data show that Car inhibition (both genetic and pharmacological) had a major impact on the acetylation of histone H4. In vitro experiments showed that the effect of IA on Ac‐H4 levels was rapid (within 3 h). Interestingly, our data showed that this effect was Foxo1‐dependent, as transfection with the constitutive nuclear form of Foxo1 counteracted the IA treatment effects. Additionally, this effect was abolished by treatment with a Pi3k inhibitor. Together, our results suggest that the interaction between Car and Foxo1 was mediated by the Pi3k‐Akt pathway to control histone H4 acetylation.

Identifying the Foxo1‐dependent alteration of Ac‐H4 could partly explain the impact of Foxo1 on the late stages of spermatogenesis, which was previously observed without identifying the molecular mechanisms.^[^ ^20^ ^]^ This Foxo1‐dependent alteration of Ac‐H4 may also contribute to the impaired spermatogenesis observed in this study in the context of inhibition of the Car signaling pathway. This is consistent with the observation of altered spermiogenesis, as revealed by the abnormal ratio of seminiferous tubules with round and/or elongated spermatids detected in IA‐treated Wt males and *Car* ^−^ *^/^* ^−^ males compared to control Wt mice. Consistently, an abnormal histone‐to‐protamine transition was verified by H3 retention in spermatozoa from IA‐treated Wt and *Car* ^−^ *^/^* ^−^ mice compared to control Wt mice. Notably, the level of histone H3 retention in the spermatozoa correlated with the percentage of seminiferous tubules containing Ac‐H4‐positive round cells, which associated testicular events with altered sperm quality.

Alterations in sperm DNA composition, such as histone modifications, might be expected to contribute to sperm head structure abnormalities. Our results align with the observed decrease in sperm count and histone retention, which are the major contributors to sperm head abnormalities. However, a more drastic impact could have been observed in both sperm head and flagellum as observed in the context of protamine default.^[^ ^67^, ^68^ ^]^ Notably, abnormalities of the head predominate over those of the flagellum in most mammals, including humans^[^ ^69^ ^]^ and in our case C57Bl/6J mice.^[^ ^70^ ^]^ This predominance is accentuated in cohorts of infertile men.^[^ ^71^ ^]^ Further, environmental or toxic stresses impact the head before the tail, for example with the diet in our C57Bl/6J model.^[^ ^72^ ^]^ This is also consistent with a recent publication demonstrating that mice knocked out heterozygous 4 genes responsible for flagellum abnormalities and got head abnormalities.^[^ ^73^ ^]^ In this latter study, very few flagella were altered, with a starting rate (10%) that has never really changed despite the increase in the mutational load, unlike the head where accumulated defects as mutations have progressed up to more than 40%. In mice, head abnormalities are a common hallmark in studies of this nature.^[^ ^74^ ^]^ Together, these findings reinforce the current data on the impact of Car signaling pathways on sperm head structure.

Certain sperm alterations may not affect fertility as long as the percentage of normal sperm remains intact. However, the literature suggests that even subtle changes in sperm DNA, such as alterations in histone epigenetic modifications, can lead to transgenerational transmission of abnormalities to offspring. Histone retention in spermatozoa may contribute to idiopathic male infertility, influencing embryonic development and reproductive success. Studies in humans show that differential sperm histone retention in normozoospermic ejaculates from infertile men negatively impacts sperm functional competence and embryo quality. Increased histone H3 levels have been linked to poor embryo quality and clinical pregnancy outcomes after embryo transfer.^[^ ^75^ ^]^ These findings highlight that abnormal histone content in sperm cells affects not only classical sperm characteristics like capacitation, hyperactivation, chemotaxis, acrosome reaction, and fertilization but also embryonic development.

In line with this, our results demonstrate, for the first time, that modulation of the Car signaling pathway during the neonatal period has long‐term effects on fertility in adulthood. We explored several parameters to determine the exact reason for this decrease in male fertility. Our findings indicate that sperm production numbers were largely unaffected, and most spermatozoa showed no significant changes in morphology and motility due to Car signaling pathway modulation. Additionally, the consistent number of embryos per litter observed at E2.5 days post‐coitum across genotypes and treatments suggests that sperm cells retained normal abilities for fertilization processes, such as capacitation, hyperactivation, chemotaxis, and acrosome reaction. Despite this, the negative impact of Car inhibition on male fertility, including an increased incidence of dead pups, suggests an alteration in germ cell quality rather than quantity. This conclusion is supported by the correlation between histone H3 levels in spermatozoa and the higher rate of dead offspring in litters.

### 3.3 Identified Limitations of the Present Study

Although we clearly demonstrated the impact of the Car signaling pathway on germ cells and suggested direct effects based on our in vitro experiments with spermatogonial cell lines, we cannot completely exclude its impact on somatic testicular cells. Spermatogenesis relies on the interaction between germ and somatic cells, such as Sertoli cells. These results provided structural and nutritional support for germ cell differentiation. The correlation between Sertoli cell number, testicular size, and sperm output is well‐established. Sertoli cells regulate key mechanisms involved in the phenotypes observed following modulation of the Car signaling pathway. Sertoli cells produce factors involved in maintaining gonocyte quiescence until birth. In the postnatal testis, Sertoli cell factors, such as Kit ligands or chemokines, regulate gonocyte migration toward the base of the seminiferous tubules. Simultaneously, Gdnf (Glial derived nerve factor), Fgf2 (Fibroblast groWth factor), and Cxcl12 (C‐X‐C motif chemokine 12) produced by Sertoli cells stimulate gonocyte proliferation, and Gdnf, Lif (leukemia inhibitory factor), and Fgf2 act on SSC receptors to promote lifelong maintenance. Sertoli cells play an important role in meiosis entry by regulating retinoid metabolism. In addition, Sertoli cells protect germ cells by forming a testicular‐blood barrier and support spermatogenesis by producing various factors required by germ cells, such as glucose metabolism to lactate. Additionally, germ and Sertoli cells interact closely with each other. Thus, the number of adult Sertoli cells dictates the number of meiotic and post‐meiotic germ cells in the testes. Post‐meiotic germ cells cross the epithelium during spermiogenesis in association with Sertoli cells via ectoplasmic specialization until the spermiation stage. In our study, no effect of IA treatment on the number of Sox9+ Sertoli cells was observed at 5 dpn (data not shown) or in adulthood. Moreover, in the present study, the involvement of Sertoli cells following the modulation of the Car signaling pathway was not investigated, as immunohistochemistry experiments showed that Car was not expressed in Sertoli cells.

Although the molecular mechanisms for the direct or indirect activation of Car are well‐described, only a few studies have focused on the mechanisms underlying its inhibition. An IA drug binds to a receptor as an agonist but induces an opposite response. Thus, its effects are expected to differ from those observed upon knockout, where the receptor is absent and thus cannot interact with either coactivators or corepressors. This is complicated to reconcile for Car, as this nuclear receptor has established a “reverse” paradigm of nuclear receptor action, in which the receptor is also active in the absence of the ligand. Thus, in the case of Car, it can be hypothesized that when it is bound to IAs, its inhibition mimics what occurs in the absence of a receptor. The present data show that the inhibition of Car activity, either by using a genetic invalidation strategy (*Car*‐knockout mice or CRISPR/Cas9 Car‐KO cell line) or a pharmacological approach (use of IA), mostly led to similar effects within the testis and germ cells.

Additionally, at the molecular level, additional data regarding the role of Car in undifferentiated germ cells should be collected. As Car is a nuclear receptor and the consensus sequences of Car response elements have been identified, Chip‐seq approaches combined with transcriptomic analysis on spermatogonia cells would be interesting to determine its target genes in this context. This will pave the way for validating research avenues aimed at elucidating the molecular functions of Car within the germ line. Similarly, while the roles of Foxo1 in spermatogonial homeostasis have been well defined,^[^ ^20^ ^]^ characterizing Foxo1 target genes in spermatogonia, combining ChIP‐seq and transcriptomic analyses would be crucial. Notably, some genes studied in our research, such as Gata2 and Tex19, identified as common targets of Car and Foxo1, were found to be dysregulated in the testis of Foxo1 null mice.^[^ ^20^ ^]^ To further elucidate the relationship between Car and Foxo1, cross‐analysis of the data on the target genes of Car and Foxo1 will enable us to identify their common target genes in the context of undifferentiated germ cells. This approach will help define the common functions they regulate in undifferentiated spermatogonia.

Furthermore, the interaction between Car and Foxo1 within germ cells raises questions, as various mechanisms have been proposed, primarily observed in liver cells: Car can compete with Foxo1 for binding to response elements on the promoters of *Pepck* and *G6pase*.^[^ ^54^ ^]^ Moreover, Car has been shown to act as a co‐repressor of Foxo1 and can diminish its transcriptional activity through nuclear interaction with the Foxo1 protein, thereby reducing Pepck and G6Pase gene expression.^[^ ^54^, ^76^ ^]^ Additionally, we should be cautious that few differences could exist between data from cell lines, spermatogonia sorted in vivo, or primary cell cultures. However, in this study, phenotypic data, such as spermatogonia count and effects on Foxo1+ cells and *Foxo1* mRNA, were consistent between mouse models and immortalized cell lines, underscoring the reliability of our findings.

Several xenobiotics can modulate Car activity, and our present data were obtained using only a specific IA. Therefore, we cannot extrapolate the differences that might be observed with other IA molecules. Additionally, our experiments were performed using a single molecule, whereas humans are exposed to multiple molecules simultaneously. The responses observed in mice might differ from those in humans due to variations in Car affinity for exogenous molecules among species. These limitations need to be addressed in future experiments.

## 4 Conclusions and Relevance of the Study

The analyses of the actors that can play roles in male testicular physiology and physiopathology are critical as the incidence of various male reproductive disorders, such as cryptorchidism, testicular cancer, and low sperm count, has gradually increased in some parts of the world over the last few decades.^[^ ^77^, ^78^ ^]^ These abnormalities may be related, at least in part, to increased exposure to environmental pollutants. Experimental rodent models have demonstrated that the fetal and neonatal periods are critical windows of sensitivity to toxic substances. This is mainly related to the fact that these are critical developmental windows for the testis and that alterations in testicular physiology during these periods could lead to fertility disorders in adults. The presence of the Car signaling pathway in the testis and its known roles in response to xenobiotics highlight that it could be involved in testicular pathologies in the context of the developmental origin of health and diseases.

Thus, the present data are relevant because exposure to xenobiotics is associated with an increased risk of persistent azoospermia. Genetic factors contribute to ≈15–30% of male infertility cases, which suggests the potential major impact of environmental exposure to xenobiotics. Thus, our findings are even more relevant, as ≈1 in 20 men is subfertile or infertile.^[^ ^13^ ^]^ Moreover, idiopathic infertility is the most common individual diagnosis of male infertility, representing ≈44% of cases,^[^ ^14^ ^]^ highlighting the urgent need to identify the causes of male fertility disorders. The present work helps us further understand fertility disorders in the context of environmental exposure to xenobiotics and provides new perspectives for developing diagnostic/prognostic markers and/or therapeutic solutions targeting Car for fertility disorders and promoting the germ cell lineage. Therefore, the side effects of xenobiotics on quality of life, including the maintenance of fertility, are key issues that need to be addressed. Therefore, pursuing research programs to decipher underlying molecular mechanisms involved in minimizing long‐term xenobiotics side effects, particularly those on fertility, is crucial. Although further research is required to elucidate certain molecular aspects, the main goal of this study was to define whether modulation of Car signaling pathway by exogenous molecules could drive the impacts of these xenobiotics on male fertility. The current body of data indicates that inhibiting Car signaling has phenotypic consequences, associated with altered homeostasis of the germ cell line up to spermatozoa. This, in turn, results in male fertility disorders. Hence, inhibiting the Car signaling pathway could pave the way for a better understanding of the impact of environmental molecules on reproduction and could open new perspectives for treatment solutions for fertility disorders.

## 5 Experimental Section

### Animals

This study was conducted following current regulations and standards approved by Institut National de la Santé et de la Recherche Médicale Animal Care Committee and by the animal care committee (CE2EA Auvergne; APAFIS#16680‐2020072216408245 v1).

Mice were housed in temperature‐controlled (22 °C) rooms with 12‐h light/dark cycles and had ad libitum access to food and water. Animal welfare was ensured through enriched cages (including cardboard tunnels and mouse houses) and social housing in groups of five mice per cage, complying with legislation on cage size relative to the number of mice.

Male and female C57Bl/6J mice, aged 7 weeks, were purchased from Charles River Laboratories (L'Arbresle, France) and acclimatized for at least 2 weeks before breeding to generate the wild‐type litters used in the experiments. *Car^−/−^* mice were previously described^[^ ^18^ ^]^ and maintained in a C57BL/6J background.

To minimize confounders in the analysis of the mice, several independent experiments were conducted, and animals were euthanized in different order across experiments. The number of samples per group was determined based on multiple factors: our expertise with genetically modified animal models and pharmacological approaches (using references), pilot studies on C57bl6J mice treated with the inverse agonist to understand effects and inter‐individual variability on histological and reproductive parameters, and validation of data using GIGA's power and sample size calculation free software.

Depending on the experiment, organs were harvested at various time points: 1, 3, 5, 10, 20, 30, 40, 90, and 180 d postnatal, or at 8 months of age. The time points were selected based on the following criteria: from birth to 6 d postpartum, gonocytes (pro‐spermatogonia) migrate from the center of the seminiferous tubules to the basement membrane,^[^ ^14^, ^25^ ^]^ transforming into spermatogonial stem cells (SSCs), which sustain spermatogenesis from puberty to adulthood. The 10‐dpn time point was analyzed as it corresponded to the end of the treatment, and the 15‐dpn time point was analyzed as it was when the blood‐testicular barrier functionally defined the basal and apical compartments in the seminiferous tubules. For adult analyses, some models demonstrated that young adult mice can be fertile and that reduced fertility was only observed in older mice, which resulted from a slight and constant loss of SSCs. This was evident in inhibitor of differentiation 4 (*Id4*)‐knockout males, whose reproductive capacities diminished at ≈8 months of age.^[^ ^64^ ^]^ Thus, to identify the potential impacts of Car expression (genetic) or activity (pharmacological) modulations on male fertility, mice were followed up until 8 months of age.

The number of animals per group was defined on independent experiments to validate the reproducibility of results.

### Analysis of Car Expression Using Magnetic Cell Sorting in Wt Male Mice

Testis cell suspensions from adult Wt males were used for spermatogonia using Thy1 (Miltenyi Biotec) antibodies conjugated to MACS microbeads (Miltenyi Biotec). Briefly, testes from 6‐d‐old mice (n = 4) were used for neonatal analysis, and testes from individual 3‐month‐old mice were used for adult experiment. Then, albuginea was removed, and seminiferous tubes were unwound into a dish containing 1.5 mL of M2 medium. Collagenase (20 mg mL^−1^, 50 µL) was added and incubated for 20 min at 37 °C with agitation until tubes were dissociated. After removing most supernatant, 250 µL of 0.5% trypsin + 1 µL of DNAse (100 mg mL^−1^) was added to the remaining 750 µL medium and incubated for 5 min at 37 °C with agitation to ensure cell dissociation. Next, 500 µL SVF was added and centrifuged for 10 min at 300 g at 4 °C. The supernatant was removed, and cells were resuspended in 1 mL of sorting buffer and centrifugated for 10 min at 300 g at 4 °C. Supernatant was removed, and cells were resuspended in 80 µL sorting buffer with 20 µL of the desired biotinylated antibody. The resultant solution was incubated for 15 min in a rotating cold chamber. 1 mL of sorting buffer was added to dilute antibody and then centrifuged for 10 min at 300 g at 4 °C.

The supernatant was removed, and cells were recovered in 80 µL of sorting buffer. 20 µL of anti‐biotin magnetic beads were added and then incubated for 15 min in a cold room on rotation. Then, 1 mL of sorting buffer was added to dilute beads and centrifuged for 10 min at 300 g at 4 °C. The supernatant was then removed, and cells were recovered in 1 mL sorting buffer. After centrifugation for 10 min at 300 g at 4 °C, the supernatant was removed and cells were resuspended in 500 µL sorting buffer. The 500‐µL suspension was applied to an equilibrated MACS MS column. The unretained fraction, corresponding to the unsorted cells, was collected. Thus, the eluates contain all other cell types of the testis (interstitial and tubular fractions). Columns were washed twice, and the column was removed from the magnetic rack and placed on an empty 1.5 mL tube. Then, 1 mL of sorting buffer was added, and the liquid was pressed with a plunger to recover the sorted fraction. The eluates of sorted cells, as well as the previously collected unsorted cells, were centrifugated for 10 min at 300 g at 4 °C. The supernatant was removed, and cells were frozen for mRNA analysis.

### Targeting of Car Pathway Using Car^−/−^ mice and inverse agonist exposure

Newborn mice were exposed with DMSO or androstanol (IA) (25 mg kg^−1^) subcutaneously from 1 to 10 d postnatal (5 µL d^−1^). The mice were housed at a density of 5 per cage, except after reproductive capacity tests, when male mice were separated and placed individually in enriched cages prior to euthanization.

Protocols were previously conducted in adult mice without reported suffering throughout our experiment. Daily monitoring in the initial days post‐injection included checks for signs of distress, abnormal activity, or physiological changes, such as increased weight loss. No instances of suffering were observed.

### Fertility Test

Fertility was assessed through multiple rounds of fertility tests between 6 and 7 months of age. Males were euthanized 2 weeks after the final fertility test at 8 months of age. During each fertility test, males were paired nightly with C57Bl6J females (Charles River) for 15 d. Mating was monitored daily for vaginal plugging to confirm mating occurrence. After 2 or 14 d post‐mating, mating efficiency was evaluated, and the number of pups per litter was recorded.

### Sperm Analyses—Sperm Phenotyping

Evaluation of sperm parameters was performed according to protocols from Martinez et al. 2022.^[^ ^79^ ^]^ Briefly, sperm concentration was evaluated on the Thoma Cell Counting chamber (VWR, catalog number: HECH40447702). Sperm motility was evaluated with a Computer Assisted Sperm Analysis System CEROS II (Hamilton Thorne Research, IMV Technologies, catalog number: 024905) using Counting chamber Leja 100 µm (Leja Products B.V., Gynemed, catalog number: SC100‐01‐02‐A). Morphology analysis was manually performed on sperm slides after Harris–Schorr coloration (minimum 200 sperm cells per mouse). Pictures were acquired on a Nikon Eclipse 80i microscope equipped with a Nikon DS‐Ri1 camera and NIS‐Elements software.

### Sperm Analyses—Transmission Electron Microscopy (TEM)

TEM experiments were performed like previously^[^ ^80^ ^]^ using sperm cells from wild‐type and knock‐out mice, with and without treatment. Briefly, following glutaraldehyde fixation, the sperm pellet was washed in fresh buffer and embedded in 2% agar. Post‐fixation was performed using 1% osmic acid in phosphate buffer. Samples were subsequently dehydrated and further embedded in Epon resin (Polysciences Inc., Warrington, PA, USA). Sections were cut on a Reichert OmU2 ultramicrotome (Reichert‐Jung AG, Vienna, Austria) with a diamond knife. Ultrathin sections (70 nm) were collected on Parlodion 0.8%/isoamyl acetate‐coated 100 mesh Nickel grids (EMS, FortWashington, PA, USA) and counterstained with 2% uranyl acetate and lead citrate before observation. Sections were examined with a Zeiss transmission electron microscope 902 (Leo, Rueil‐Malmaison, France). Images were acquired using a Gatan Orius SC1000 CCD camera (Gatan France, Grandchamp, France).

### Sperm Analyses—Nuclear Morphology Analysis

Nuclear morphology was precisely evaluated using Nuclear Morphology Analysis Software (NMAS, version 2.0.0, https://bitbucket.org/bmskinner/nuclear_morphology/wiki/Home), according to the analysis method described in Skinner et al., 2019.^[^ ^55^, ^81^ ^]^ Briefly, DAPI‐stained nuclei were captured with a Zeiss Imager Z2 microscope using a CoolCube 1 CCD camera, with a 100 x/1.4 Zeiss objective and Neon software (MetaSystems, Altlussheim, Germany). Nucleus detection settings were Kuwahara kernel: 3, and flattening threshold: 100, for preprocessing; canny low threshold: 0.5, canny high threshold: 1.5, canny kernel radius: 3, canny kernel width: 16, gap closing radius: 5, to find objects; and min area: 1000, max area: 10 000, min circ: 0.1, max circ: 0.9, for filtering. After acquiring nuclei images, landmarks were automatically identified using a modification of the Zahn–Roskies transform to generate an angle profile from the internal angles measured around the periphery of the nuclei. Angles were measured at every mouse pixel around the original shape. This method combines data from every possible polygonal approximation into a single unified trace, from which landmark features can be detected. Angle profiles were presented as angle degrees according to the relative position of each pixel along the perimeter, and variability profiles use the interquartile range (IQR, difference between the third and first quartile) as a dispersion indicator to measure the variability of values obtained for each point. The statistics relating to nuclear morphology presented in Figure B were automatically calculated using the software. This analysis relied on a Mann–Whitney U test with Bonferroni multiple testing correction. p‐values were considered significant when inferior to 0.05.

### Sperm Analyses—Histone Extraction from Spermatozoa

Sperm cells were extracted from cauda epididymis, which were perforated with a need and incubated for 10 min at 37 °C in M2 medium to allow sperm to swim up. Sperm cell pellets were snap‐frozen in liquid nitrogen and stored at −80 °C prior to use. The acid extraction of histone from mouse spermatozoa was performed according to the protocol adapted from Crespo et al.^[^ ^82^, ^83^ ^]^ Five million mouse spermatozoa were resuspended in 50 mm DTT and incubated for 30 min at 4 °C. Subsequently, 1 mL of 0.4 M H_2_SO_4_ was added and incubated for 1 h on ice. The samples underwent sonication (10 s “on” + 10 s “off” for 12 cycles at high intensity) and were kept on ice for 1 h and 30 min before being centrifuged for 10 min at 14 000 g. Trichloroacetic acid (TCA) was added to the supernatant and incubated for 30 min on ice. Samples were centrifugated for 15 min at 14 000 g at 4 °C, and the pellet was washed with 400 µL of acetone containing 0.05% HCl. After another centrifugation step (15 min, 14 000 g at 4 °C), the pellet was dried under the hood for 10 min and resuspended in Blue loading dye containing 10% *β*‐mercaptoethanol, followed by heating at 70 °C for 5 min. Electrophoresis was performed in polyacrylamide gels at 120 V in a denaturing buffer containing 25 mM Tris Base, 190 mM glycine, and 0.1% SDS. Quantification was performed against total protein content using stain‐free staining, as described above, to ensure accuracy.

Membranes were blocked for one h in a 10% BSA solution in Tris‐buffered saline with 0.1% Tween, followed by overnight incubation with primary antibodies diluted in Tris‐buffered saline with 0.1% Tween and 5% BSA. The list of antibodies used was provided in the Supporting Information (Table S4, Supporting Information). After incubation, membranes underwent five washes with 1X‐tween 0.1% TBS, followed by blotting with fluorophore‐coupled secondary antibodies (IgG (H+L) DyLigh, specific for the species corresponding to the IgG of the primary antibodies (Table S4, Supporting Information). The signals obtained were quantified using Bio‐Rad Image Lab software.

### Testicular Histology

The testes were collected and fixed in 4% paraformaldehyde (PFA) for a minimum of 4 h for testes collected from 1 dpn to 5 dpn and for 24 h for testes collected from 10 dpn to adulthood. Testes were then washed in 70% ethanol at room temperature for 30 min, dehydrated, and embedded in paraffin. Analyses were performed on 5 µm‐thick sections.

### Testicular Immunohistochemistry

It mounted 5‐µm sections on positively charged glass slides (Superfrost plus), deparaffinized, rehydrated, treated for 20 min at 93–98 °C in 0.01 m citric buffer‐tween 0.1% (pH 6), rinsed in osmosed water (2 × 5 min), and washed (2 × 5 min) in phosphate‐buffered saline (PBS). Slides were then counterstained with Hoechst medium (1 mg mL^−1^) and then mounted on PBS/glycerol (50/50). The antibodies used are reported in Table S5 (Supporting Information).

To further validate antibodies, co‐immunostainings were performed (Figures S2 and S20–S23, Supporting Information). Notably, according to the literature, some markers of germ cell lineage Id4, Plzf, Lin28, G9a, or cKit overlap. Present data (Figures S20–S23, Supporting Information) show specific staining according to their expected profiles through the ages studied. Almost all ID4 cells were all Plzf+. For Plzf positive cells, the co‐staining changes with development (Figure S4, Supporting Information) were consistent with previously published data described.^[^ ^46^ ^]^

From 10 dpn, Plzf also overlaps in part with G9a+ cells. Notably, G9a+ cells that were Plzf‐ were cKit (Figure S23, Supporting Information). This was consistent with previously published reports showing that G9a‐positive was expressed from A1 spermatogonia until the leptotene spermatocyte stage.^[^ ^48^ ^]^

As expected, no overlap of the staining of germ cell markers and the Sertoli cell marker SOX9 with the germ cell‐specific markers was observed (Figures S20–S23, Supporting Information).

### Analysis of Testicular Apoptosis using TUNEL

TUNEL experiments were performed, as previously described^[^ ^84^ ^]^ on 5 µm of testis fixed in paraformaldehyde 4%. After deparaffinization of the slides, 8 min of unmasking was carried out in a microwave oven at 500 Watts in a 10 mm pH 6 citrate buffer −0.05% Tween. After washing in 1X PBS, the sections were incubated for 1.5 h at 37 °C, with the solution allowing the enzymatic reaction (dATP [26.6 µm]; biotin‐11‐dUTP [6.6 µm], Terminal Deoxynucleotidyl Transferase [0.3 U], Buffer [1X] per slide). The revelation was performed by the addition of an alkaline phosphatase coupled to extravidin for 25 min then by the addition of its substrate Sigma FastRed Tr/Naphtol AS‐MX (Sigma–Aldrich). A counterstain with hematoxylin (Diapath, Italy) was carried out to visualize the structure of the tissues. Positive cells were counted, and the results were expressed as the relative number of TUNEL positive cells per seminiferous tubules.

### In Vitro Cell Line Approaches

The immortalized wild‐type type cell line of A‐spermatogonia, namely C_18_4 cells (were obtained from Dr Froment, France, who obtained them from Pr Hofmann).^[^ ^56^ ^]^ This immortalized cell line was generated using the Simian virus large T‐antigen gene. This cell line has been demonstrated to express germ cell markers, such as *Dazl*, *Pou5f1* and *Gfrα−1*.^[^ ^56^ ^]^ The cell line deficient of the Car gene, namely Car knockout cells (Car‐KO), was generated as described below:

### In Vitro Cell Line Approaches—Generation of Crispr/CAS9 Car‐Deficient C18‐4 Cells

For the generation of the Car knockout cells using Crispr/CAS9 approach (see Figure S2, Supporting Information), guides were defined based on the mm9 version of the mouse genome and using the website. The sequences (Table S3, Supporting Information) used were for guide‐1 were: AGCCCTCACAAGTCAGGGCG and for guide‐2: TAGTGTTAGCATAGCTGTCA. Then, the guides were introduced on PX458: pSpCas9(BB)−2A‐GFP (PX458) (Addgene Plasmid #48 138) and PX459: pSpCas9(BB)−2A‐Puro (PX459) V2.0 (Addgene Plasmid #62988). pSpCas9(BB)−2A‐Puro (PX459) was a gift from Feng Zhang (Addgene plasmid # 48139; http://n2t.net/addgene:48139; RRID:Addgene_48139).^[^ ^85^ ^]^ Guides were cloned on vectors at the BbsI restriction site.

C18.4 cells were plated on 6‐well plates and transfected using Jet PEI (Ozyme) with 1 µg of guide 1 in PX458 (expressing GFP) and 2 µg of guide 2 in PX459. After 24 h of transfection using GFP expression, C18 cells were sorted via FACS (BD FACSMelody Cell Sorter from BD Biosciences) and plated as individual cell in a 96‐well plate in C18 cell conditioned medium (DMEM and 1% SVF). The clones were then validated by sequencing and genotyping. Primer sequences for PCR are listed in Table S6 (Supporting Information).

### In Vitro Cell Line Approaches—Non‐Transfected Cells Treatment with Inverse Agonist

Wt or CAR‐KO cells were plated in 6‐well plates (50 000 cells per well). After 24 h of plating, cells were treated with vehicle (DMSO, 1/1000) or IA 10^−6 ^mm. Then, cells were harvested at different time points later, and messenger RNA (mRNA) or protein extractions were performed.

### In Vitro Cell Line Approaches—Transient Transfection for Overexpression

C18.4 cells were transfected with Jet‐PEI RNA (Ozyme, Saint Quentin Yvelines, France) in 6‐well plates (50 000 cells per well). The plasmids vector of FOXO1‐AAA (Addgene) or empty vector were transfected at 200 ng per well.

### In Vitro Cell Line Approaches—BrdU Incorporation

To define proliferation rate, cells were plate in 6‐well plates (50 000 cells per well) and were treated with IA or DMSO for 24 h. Cells were then incubated with BrdU (10 µm) for the one last h. Then, cells were washed with PBS1X and then fixed with methanol. The detection of BrdU was performed using primary antibody anti‐Bromodeoxyuridine (11170376001, Merck) revealed with specific Alexa488‐coupled secondary antibody.

### In Vitro Cell Line Approaches—TUNEL

For TUNEL experiments, cells were plate in 6‐well plates (50 000 cells per well) and were treated with IA or DMSO for 24 h. Then, cells were fixed with PFA 1% on 6‐well plates and then the experiments were performed as described above in the immunohistochemistry section, except that the revelation of the staining was visualized via immunofluorescence.

### In Vitro Cell Line Approaches—Cell Fraction Experiments

The preparations of cell fractions were performed as previously described.^[^ ^86^ ^]^ Cells were initially plated at 5 million cells per 10 cm plate. Cells were treated with IA or DMSO for the requested time. At the end of the treatment, cells were harvest from each plate into fractionation buffer (Hepes 10 mm; KCL 10 mm; MgCl2 1.5 mm; Sucrose 0.34 mm; and 10% glycerol). Then, using 1 mL syringe pass cell suspension through a 27‐gauge needle 15 times (or until all cells were lysed) and incubated on ice for 20 min. Cells were centrifuged for 5 min at 4 °C at 1300 rpm. The pellet was the nuclear fraction (revealed using Histone H3). The supernatant (cytoplasmic fraction, revealed using Gapdh) was re‐centrifuged (15 000 rcf, 3 min) to pellet debris. (See Table S4, Supporting Information for antibody details).

### Western Blotting

Proteins were extracted from cells using RIPA lysis buffer completed with protease inhibitors (Roche Diagnostics, Meylan, France). After a sonication step in chilled water bath (high intensity; two times of 30 s ON and 30 s OFF between each sonication; Bioruptor, Diagenode). The proteins were then transferred to denaturing buffer (4x Laemmli Sample Buffer, Biorad Laboratories, USA) for separation via SDS‐PAGE and then transferred to nitrocellulose membrane. Subsequently, 20 mg of protein per well was used for analysis. Electrophoresis was performed in polyacrylamide gels at 120 V in denaturating buffer containing 25 mm Tris Base and 190 mm glycine, 0.1%. The antibodies used are listed in the Table S3 (Supporting Information). For some analyses, the quantifications of the protein accumulation were performed either against houskeeping gene (Tubulin see Table S4, Supporting Information) or against total stained proteins using stain‐free imaging technology from BIO‐RAD. This allows obtaining quantitative western blot data by normalizing bands to total protein in each lane. On each figure of the present manuscript using stain‐free gels, a representative image of total stained proteins on membrane was given with a highlight made with a crop section at ≈50 kDa for each experiment. On western blot experiments, the band sizes and size ladder were included.

### Real‐Time RT‐PCR

RNA was isolated using RNAzol. cDNA was synthesized from total RNA with the MMLV and random hexamer primers (Promega, Charbonnières‐les‐Bains, France). The real‐time PCR measurement of individual cDNA was performed using SYBR green dye (Master mix Plus for SYBR Assay, Takara Bio) to measure duplex DNA formation with the Eppendorf‐Realplex system. For each experiment, standard curves were generated with pools of cDNA from cells with different genotypes and/or treatments. The results were analyzed using the ΔΔct method. Primer sequences are reported in Table S7 (Supporting Information).

### Statistical Analyses—Pre‐Processing of Data

For some in vivo experiments, raw number of positive cells was given for each group in dedicated graphs and then to analyze the impact of pharmacological molecules compared to vehicle groups; vehicle groups of each genotype have been arbitrarily set at 1.

### Statistical Analyses—Data Presentation

All numerical data were represented as mean ± SD. Significant difference was set at P < 0.05.

### Statistical Analyses—Values for n Representing Biological Replicates for Cell Experiments

For animal experiments, n corresponds to the number of animals per condition. For both cell and animal approaches, N indicates the number of independent experiments. For each statistical analysis, the number, type of replicates (e.g., number of samples (n), and number of independent experiments (N)) were reported in the figure legends for each experiment.

### Statistical Analyses—Statistical Methods

Quantitative values were presented as mean± SD. Differences between groups were determined via T‐test or two‐way ANOVA. When overall tests were significant (*p* < 0.05), Holm‐Sidak's post hoc testing was conducted for adjustment, for two‐way ANOVA. For fertility analysis, a Chi2 test was performed. For Correlation analysis, the Pearson's test was performed. Statistical analyses were conducted using Sigmastat 3.2 software as well as Prism 8.0.1. Significant differences between the two groups (^*^ *p* < 0.05, ^**^ *p* < 0.01, ^***^ *p* < 0.001) were defined.

## Conflict of Interest

The authors declare no conflict of interest.

## Supporting information

Supporting Information

## Data Availability

The data that support the findings of this study are available from the corresponding author upon reasonable request.
