# Supplementary material for: Constitutive Androstane Receptor Regulates Germ Cell Homeostasis, Sperm Quality, and Male Fertility via Akt‐Foxo1 Pathway
Source: Adv Sci (Weinh). 2024 Sep 24;11(43):2402082. doi: 10.1002/advs.202402082 (PMC11578384; doi:10.1002/advs.202402082)
Supplement: Supplementary file 1 — Supporting Information [file ADVS-11-2402082-s001.docx]

Supporting Information

Constitutive Androstane Receptor Controls Undifferentiated Germ Cell Homeostasis, Sperm Quality, and Male Fertility through Akt-Foxo1 signaling pathway

Mélusine Monrose, Hélène Holota, Guillaume Martinez, Christelle Damon-Soubeyrand, Laura Thirouard, Emmanuelle Martinot, Edwige Battistelli, Angélique de Haze, Stéphanie Bravard, Christelle Tamisier, Françoise Caira, Charles Coutton, Anne-Laure Barbotin, Angèle Boursier_,_ Lakhal Laila, Claude Beaudoin*, and David H Volle*

**
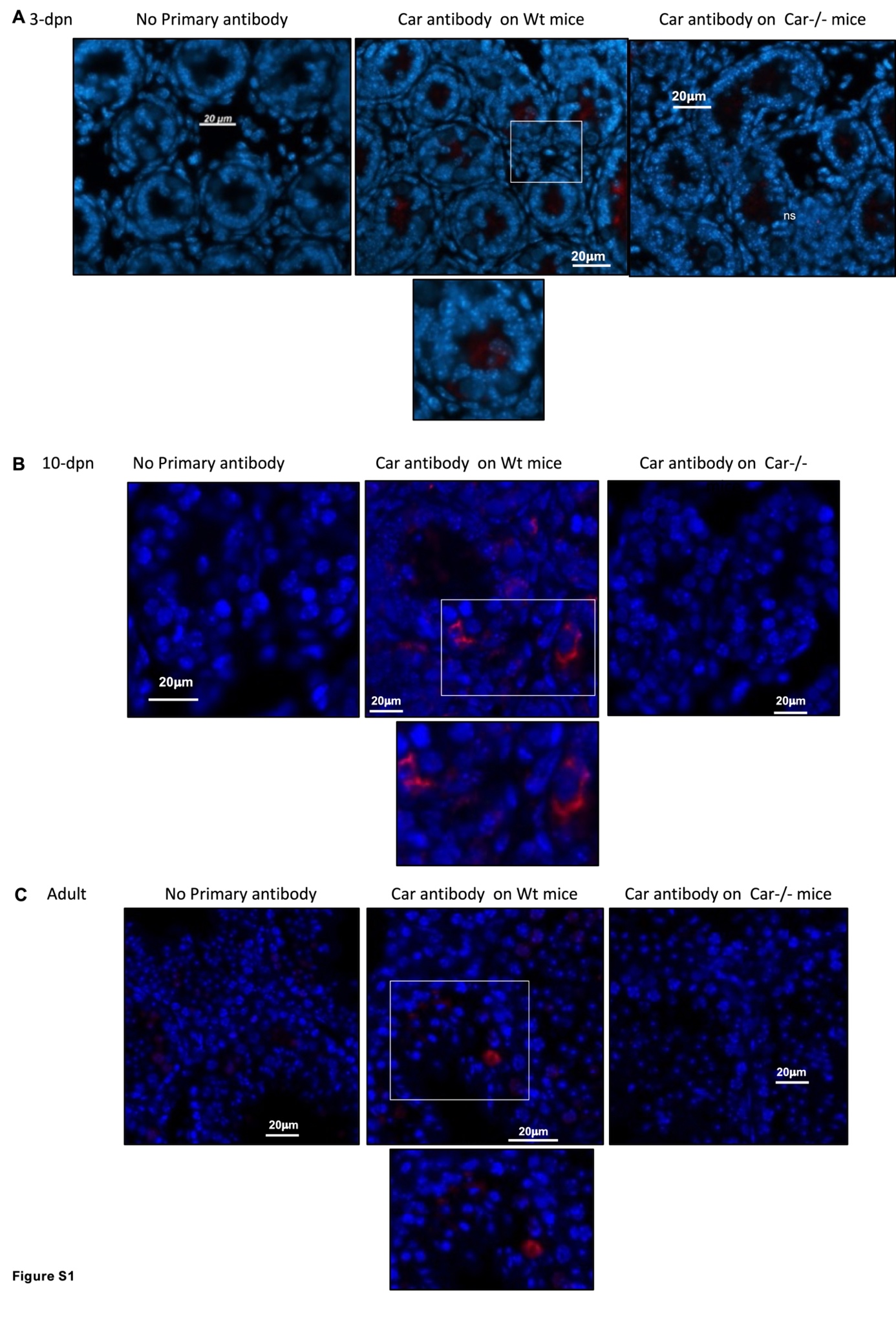
**

**Figure S1**

Validation of Car immuno-histochemistry on testis.

1. Representative testis micrographs of 3-dpn, (Left panel) slides with no primary antibody were prepared on Wt males as a negative control; (Middle panel) immunostaining with CAR antibody was performed on Wt males; (Right panel) immunostaining with CAR antibody was performed on Car-/- males. White square indicates the enlarged area. The experiment was performed on three different male mice. ns: non specifi.
2. Representative testis micrographs of 10-dpn, (Left panel) slides with no primary antibody were prepared on Wt males as a negative control; (Middle panel) immunostaining with CAR antibody was performed on Wt males; (Right panel) immunostaining with CAR antibody was performed on Car-/- males. White square indicates the enlarged area. The experiment was performed on three different male mice. ns: non specific.
3. Representative testis micrographs of Adult mouse, (Left panel) slides with no primary antibody were prepared on Wt males as a negative control; (Middle panel) immunostaining with CAR antibody was performed on Wt males; (Right panel) immunostaining with CAR antibody was performed on Car-/- males. White square indicates the enlarged area. The experiment was performed on three different male mice. ns: non specific.

**
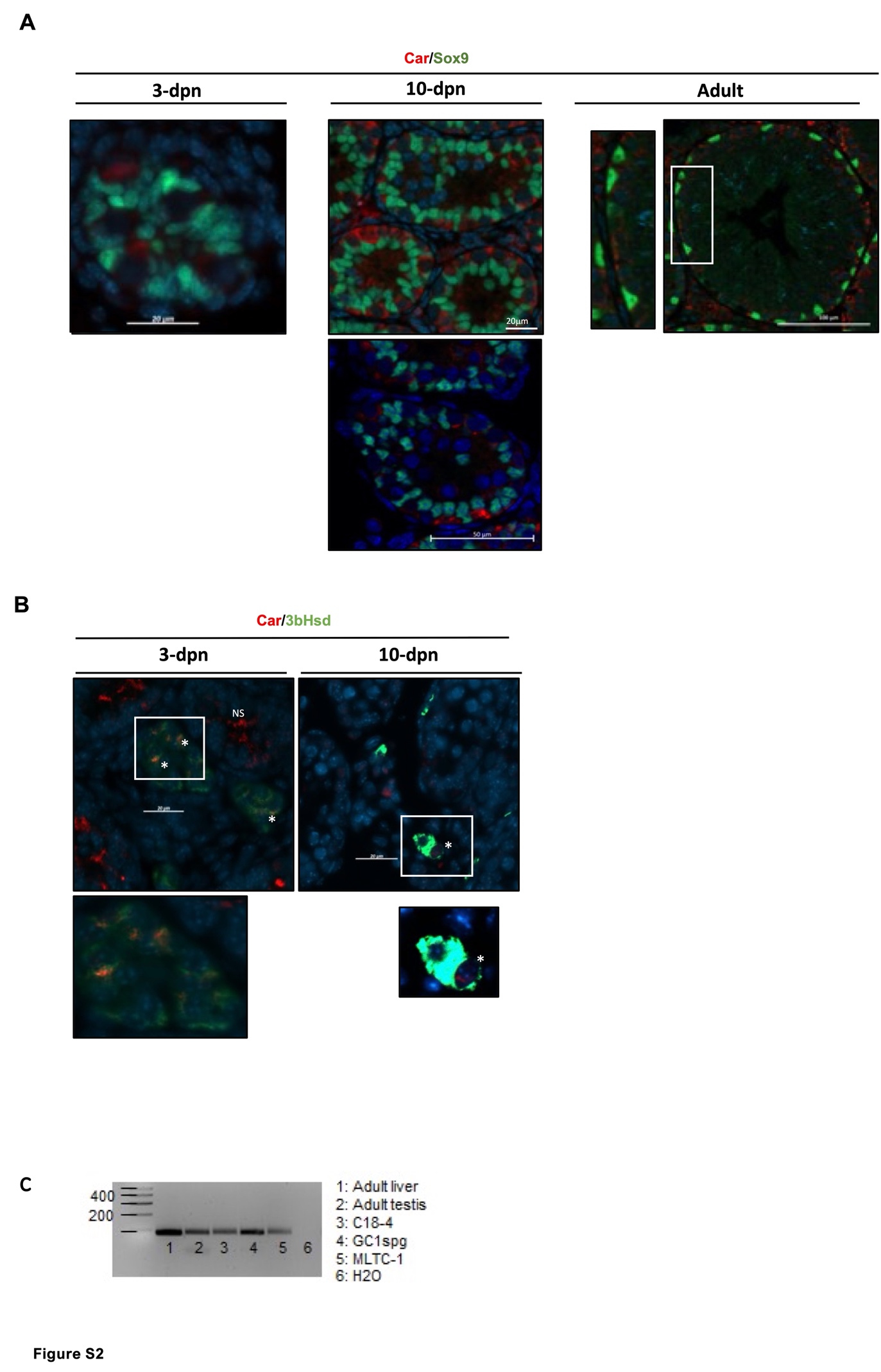
**

**Figure S2**

**A)** Representative testis micrographs of 3-dpn, 10-dpn, and adult (8 months) Wt males co-stained for Sox9 (Green) and Car (Red). White square indicates the enlarged area. The experiment was performed on three different male mice.

**B)** Representative testis micrographs of 3-dpn and 10-dpn Wt males co-stained for Hsd3b1 (Green) and Car (Red). White Stars indicate the co-stained cells. and white square indicates the enlarged area. The experiment was performed on three different male mice.

**C)** Representative image of PCR for mouse *Car* mRNA in adult liver, adult mouse testes, and in different cell lines C18-4 (Spermatogonia-A), GC1spg (spermatogonia B), and MLTC1 (Leydig).

**Figure S3**

Quantification of the number of Id4-positive cells per seminiferous tubule in 10-dpn testis of Wt or Car-/- males treated with vehicle or IA. The blue line indicates the median of each group. n=5 per group. Numbers were normalized to vehicle-treated Wt, which was arbitrarily set at 1.

**
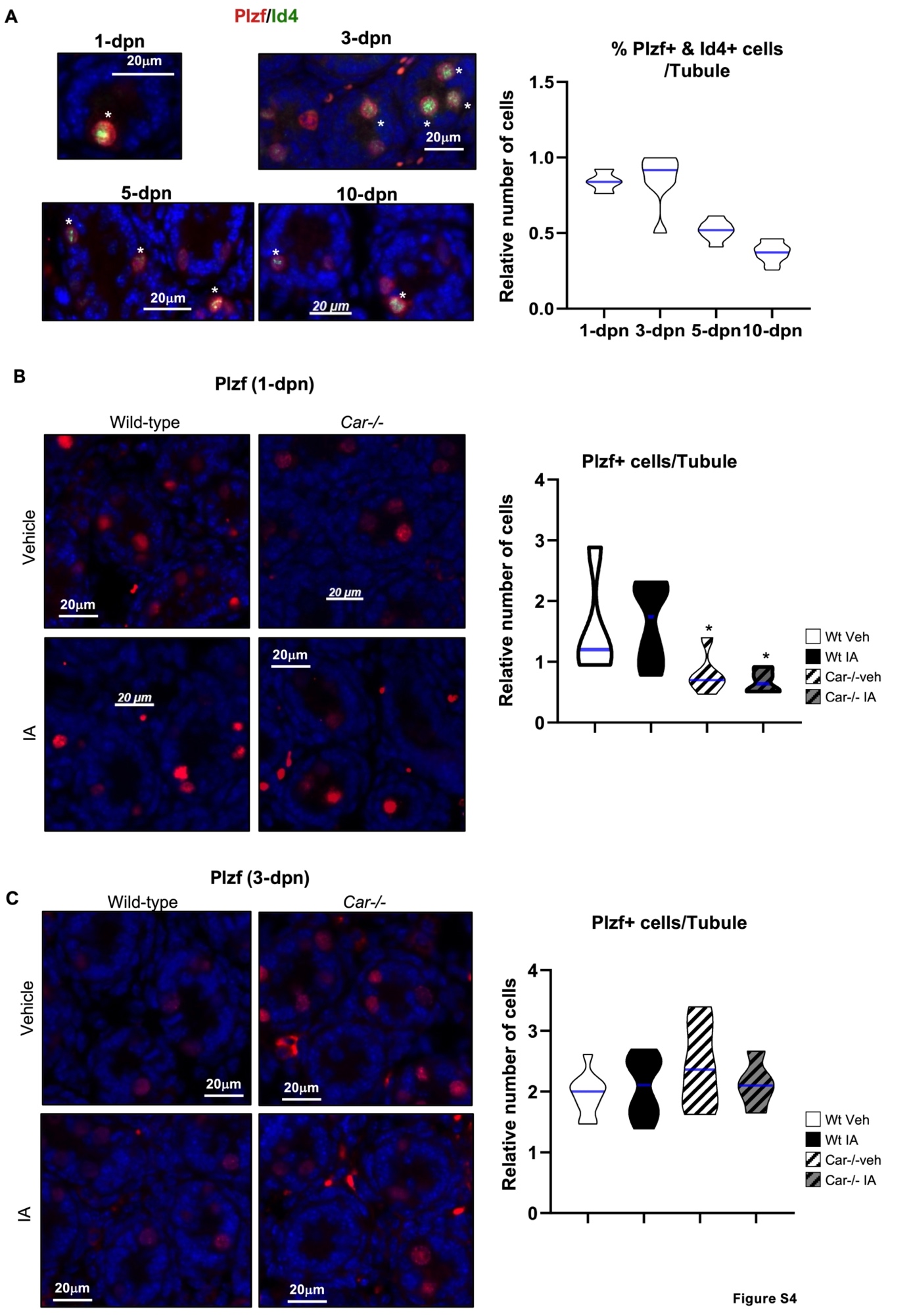
**

**Figure S4**

**A)** Representative testis micrographs of 1-dpn, 3-dpn, 5-dpn, and 10-dpn Wt males co-stained for Plzf (red) and Id4 (green). White stars indicate the co-stained cells. The experiment was performed on five different male mice. (Right) Quantification of the percentage of Plzf+ cells co-stained with Id4. The blue line indicates the median of each group.

**B)** (Left) Representative micrographs of the vehicle or IA-treated Wt and Car^-/-^ males testis stained for Plzf at 1 dpn; and (Right) quantification of the number of PLZF positive cells per seminiferous tubule in testes of Wt or Car^-/-^ males treated with vehicle or IA. The blue line indicates the median of each group. n=6 per group. Numbers were normalized to vehicle-treated Wt group, which was arbitrarily set at 1.

**C)** (Left) Representative micrographs of the vehicle or IA-treated Wt and Car^-/-^ males testis stained for Plzf at 3 dpn; and (Right) quantification of the number of Plzf positive cells per seminiferous tubule in testes of Wt or Car^-/-^ males treated with vehicle or IA. The blue line indicates the median of each group. n=6 per group. Numbers were normalized to vehicle-treated Wt group, which was arbitrarily set at 1.

Two-way ANOVA followed by Holm–Sidak’s test for multiple comparisons. *, p<0.05 and **, p < 0.01 versus Wt vehicle-treated group.

**
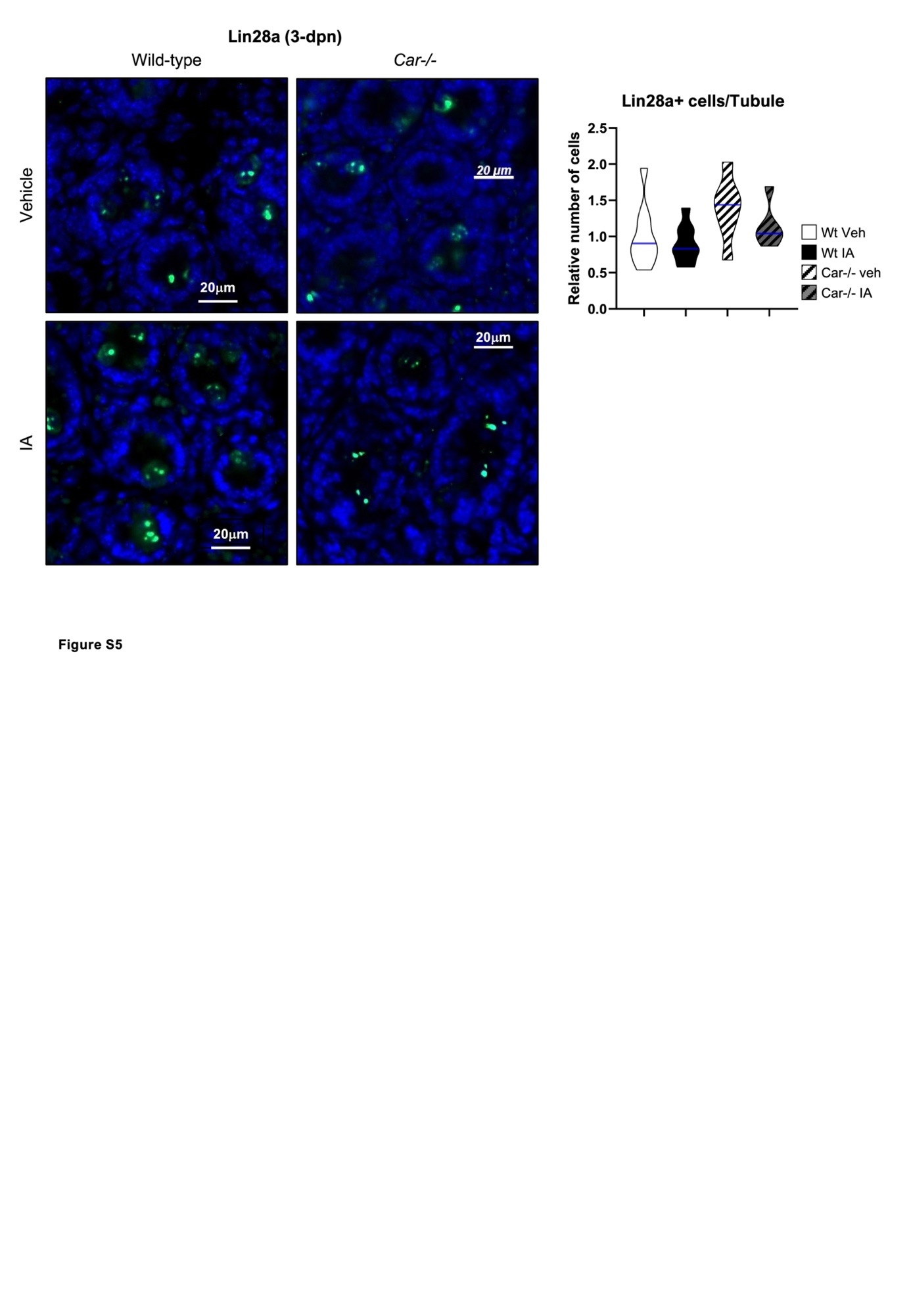
**

**Figure S5**

(Left panel) Representative micrographs of vehicle or IA-treated Wt and Car^-/-^ males testis stained for Lin28a at 3 dpn; and (Right panel) quantification of the number of Lin28a positive cells per seminiferous tubule in testis of Wt or Car^-/-^ males treated with vehicle or IA. The blue line indicates the median of each group. n=6 per group. Numbers were normalized to vehicle-treated Wt group, which was arbitrarily set at 1.

**
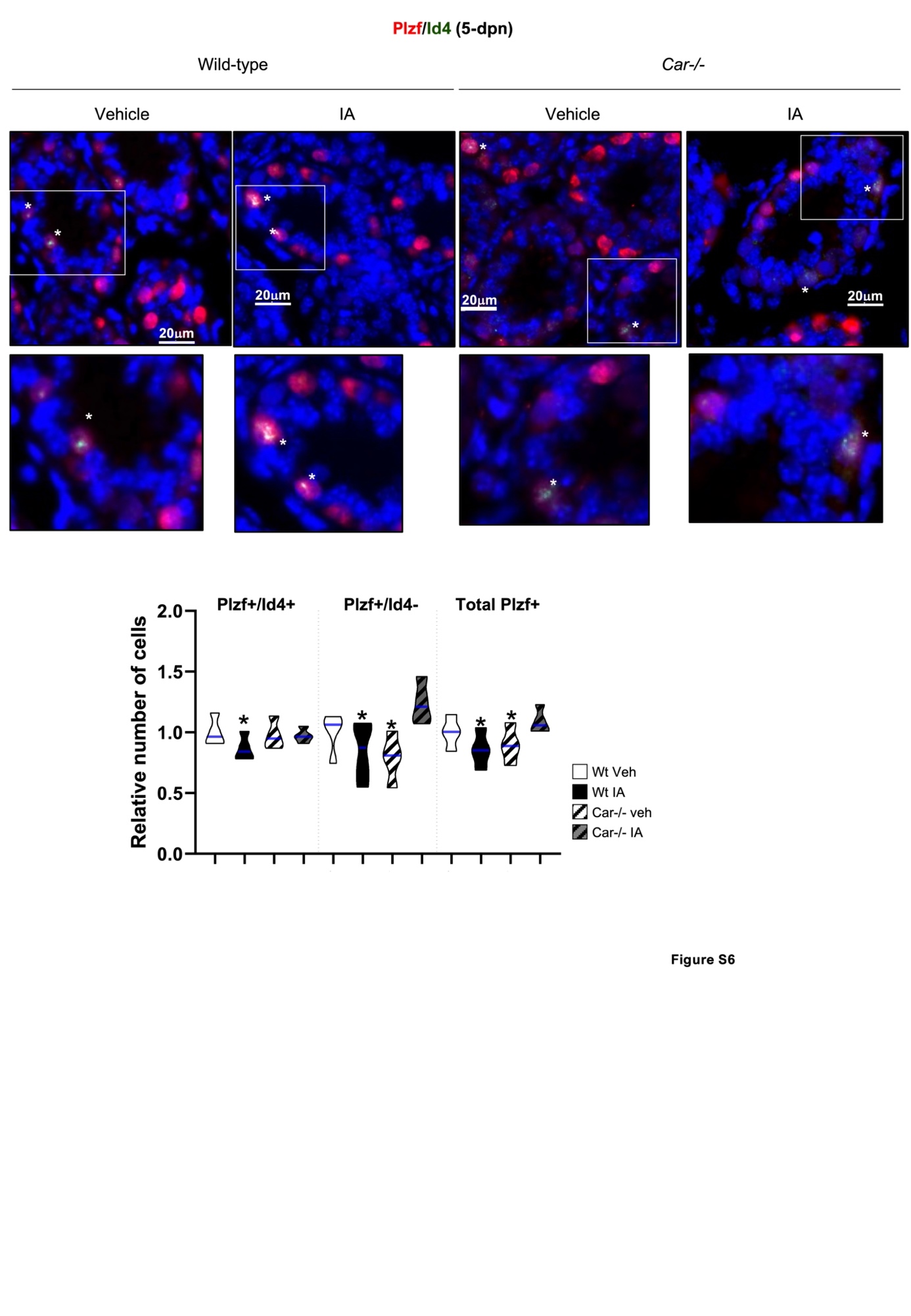
**

**Figure S6**

(Top panel) Representative testis micrographs of 5-dpn testis of males, co-stained for Plzf (red) and Id4 (green) in Wt or Car^-/-^ males treated with vehicle or IA. Stars indicate stained cells, while squares indicate the enlarged area.

(Bottom panel) Quantification of the relative number of Id4+/Plzf+ cells, of the Id4-/Plzf+ cells, and Plzf+ cells. Numbers were normalized to vehicle-treated Wt group, which was arbitrarily set at 1. The blue line indicates the median of each group. n=10 per group.

Two-way ANOVA followed by Holm–Sidak's test for multiple comparisons. *, p<0.05 and **, p < 0.01 vs. Wt vehicle-treated group.

**
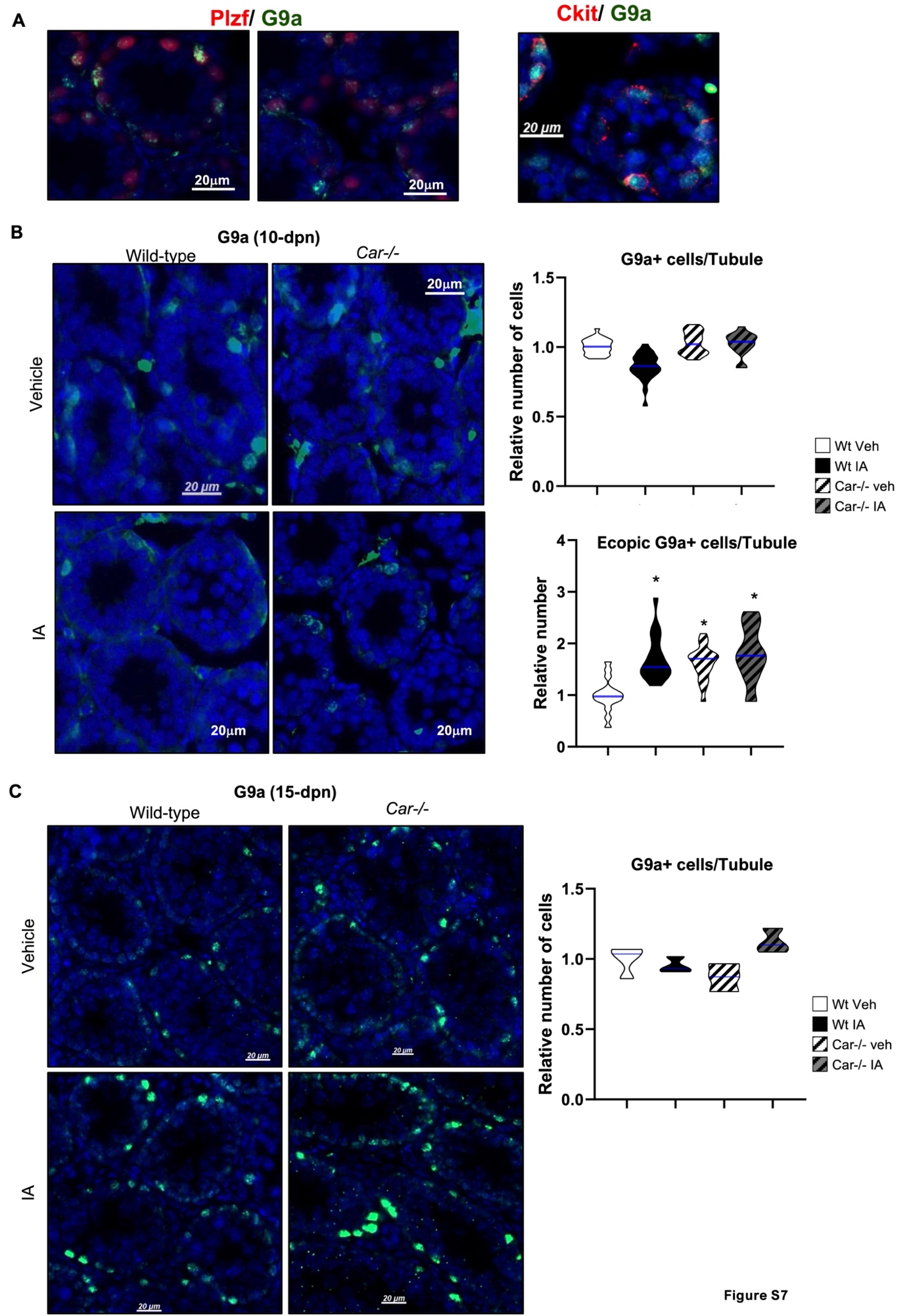
**

**Figure S7**

**A)** (Left) Representative testis micrographs of 10-dpn testis of Wt males co-stained for G9a (green) and a marker of undifferentiated spermatogonia, namely Plzf (red), was performed to define colocalization on germ cells. (Right) Representative testis micrographs of 10-dpn testes of WT males co-stained for G9a (green) and differentiating/differentiated spermatogonia, namely Ckit (red), were performed to define colocalization on germ cells.

**B)** (Left panel) Representative testis micrographs of 10-d-old vehicle or IA-treated Wt and Car^-/-^ males stained for G9a. (Right panel) Quantification of the number of G9a-positive cells per seminiferous tubule in testes of Wt or Car^-/-^ males treated with vehicle or IA. The blue line indicates the median of each group. n=20 per group for Wt and n=15 per group for Car^-/-^ males. Numbers were normalized to vehicle-treated Wt, which was arbitrarily set at 1.

**C)** (Left panel) Representative testis micrographs of 15-d-old vehicle or IA-treated Wt and Car^-/-^ males stained for G9a. (Right panel) Quantification of the number of G9a-positive cells per seminiferous tubule in testes of Wt or Car^-/-^ males treated with vehicle or IA. The blue line indicates the median of each group. n=6 per group. Numbers were normalized to vehicle-treated Wt which was arbitrarily set at 1.

Two-way ANOVA followed by Holm–Sidak's test for multiple comparisons. *, p<0.05 vs. Wt vehicle-treated group.

**Figure S8**

**A)** Quantification of proliferation revealed by the analysis of the relative number of Plzf/Pcna co-stained positive cells in Wt and Car^-/-^ 5 and 10-dpn testes treated with vehicle or IA. The blue line indicates the median of each group. n=20 per group for Wt and n=15 per group for Car^-/-^ males. Vehicle-treated cells were arbitrarily set at 1.

**B**) Quantification of apoptotic process, as revealed by the analysis of the relative number of TUNEL positive cells in Wt and Car^-/-^ 5 and 10-dpn testis treated with vehicle or IA. The blue line indicates the median of each group.

At 5 dpn: n=6 per group for Wt and n=6 per group for Car^-/-^ males. Vehicle-treated cells were arbitrarily set at 1. At 10 dpn, n=30 per group for Wt and n=20 per group for Car^-/-^ males. Vehicle-treated cells were arbitrarily set at 1.

Two-way ANOVA followed by Holm–Sidak's test for multiple comparisons. *, p < 0.05 Wt vehicle-treated male group. Veh: vehicle and IA: Inverse agonist

**
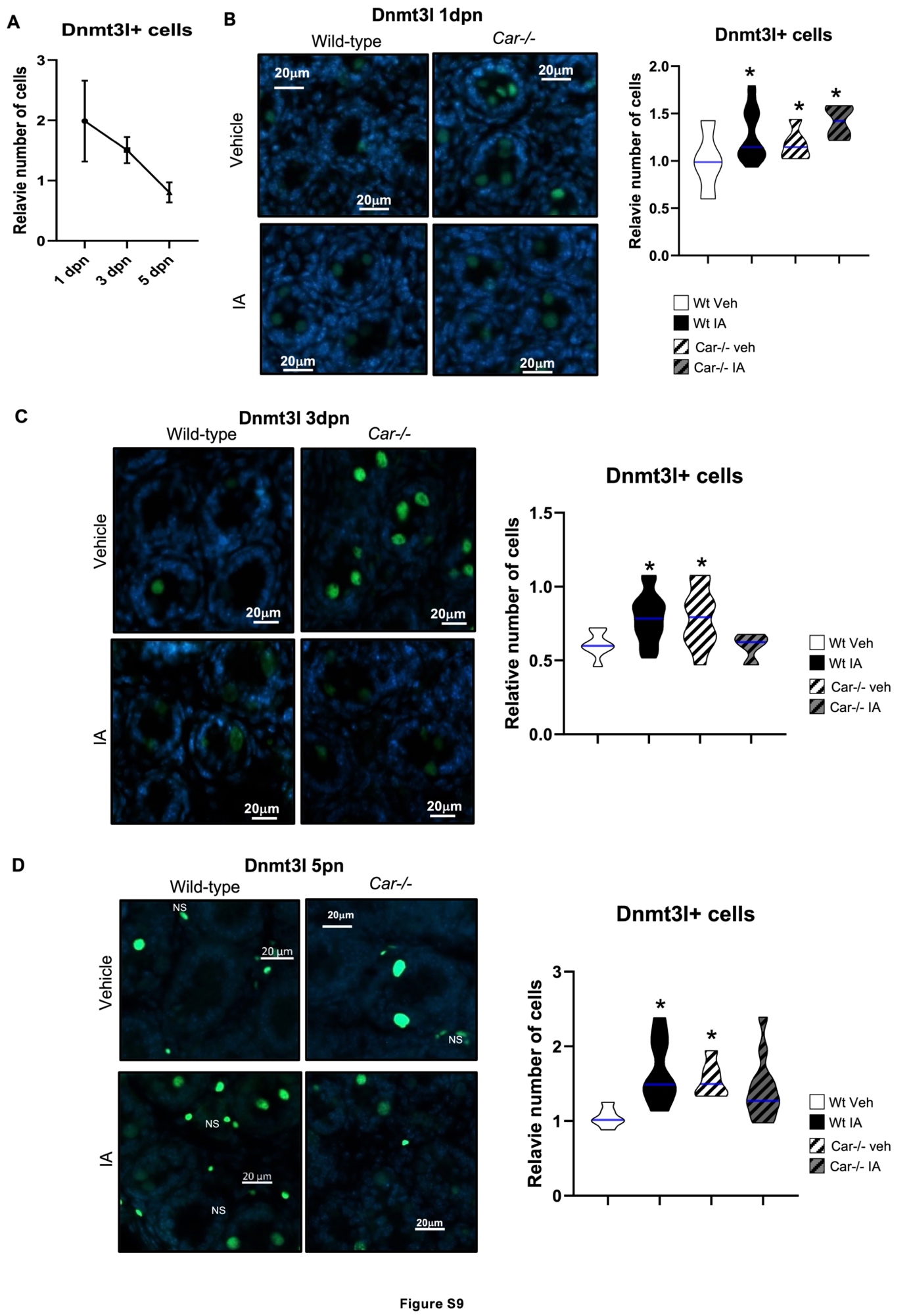
**

**Figure S9**

**A)** Quantification of the number of Dnmt3l-positive cells per seminiferous tubule in testes of Wt mice from 1 to 5pn. n=5 per group.

**B)** (Left panel) Representative micrographs of the vehicle or IA-treated Wt and Car^-/-^ males testis stained for Dnmt3l at 1 dpn; and (Right panel) quantification of the number of Dnmt3l-positive cells per seminiferous tubule in testes of Wt or Car^-/-^ males treated with vehicle or IA. The blue line indicates the median of each group. n=6 per group. Numbers were normalized to vehicle-treated Wt group, which was arbitrarily set at 1.

**C)** (Left panel) Representative micrographs of vehicle or IA-treated Wt and Car^-/-^ males testis stained for Dnmt3l at 3 dpn; and (Right panel) quantification of the number of Dnmt3l positive cells per seminiferous tubule in testis of WT or Car^-/-^ males treated with vehicle or IA. The blue line indicates the median of each group. n=6 per group. Numbers were normalized to vehicle-treated Wt group, which was arbitrarily set at 1.

**D)** (Left panel) Representative micrographs of the vehicle or IA-treated Wt and Car^-/-^ males testis stained for Dnmt3l at 5 dpn; and (Right panel) quantification of the number of Dnmt3l-positive cells per seminiferous tubule in testes of Wt or Car-/- males treated with vehicle or IA. The blue line indicates the median of each group. n=6 per group. Numbers were normalized to vehicle-treated Wt group, which was arbitrarily set at 1.

Two-way ANOVA followed by Holm–Sidak's test for multiple comparisons. *, p<0.05 vs. Wt vehicle-treated group.

**
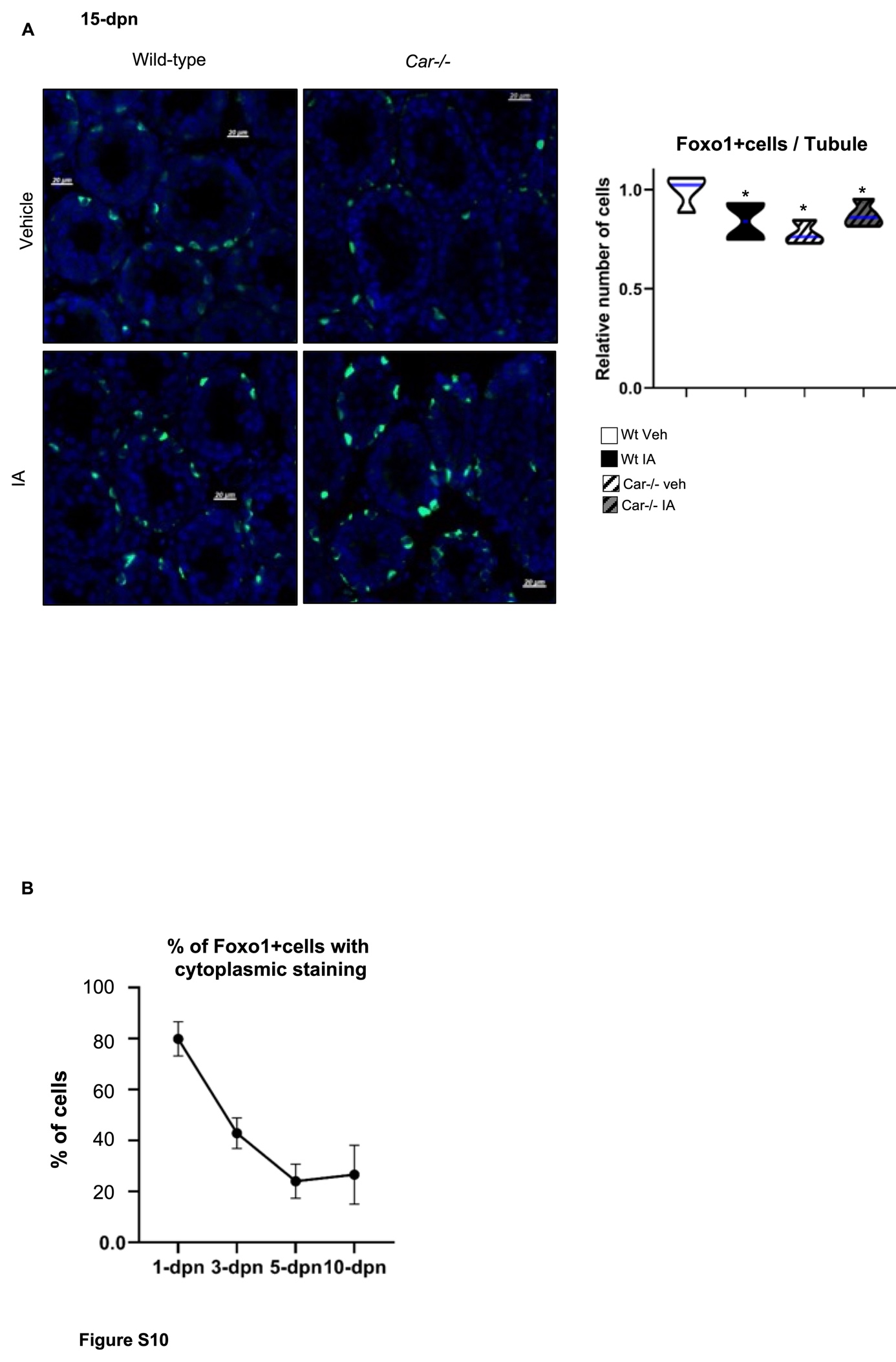
**

**Figure S10**

**A)** (Left panel) Representative micrographs of the 15-d-old vehicle or IA-treated Wt and Car^-/-^ testes stained for Foxo1. (Right panel) Quantification of the number of Foxo1-positive cells per seminiferous tubule in testes of Wt or Car^-/-^ males treated with vehicle or IA. The blue line indicates the median of each group. n=5 per group. Numbers were normalized to vehicle-treated Wt, which was arbitrarily set at 1. Two-way ANOVA followed by Holm–Sidak’s test for multiple comparisons. *, p<0.05 vs. Wt vehicle-treated group.

**B)** Quantification of the percentage of Foxo1-positive cells with cytoplasmic staining per seminiferous tubule in testes of Wt from 1 to 10 dpn. n=6 per group.

**
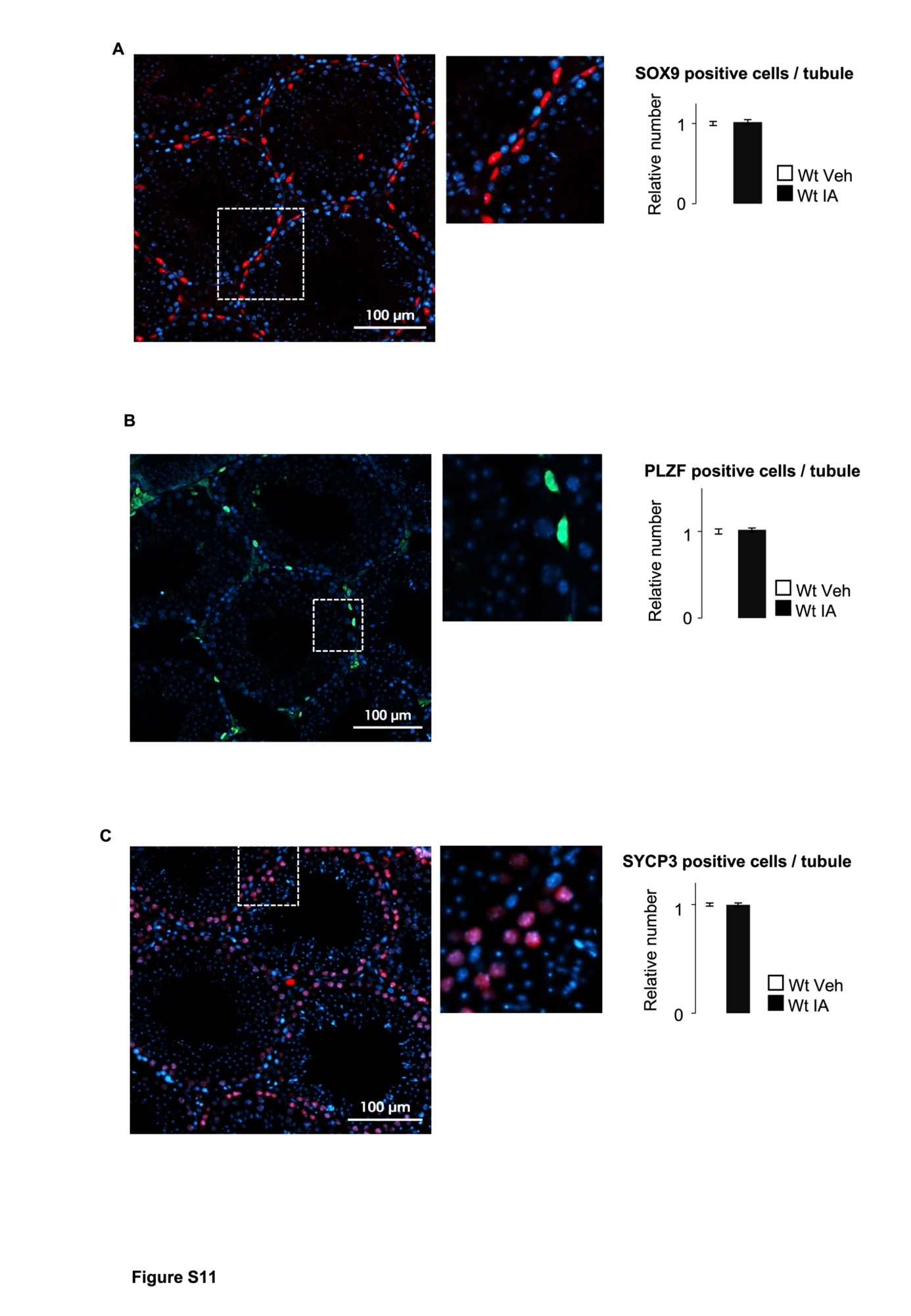
**

**Figure S11**

**A)** Representative micrograph of adult (8-month-old) IA-treated Wt testis stained for Sox9. Quantification of the number of Sox9-positive cells per seminiferous tubule in testes of Wt males treated with vehicle or IA. Numbers were normalized to vehicle-treated Wt group, which was arbitrarily set at 1. White square indicates the enlarged area.

**B)** Representative micrograph of adult (8-month-old) IA-treated Wt testes stained for Plzf. Quantification of the number of Plzf-positive cells per seminiferous tubule in testes of Wt males treated with vehicle or IA. Numbers were normalized to vehicle-treated Wt group, which was arbitrarily set at 1. White square indicates the enlarged area.

**C)** Representative micrograph of adult (8-month-old) IA-treated Wt testes stained for Sycp3. Quantification of the number of Sycp3-positive cells per seminiferous tubule in testes of Wt males treated with vehicle or IA. Numbers were normalized to vehicle-treated Wt group, which was arbitrarily set at 1. White square indicates the enlarged area.

In the panel, n=26 for Wt veh; n=24 for IA from five independent experiments. A T-test was performed to compare the two groups.

**Figure S12.**

**A)** Relative number of sperm count in the epididymis head of Wt or Car^-/-^ males; Wt males were arbitrarily set at 1.

**B)** Relative number of sperm count in the epididymis head of Wt or Car^-/-^ males treated neonatally with vehicle or IA, Wt vehicle-treated males were arbitrarily set at 1.

**Figure S13.**

No statistical differences were observed among conditions regarding fine motility parameters including **(A)** curvilinear velocity or VCL, (**B)** straight-line velocity or VSL, and **(C)** lateral amplitude of the head or ALH. Statistical significance was assessed using an unpaired Student t-test. Each group was compared individually with all other groups one by one. For each histogram, plots sharing different small letters represent statistically significant differences between the groups (p < 0.05), and plots with a common letter do not present statistically significant differences between the groups (p > 0.05). The corresponding statistical data can be found in supplementary Table 3.

**Figure S14.**

**A)** Percentage of pregnant C57BL/6J females following plugged (visualized by vaginal plug) after 14 d of reproduction with Wt or Car^-/-^ males neonatally treated with vehicle or IA. The number of male vehicle-treated Wt n=39 and IA-treated Wt n=43; Vehicle-treated Car^-/-^ n= 24 and IA-treated Car^-/-^ n=25 mice with two females per male. Two-way ANOVA followed by Holm–Sidak's test for multiple comparisons.

**B)** Number of embryos per litter visualized at E2.5 after vaginal plug obtained from the breeding of Wt or Car^-/-^ males neonatally treated with vehicle or IA with C57Bl6J females. Seven males per group were put into breeding with two C57BL/6J females. n=7 per group.

**Figure S15**

**A)** Schematic representation of the experimental design (primers) of Crispr/CAS9 experiment for Car deletion.

**B)** DNA alignment between wild-type Car sequences obtained following Crispr/CAS9 protocol.

**C)** Protein alignment between wild-type Car protein and the truncated protein obtained following Crispr/CAS9 protocol.

**D)** *Car* mRNA accumulation observed in Wt cells and CRISPR/Cas9 *Car* knockout cells as observed using RT-qPCR. Vehicle-treated Wt cells were arbitrarily set at 1.

**
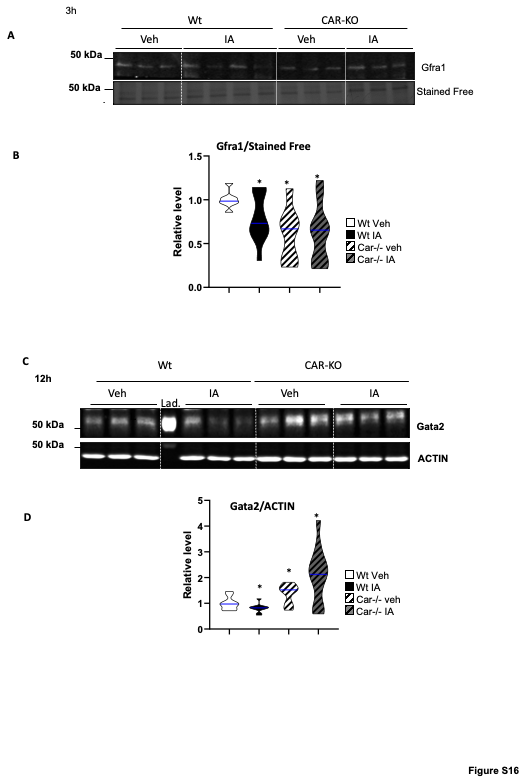
**

**Figure S16.**

**A**) Representative western blots of Gfra1 in Wt and Car-KO cells treated with vehicle for 3 h.

**B**) Quantification of Gfra1 protein accumulation in Wt and Car-KO cells treated with vehicle or IA for 3 h. Normalization was performed relative to total protein using unstained gels. The Wt vehicle group was arbitrarily set at 1.

**C)** Representative western blots of Gata2 in Wt and Car-KO cells treated with vehicle or IA for 24 h.

**D**) Quantification of Gata2 protein accumulation in Wt and Car-KO cells treated with vehicle or IA for 24 h. Normalization was performed relative to total protein using unstained gels. The Wt vehicle group was arbitrarily set at 1. (Lad.: Ladder).

In all panels n=8 per group. Two-way ANOVA followed by Holm‐Sidak's test for multiple comparisons. *, p < 0.05 Wt vehicle-treated male group. Veh: vehicle and IA: Inverse agonist.

**
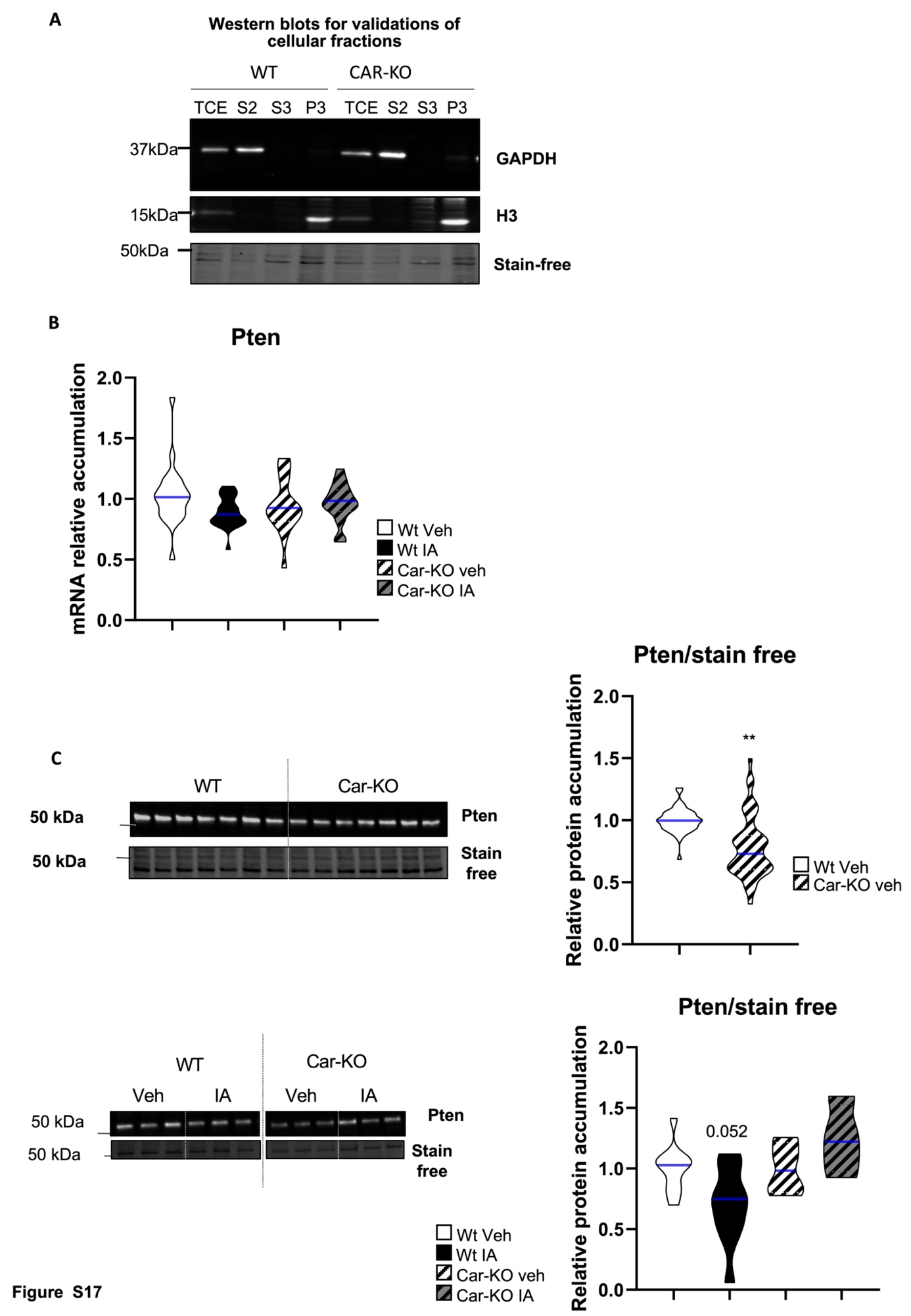
**

**Figure S17.**

**A)** Representative images of the validation of the fraction experiments by western blots in Wt and CAR-Ko cells using GAPDH (cytoplasmic maker) or Histone H3 (nuclear marker). TCE; Whole cell extract; S2: Cytoplasm; S3 nucleoplasm; P3: Chromatin.

**B)** Relative mRNA accumulations of *Pten* normalized to β‐actin on Wt and Car-KO cells treated with vehicle or IA. The blue line indicates the median for each group. Vehicle-treated groups of each genotype were arbitrarily set at 1. Analyses were performed at 3 h after treatment.

**C)** Representative western blots of Pten in Wt and CAR-KO cells were treated with a vehicle for 3 h. Quantification of Pten protein accumulation in Wt and Car-KO cells treated with vehicle or IA for 3 h. Normalization was performed relative to total protein using unstained gels. For vehicle Wt and Car-KO cells analysis, Wt vehicle group was arbitrarily set at 1. For the analysis of IA impact, vehicle group of each genotype were arbitrarily set at 1.

In B and C panels, n=8 per group. Two-way ANOVA followed by Holm–Sidak's test for multiple comparisons. *, p < 0.05 Wt vehicle-treated male group. Veh: vehicle and IA: Inverse agonist.

**Figure S18.**

Quantification of the relative number of TUNEL-positive cells in Wt and Car-KO cells treated with vehicle or IA for 24 h. Vehicle-treated cells were arbitrarily set at 1.

**
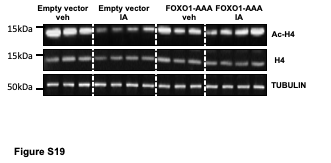
**

**Figure S19.**

Representative western blots of H4 and Ac-H4 in Wt and Car-KO cells transfected with an empty vector or a vector for overexpression of none phosphorylable Foxo1 (Foxo1-AAA) and treated with vehicle or IA for 24 h.


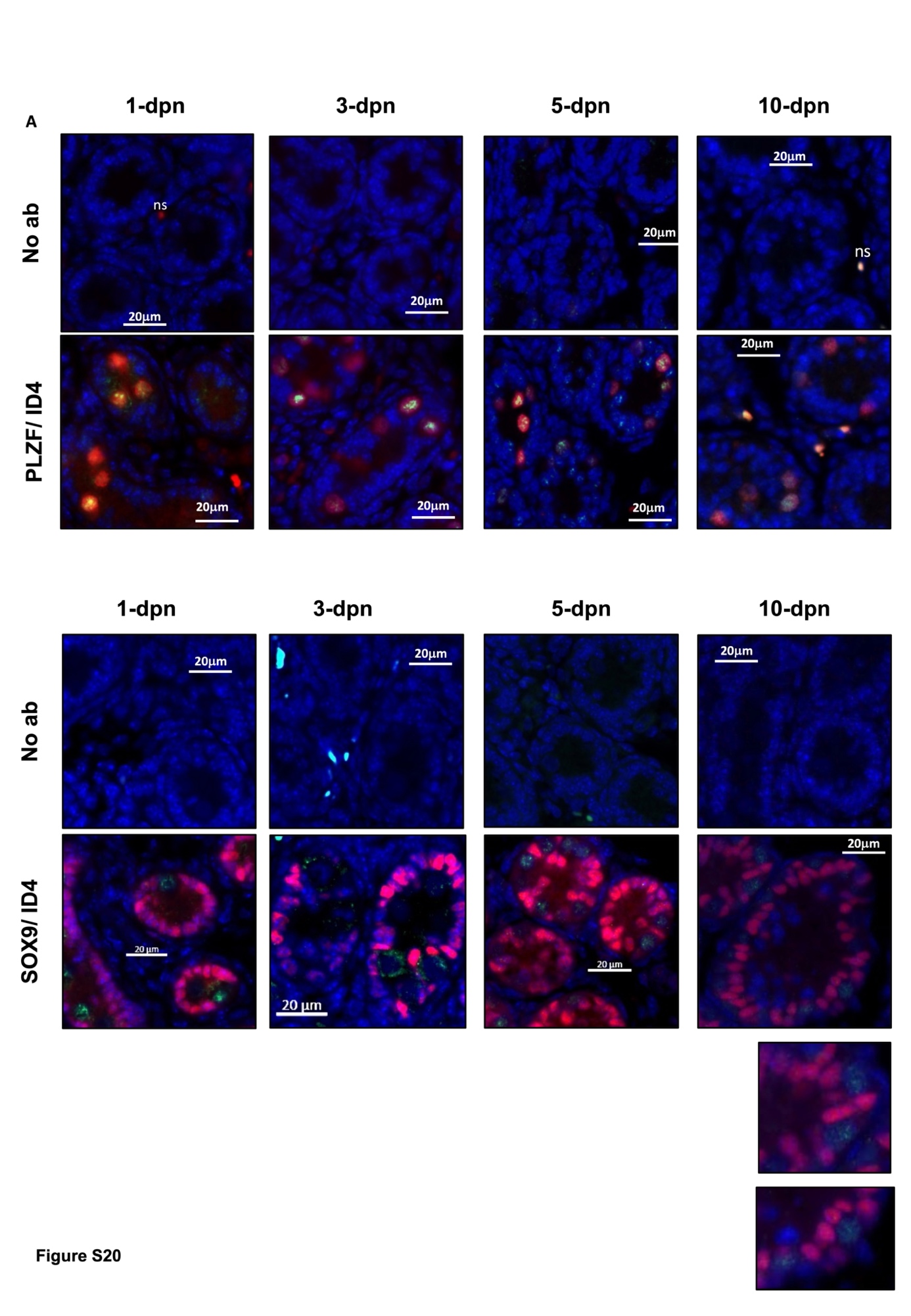


**Figure S20. Validation of Id4 immunostaining**

The validation of the Id4 antibody for immunostaining was performed using slides 1-dpn, 3-dpn, 5-dpn, and 10-dpn Wt testis. For that:

**A)** (Top line) Immunostaining without primary antibody was performed as negative control; (Bottom line) Co-staining of Id4 with another marker of spermatogonia, namely Plzf, was performed to define colocalization in germ cells. (ns: non-specific).

**B**) (Top line) Immunostaining without primary antibody was performed as negative control; (Bottom line) Co-immunostaining of Id4 with the Sertoli cell marker, namely Sox9, was performed to demonstrate the lack of overlap Id4 staining with the Sertoli cell marker. The white squares indicate the enlarged region.


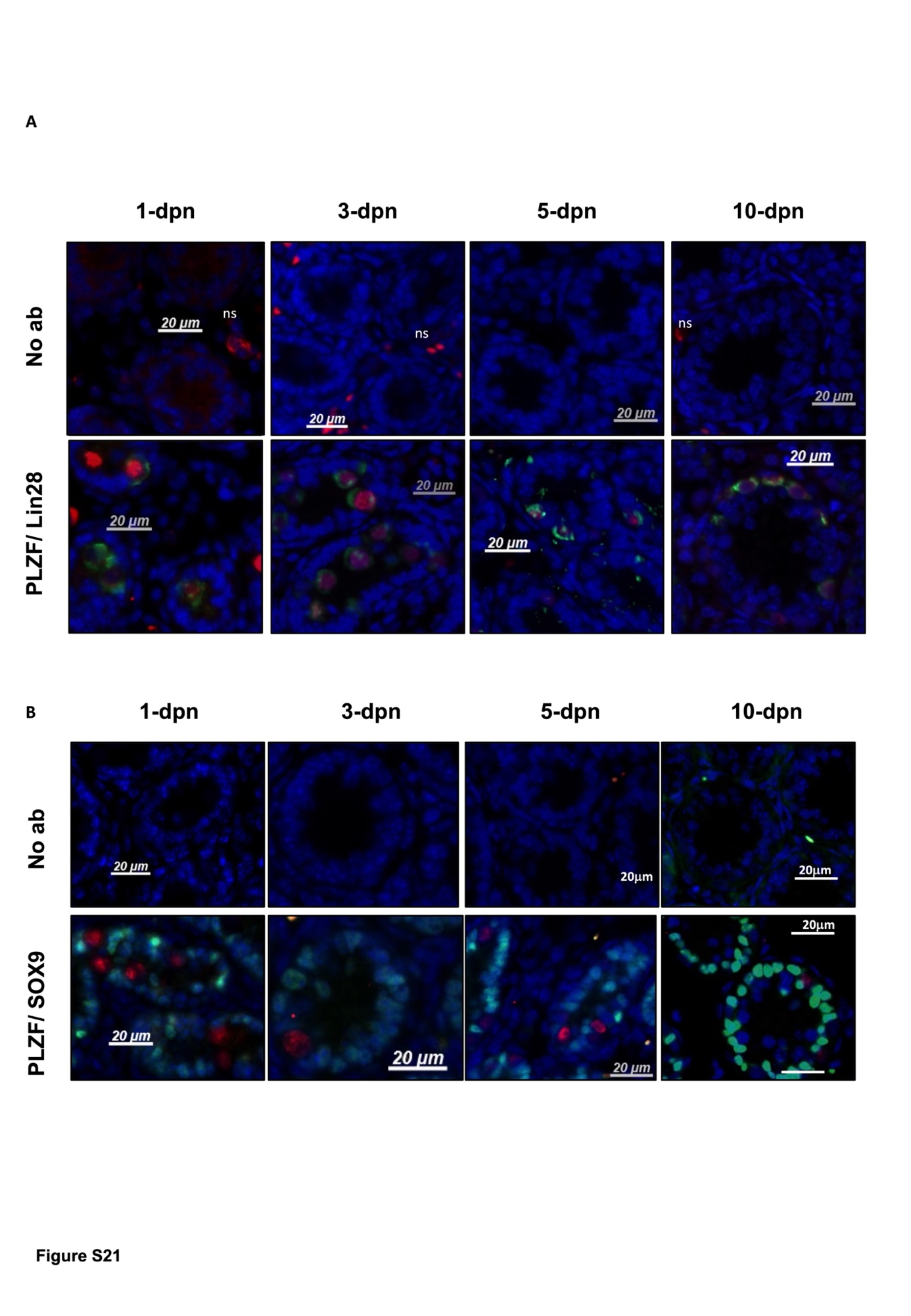


**Figure S21. Validation of Plzf immunostaining**

Next to the co-staining with Id4 (Supplemental 20), the validation of the Plzf antibody for immunostaining was performed using slides 1-dpn, 3-dpn, 5-dpn, and 10-dpn Wt testis. For that:

**A)** (Top line) Immunostaining without primary antibody was performed as negative control; (Bottom line) Co-staining of Plzf with another marker of spermatogonia, namely Lin28a, was performed to define colocalization in germ cells.

**B)** (Top line) Immunostaining without primary antibody was performed as negative control; (Bottom line) Co-staining of Plzf with a marker of Sertoli cells, namely Sox9, was performed to demonstrate the lack of overlap Plzf staining with the Sertoli cell marker.

**
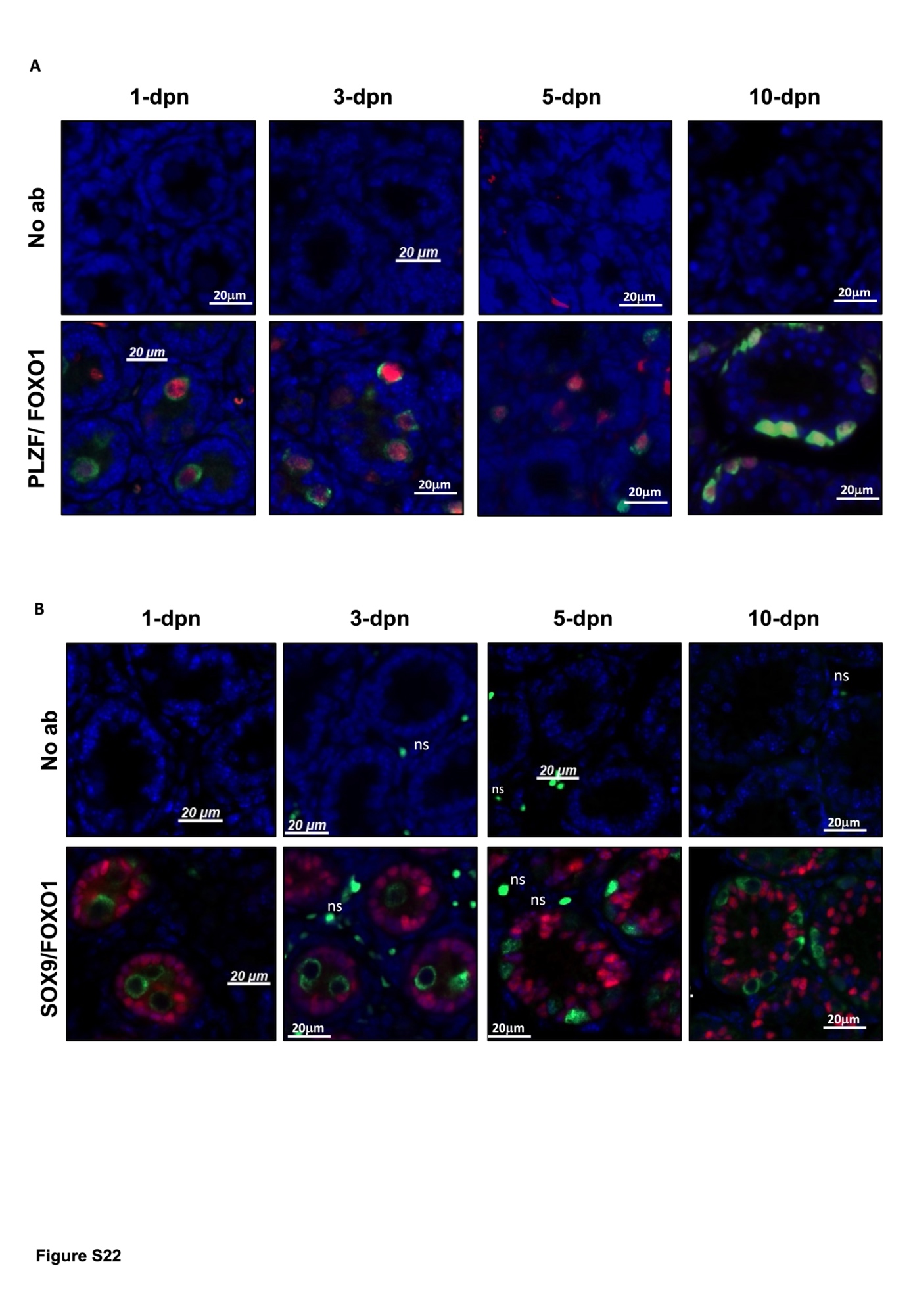
**

**Figure S22**. Validation of Foxo1 immunostaining

The validation of the Foxo1 antibody for immunostaining was performed using slides 1-dpn, 3-dpn, 5-dpn, and 10-dpn WT testis. For that:

**A)** (Top line) Immunostaining without primary antibody was performed as negative control; (Bottom line) Co-staining of Foxo1 with a marker of spermatogonia, namely Plzf, was performed to define colocalization in germ cells.

**B)** (Bottom line) Immunostaining without primary antibody was performed as negative control; (Bottom line) Co-staining of Foxo1 to a marker of Sertoli cell, namely Sox9, was performed to demonstrate the lack of overlap of Foxo1 staining with the Sertoli cell marker.

**
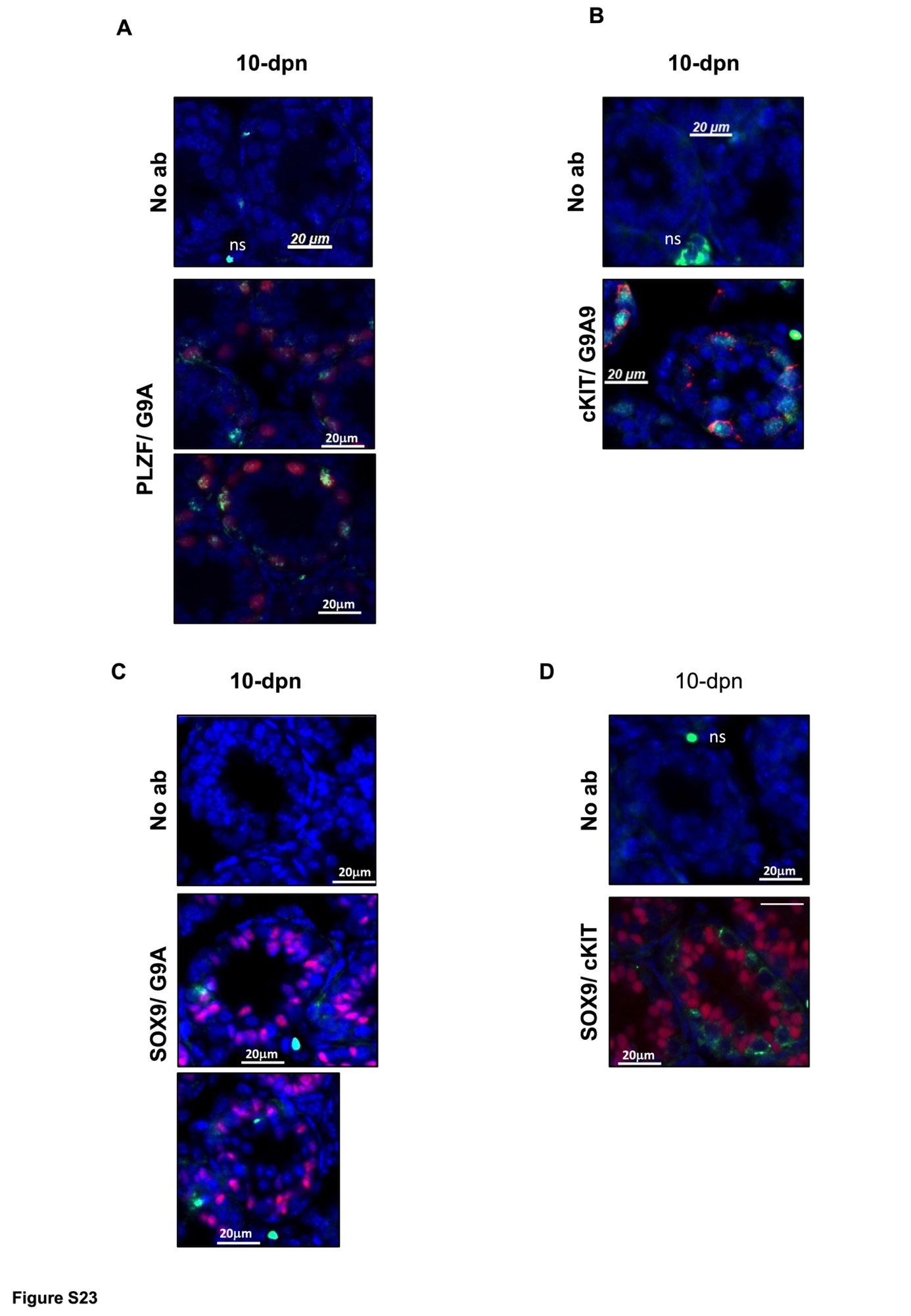
**

**Figure S23. Validation of G9a and cKit immunostaining**

The validation of the G9a antibody for immuno-staining was performed using a slide of 10-dpn Wt testis. For that:

**A)** (Top left) Immunostaining without primary antibody was performed as a negative control; (Middle and Bottom left) Co-staining of G9a with a spermatogonial marker, namely Plzf, was performed to define colocalization in germ cells.

**B)** (Top right) Immunostaining without primary antibody was performed as a negative control; (Bottom right) Co-staining of G9a with a marker of differentiated spermatogonia, namely cKit, was performed to define colocalization in germ cells.

**C)** (Top Left) Immunostaining without primary antibody was performed as negative control; (Middle and Bottom Left) Co-staining of G9a with a marker of Sertoli cells, namely Sox9, was performed to define the lack of colocalization in Sertoli cells.

**D)** (Top right) Immunostaining without primary antibody was performed as negative control; (Bottom right) Co-staining of cKit with a marker of Sertoli cells, namely Sox9, was performed to define the lack of colocalization in Sertoli cells.

**
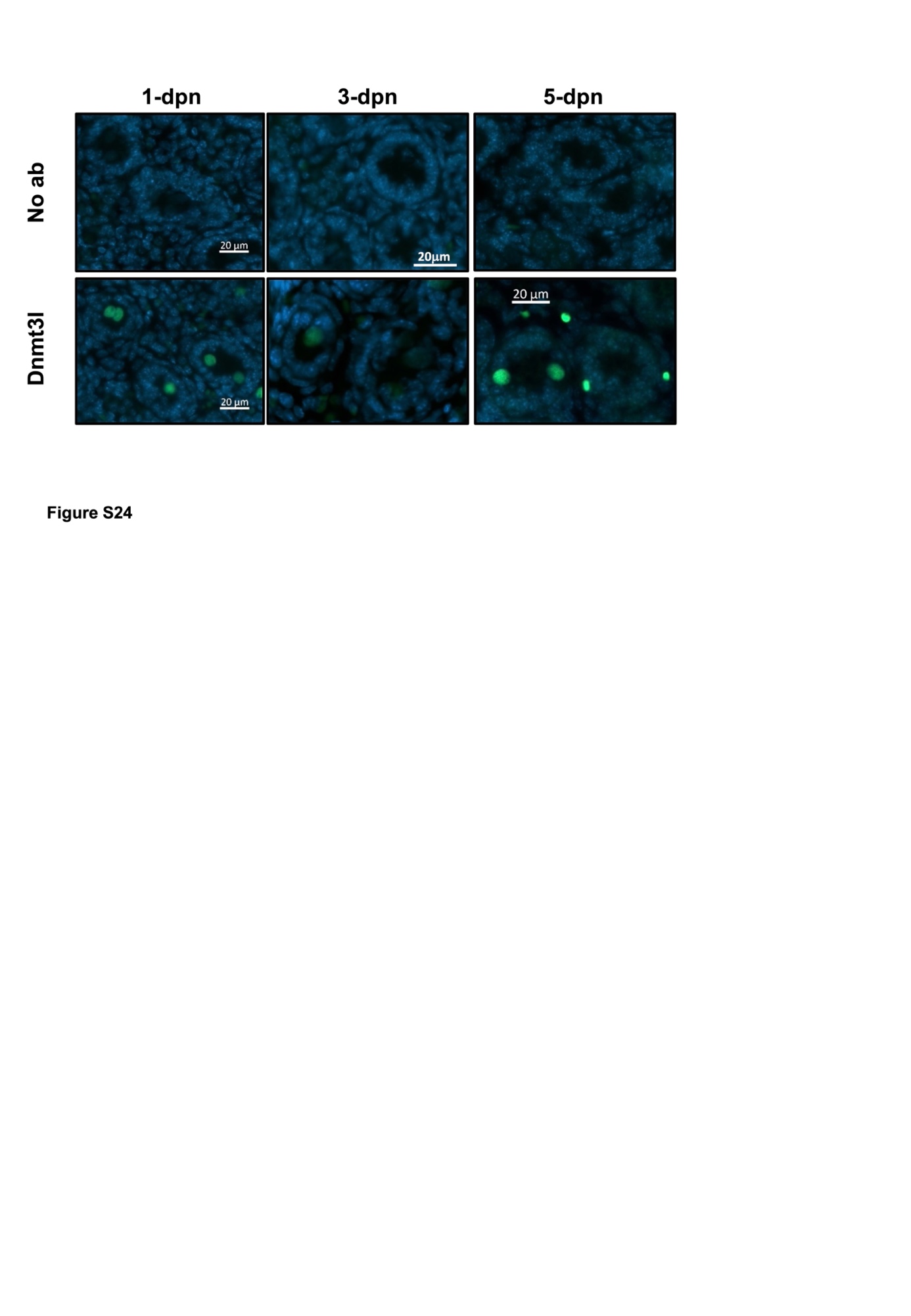
**

**Figure S24. Validation of Dnmt3l immuno-staining**

(Top line) Immunostaining without primary antibody was performed as negative control; (Bottom line) Immunostaining of Dnmt3l on Wt testis treated with veh at 1 dpn, 3 dpn, and 5 dpn.

**Supplemental Tables**

**Table S1.** Statistical data associated with the Student *t*-test performed in Figure 11. DoF = Degrees of Freedom; CI = Confidence Interval.

|  | **Concentration** | | | | **Motility** | | | |
| --- | --- | --- | --- | --- | --- | --- | --- | --- |
| **Groups** | **t-value** | **DoF** | **p-value** | **95% CI** | **t-value** | **DoF** | **p-value** | **95% CI** |
| Wt veh versus Wt IA | 2.701 | 8 | 0.02703 | 0.7107302 / 9.0092698 | -0.373 | 8 | 0.7188 | -15.08299 / 10.88299 |
| Wt veh versus *Car*-/- veh | 4.1803 | 8 | 0.003078 | 3.802164 / 13.157836 | 0.67641 | 8 | 0.5179 | -11.22681 / 20.54681 |
| Wt veh versus *Car*-/- IA | 4.065 | 8 | 0.003608 | 3.600178 / 13.039822 | -0.0052 | 8 | 0.9959 | -8.815041 / 8.775041 |
| Wt IAversus *Car*-/- veh | 1.9362 | 8 | 0.08886 | -0.6913028 / 7.9313028 | 0.89178 | 8 | 0.3985 | -10.72033 / 24.24033 |
| Wt ai versus *Car*-/- IA | 1.8313 | 8 | 0.1044 | -0.8968224 / 7.8168224 | 0.41983 | 8 | 0.6857 | -9.344709 / 13.504709 |
| *Car*-/- veh versus *Car*-/- IA | -0.0758 | 8 | 0.9414 | -5.022881 / 4.702881 | -0.7371 | 8 | 0.4821 | -19.320929 / 9.960929 |

**Table S2.** Statistical data associated with the Student *t*-test performed in Figure 12. DoF = Degrees of Freedom; CI = Confidence Interval.

|  | **Abnormal cells** | | | | **Head anomalies** | | | | **Flagellum anomalies** | | | |
| --- | --- | --- | --- | --- | --- | --- | --- | --- | --- | --- | --- | --- |
| **Groups** | **t-value** | **DoF** | **p-value** | **95% CI** | **t-value** | **DoF** | **p-value** | **95% CI** | **t-value** | **DoF** | **p-value** | **95% CI** |
| Wt veh versus Wt IA | -7.0993 | 8 | 0.0001021 | -5.56425 / -2.83575 | -7.5427 | 8 | 6.654e-05 | -5.614625 / -2.985375 | 0.30861 | 8 | 0.7655 | -0.6472307 / 0.8472307 |
| Wt veh versus *Car*-/- veh | -18.769 | 8 | 6.708e-08 | -12.014625 / -9.385375 | -15.629 | 8 | 2.802e-07 | -12.164008 / -9.035992 | -0.31623 | 8 | 0.7599 | -0.8292225 / 0.6292225 |
| Wt veh versus *Car*-/- IA | -14.776 | 8 | 4.331e-07 | -12.138724 / -8.861276 | -15.354 | 8 | 3.215e-07 | -11.846915 /  -8.753085 | -0.49237 | 8 | 0.6357 | -1.1367033 / 0.7367033 |
| Wt IA versus *Car*-/- veh | -9.2387 | 8 | 1.528e-05 | -8.122418 / -4.877582 | -8.0335 | 8 | 4.238e-05 | -8.108413 / -4.491587 | -0.56569 | 8 | 0.5871 | -1.0152956 / 0.6152956 |
| Wt IAversus *Car*-/- IA | -7.6681 | 8 | 5.916e-05 | -8.194576 / -4.405424 | -7.7139 | 8 | 5.67e-05 | -7.79365 / -4.20635 | -0.68825 | 8 | 0.5108 | -1.3051639 / 0.7051639 |
| *Car*-/- veh versus *Car*-/- IA | 0.248 | 8 | 0.8103 | -1.65916 / 2.05916 | 0.34874 | 8 | 0.7363 | -1.6837 / 2.2837 | -0.2325 | 8 | 0.822 | -1.0918499 / 0.8918499 |

**Table S3.** Statistical data associated with the Student *t*-test performed in Supplemental Figure 13. DoF = Degrees of Freedom ; CI = Confidence Interval.

|  | **VCL** | | | | **ALH** | | | | **VSL** | | | |
| --- | --- | --- | --- | --- | --- | --- | --- | --- | --- | --- | --- | --- |
| **Groups** | **t-value** | **DoF** | **p-value** | **95% CI** | **t-value** | **DoF** | **p-value** | **95% CI** | **t-value** | **DoF** | **p-value** | **95% CI** |
| Wt veh versus Wt IA | -0.1745 | 8 | 0.8658 | -41.26754 / 35.45954 | 0.09219 | 8 | 0.9288 | -2.305081 / 2.497081 | -0.11931 | 8 | 0.908 | -24.67782 / 22.24982 |
| Wt veh versus *Car*-/- veh | 0.9589 | 8 | 0.3657 | -28.96426 / 70.20026 | 0.43621 | 8 | 0.6742 | -2.040357 / 2.992357 | 1.2262 | 8 | 0.255 | -20.38227 / 66.67427 |
| Wt veh versus *Car*-/- IA | -0.5639 | 8 | 0.5883 | -46.7397 / 28.3717 | -0.0824 | 8 | 0.9363 | -2.779981 / 2.587981 | -1.0579 | 8 | 0.321 | -33.30564 / 12.35764 |
| Wt IA versus *Car*-/- veh | 1.1188 | 8 | 0.2957 | -24.95974 / 72.00374 | 0.44779 | 8 | 0.6662 | -1.57691 / 2.33691 | 1.3665 | 8 | 0.209 | -16.7495 / 65.4695 |
| Wt IA versus *Car*-/- IA | -0.4012 | 8 | 0.6987 | -42.37029 / 29.81029 | -0.2042 | 8 | 0.8433 | -2.360225 / 1.976225 | -1.2002 | 8 | 0.264 | -27.052257 / 8.532257 |
| *Car*-/- veh versus *Car*-/- IA | -1.4364 | 8 | 0.1888 | -77.64704 / 18.04304 | -0.5746 | 8 | 0.5813 | -2.867226 / 1.723226 | -1.9024 | 8 | 0.093 | -74.371976 / 7.131976 |

Table S4: Antibodies used in western blot experiments

| **Antibody** | **Species** | **Supplier** | **Reference** |
| --- | --- | --- | --- |
| **P-Foxo1 (Ser256)** | Rabbit | Cell Signaling | 9461 |
| **Foxo1** | Rabbit | Cell Signaling | 2880s |
| **G9a** | Rabbit | Cell Signaling | C6H3 |
| **ac-H4** | Rabbit | Millipore | 06-946 |
| **H4** | Mouse | Cell Signaling | 2935s |
| **Tubulin** | Mouse | Sigma Aldrich | T5168 |
| **Akt** | Rabbit | Cell Signaling | 4685s |
| **P-Akt** | Rabbit | Cell Signaling | 4060s |
| **Gapdh** | Rabbit | Sigma Aldrich | G9545 |
| **H3** | Goat | Santa Cruz | sc865 |
| **Gfra1** | Mouse | Santa Cruz | sc271546 |
| **Gata2** | Rabbit | Abcam | ab109241 |
| **IgG Dylighconjugate 680** | Mouse | Cell Signaling | 5470p |
| **IgG Dylighconjugate 800** | Rabbit | Cell Signaling | 5151p |
| **IgG Dylighconjugate 680** | Goat | Santa cruz | Sc-516245 |
| **IgG Dylighconjugate 488** | Mouse | Bethyl | A90-317D2 |

Table S5: Antibodies used in immunohistochemistry experiments

|  | **Species** | **Distributors** | **References** | **Unmasking** | **Saturation** | **Dilution** | **Amplification** |
| --- | --- | --- | --- | --- | --- | --- | --- |
| **Hyper-Acetylated H4** | Rabbit | Millipore | 06-946 | Citrate-tween PH6 | BSA10% | 1/1000 | No |
| **G9a** | Rabbit | Cell Signaling | C6H3 | Citrate-tween PH6 | BSA10% | 1/500 | Yes |
| **Plzf** | Rabbit | Santa Cruz | sc-22839 | Citrate-tween PH6 | BSA10% | 1/200 | No |
| **Foxo1** | Rabbit | Cell Signaling | 2880s | Citrate-tween PH6 | BSA10% | 1/500 | Yes |
| **Pcna** | Mouse | Santa Cruz | sc-56 | Citrate-tween PH6 | BSA10% | 1/500 | No |
| **Id4** | Rabbit | Santa Cruz | sc491 | Citrate-tween PH6 | BSA10% | 1/500 | No |
| **cKit** | Rabbit | Cell Signaling | 3074p | Citrate-tween PH6 | BSA10% | 1/500 | No |
| **Dnmt3l** | Rabbit | Abcam | ab194094 | Tris EDTA PH9 | BSA10% | 1/250 | Amplification |
| **Lin28a** | Goat | R&D system | af3757 | Citrate-tween PH6 | BSA10% | 1/500 | Amplification |

Table S6: Sequences of RNA guides and primers used for CRISPR/CAS9 genotyping

| **Material for generation and validation of Car CRISPR/CAS9 cells** |
| --- |
| mCar_guide1: AGCCCTCACAAGTCAGGGCG |
| mCar_guide2: TAGTGTTAGCATAGCTGTCA |
| Car ctrl_int_fwd: TGAGGACCGCAGTCCCTAAT |
| Carctrl_int_rev: ACACAGTTCCTCGGCCCATA |
| ctrl del exon2 (fw): TGACAGCTATGCTAACACTAG |
| ctrl del exon2 (rev): TCAGGGCGTGGAAATGATAG |
| Car_151_fwd: GGAGGACCAGATCTCCCTTC |
| Car_151_rev: CTGGAGATGCAGTCCTTTCAG |

**TableS7**: Sequences of primers used for RT-qPCR

| **Primers used for qPCR** | | |
| --- | --- | --- |
| **Gene name** | **Forward** | **Reverse** |
| ***Actin*** | TCATCACTATTGGCAACGAGC | AGTTTCATGGATGCCACAGG |
| ***36b4*** | AGATTCGGGATATGCTGTTGG | AAAGCCTGGAAGAAGGAGGTC |
| ***Nanog*** | TACTGAGATGCTCTGCACAG | GACTGGTAGAAGAATCAGGG |
| ***Id4*** | GAGACTCACCCTGCTTTGCT | ATGCTGTCACCCTGCTTGTT |
| ***Gfra1*** | TACCACCAGCATGTCCAATGAA | GTAGCTGTGCTTGGCTGGAACT |
| ***Foxo1*** | CAGCAAATCAAGTTATGGAGGA | TATCATTGTGGGGAGGAGAGTC |
| ***Gata2*** | CGATACCCACCTATCCCTCCT | GTGGCACCACAGTTGACACA |
| ***Tex19.1*** | CCTCCCTGGTGTTCAGTGCTT | ACTTGCTCGATGGACTGAGG |
| ***Gadd45b*** | AACGCGGTTCAGAAGATGCAG | TTCATCAGTTTGGCCGCCTC |
| ***Cdh1*** | ACGTCCATGTGTGTGACTGTG | AGGAGCAGCAGGATCAGAATC |
| ***Thy1*** | GGCTGCTTCTGATTATTTAGTTTGTTC | ACCCACCATACGCCCTTATG |
| ***Car*** | TGGTCCCATCTGTCCGTTTG | AAGATGCTTTCTCTGCCCGC |
|  |  |  |
